# Preparation of 6-Monohalo-β-cyclodextrin Derivatives with Selectively Methylated Rims via Diazonium Salts

**DOI:** 10.1021/acsomega.3c01950

**Published:** 2023-07-27

**Authors:** Konstantin Lebedinskiy, Jindřich Jindřich

**Affiliations:** Department of Organic Chemistry, Faculty of Science, Charles University, Hlavova 8, CZ-128 43 Prague, Czech Republic

## Abstract

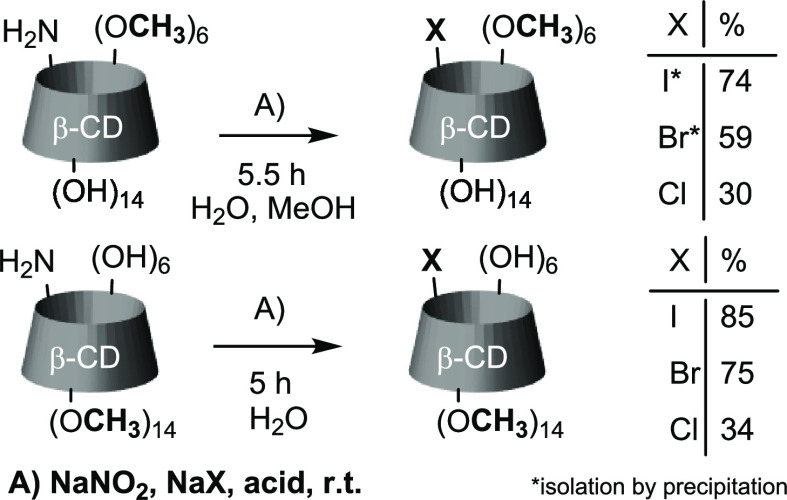

A series of 6-monohalo (Cl, Br, and I) β-cyclodextrin derivatives with various types of methylations were synthesized via a diazotization/nucleophilic displacement reaction from the corresponding methylated cyclodextrin amines. All four starting compounds (6^A^-amino-6^A^-deoxy derivatives of native β-CD, per-6-*O*-methyl-, per-2,3-*O*-methyl-, and per-2,3,6-*O*-methyl-β-CD) were found to have different reactivities under the same reaction conditions. Unsubstituted and fully per-O-methylated cyclodextrin amines undergo fast transformation, giving lower yields of the monohalogenated product. The selectively methylated cyclodextrin amines react remarkably slower and provide almost complete conversion into the desired monohalogenated compound. A pure product was, in several cases, successfully isolated with simple purification techniques (extraction and precipitation), allowing large-scale preparations. This new method opens the way for preparing poorly investigated monofunctionalized selectively methylated cyclodextrins.

## Introduction

Cyclodextrins (CDs) are cyclic oligosaccharides widely applied in many branches of industry because of their ability to form inclusion complexes with various organic molecules.^1^ The abundance of hydroxyl groups on cyclodextrins’ primary and secondary rims ensures plenty of possible ways to derivatize them.^2^ However, the most common strategy revolves around the functionalization of only one hydroxyl on position 6 of the glucose unit and subsequent transformations of this group.^3^ This functional group is usually a leaving group (tosylate, halide, 2,3,6-trimethylphenylsulfonate, etc.), amine, or azide. However, if any other reactions of unsubstituted hydroxyl groups of cyclodextrin are required, the leaving group may degrade as these reactions are commonly conducted in strongly basic conditions.^4^ That is why such processes are more convenient for CD azides.^5^ The most common ways to modify the rest of the hydroxyl groups are alkylations, such as hydroxypropylation and methylation. Such transformation affords an increased solubility and sometimes enhanced complexation affinity to a particular guest.^6^ For practical purposes, randomly alkylated cyclodextrins with some unreacted hydroxyl groups are often synthesized, as, for example, randomly methylated CDs with 2/3 alkylated positions reach the maximum water solubility.^2^ However, the blended nature of such compounds does not allow their use, for instance, in enzyme mechanism^7^ or chemosensor^8^ studies. Thus, preparing CD derivatives with selectively substituted hydroxyl groups is of some interest.^9,10^

There are well-established procedures for preparing methylated CDs with selectively substituted positions,^11^ but information about the monofunctionalization of such compounds is quite scarce. There are several strategies available: mono-6-O-protection with *tert*-butyldimethylsilyl,^12^*tert*-butyldiphenylsilyl,^13^ or trityl^14^ group followed by methylation-deprotection sequence; monoalkylation of selectively methylated CD,^15^ monodemethylation,^16^ monotosylation of the secondary-rim methylated CD,^17^ or direct methylation of mono-6-*O*-tosylated CD.^4,18^ Recently, we have developed new procedures for preparing mono-azido β-cyclodextrin derivatives with selectively methylated rims.^19^ The cyclodextrin azides are stable and easy-to-handle compounds that enable selective and efficient reactions.^20,21^ However, they do not allow reactions with nucleophiles, such as the widely used mono-6-*O*-tosyl-β-CD,^3^ which might be desirable in some cases. For example, the azide cannot be converted directly into ether or thioether.

Diazonium salts are valuable intermediates in organic synthesis, particularly in the derivatization of aromatic compounds.^22,23^ According to crystallography studies, the bond length between nitrogen atoms is almost the same as in the nitrogen molecule,^24^ thus enabling the outstanding electrophilic properties of diazonium compounds accompanied by the evolution of N_2_. On the other hand, the labile -N≡N^+^ group justifies the typical instability of the molecule, significantly limiting its application area almost exclusively to aromatic systems where the conjugation with an aromatic ring provides additional stabilization to the molecule.^25^ The application of aliphatic diazonium salts is restricted because of the expected absence of stabilization factors and the resulting lack of stability. The typical products of diazotization of aliphatic amines are the corresponding alkenes and alcohols because the process is typically carried out in water media. At the same time, the diazonium salt is not detected in the mixture. The main exclusions from this rule are the compounds that cannot form stable alkenes or carbocations like, for example, methanediazonium or bridged cyclic diazoniumalkanes, and those substances that have a strong electron-withdrawing group on the carbon atom bearing the diazonium moiety.^26^ Though some reports showed promising results,^27,28^ the diazotization of aliphatic amines is still rare and somewhat exotic. The best results have been achieved with one-pot processes when an aliphatic diazonium salt is formed *in situ* for the esterification of carboxylic acid^29^ and the alkylation of triazoles.^27^ Authors of these works attribute the good utility of their methods to the formation of a diazonium ion pair with the corresponding nucleophile that rapidly yields the desired product before other unwanted processes occur. Few works on diazo carbohydrate derivatives, containing either a stabilized benzyl group^30,31^ or unstabilized diazo group,^32^ have been published, and the usability of the diazo group for nucleophilic substitution with an acid was presented.

Our work develops efficient synthetic methods for derivatizing selectively methylated β-cyclodextrin derivatives with one functional group. To allow their reactions with nucleophiles, we were interested in creating a new strategy for converting the azido group of our compounds into a leaving group. Our initial plan was to reduce azide to amine and optimize the diazotization process of the amine to increase the content of the hydroxylated product, minimizing the number of other byproducts, as, for example, it was done by the Mukaiyama group for the total synthesis of taxol starting from (*S*)-serine.^33^ Then, we could replace the hydroxyl with a leaving group as other hydroxyls are protected or possess different reactivities than 6-OH. However, some reactions demonstrated remarkable effectiveness in directly substituting the amino group with halogen.

## Results and Discussion

### Synthesis of Monoamino-β-cyclodextrin Derivatives

**Scheme 1:**
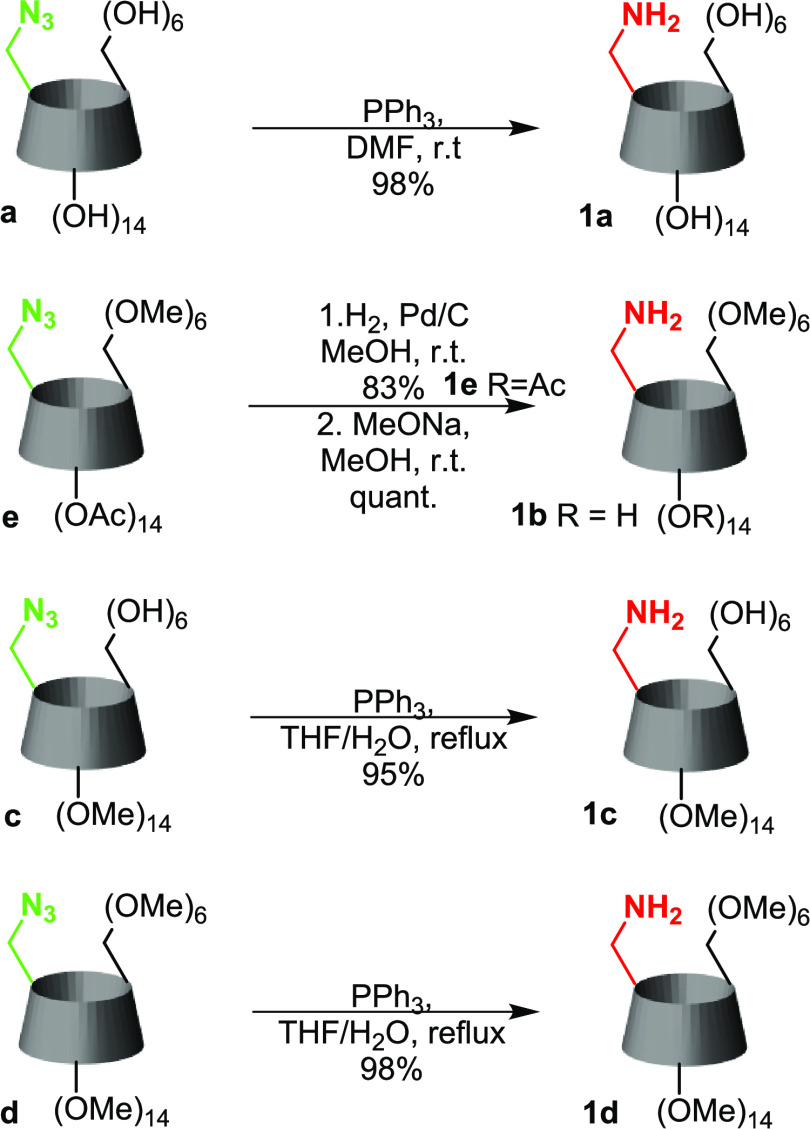
Transformation of Monoazido-CDs into the Corresponding Amines

We selected several 6-monoazido-β-cyclodextrin derivatives for our work— nonmethylated (a), selectively permethylated on the primary (b) or secondary (c) rim, and permethylated (d). Primary-rim methylated azide was also used in the peracetylated form (e). We first had to convert the chosen CD azides into the corresponding monoamino compounds (Scheme 1). Reducing the azido group by triphenylphosphine and subsequent hydrolysis of the iminophosphorane^34^ was the most convenient way. Still, in the case of the primary-rim methylated cyclodextrin, we reduced the azido group by hydrogenation in the presence of Pd/C^35^ because the hydrolysis of the intermediate resulted in the formation of the amide alongside the product, thus lowering the yield. One part of compound **1e** was kept for separate experiments with monohalogenation, and another part was transformed into **1b** by base-catalyzed cleavage of acetyls.

### Synthesis of Monohalo-β-cyclodextrin Derivatives

**Figure 1:**
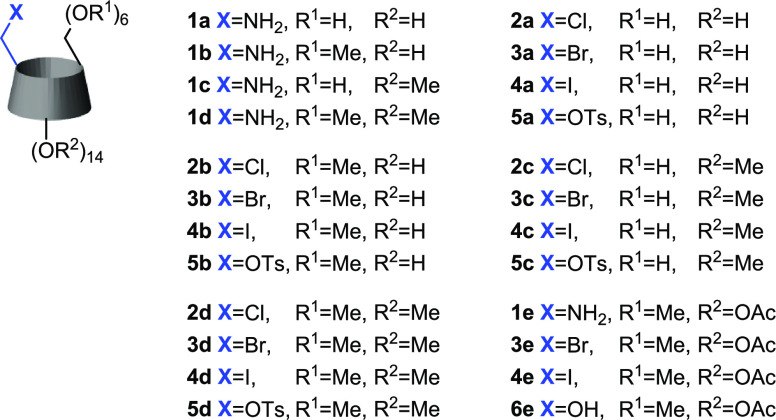
Numbering of Amino-CDs and Halo-CDs Used in This Work.

**Scheme 2:**
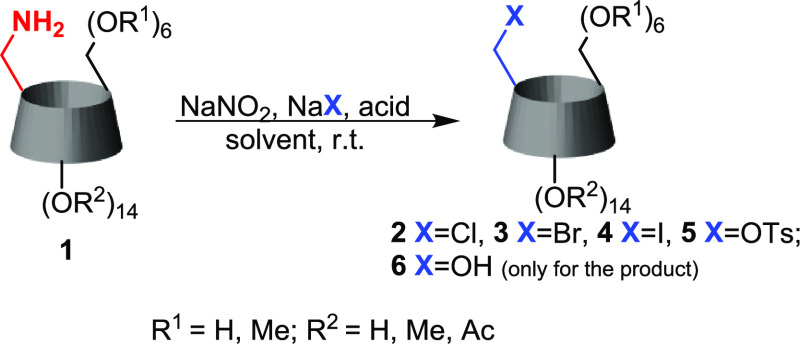
General Scheme of the Diazotization of Amino-CDs

The obtained cyclodextrin amines were then transformed into the corresponding monohalides by the diazotization/nucleophilic substitution procedure. The general scheme of the reaction is presented in Scheme 2. The numbering of all utilized starting compounds and obtained products is shown in Figure 1. For a start, we tested the common diazotization procedure for aromatic amines that includes the treatment of water-based mixtures of amines and sodium nitrite by a strong acid (TsOH) at 5 °C and the subsequent addition of a halide at room temperature.^23^ Later, further iterations of this procedure led us to methods described in detail in the corresponding sections below.

### Two-Step Transformation of **1e**

**Scheme 3:**
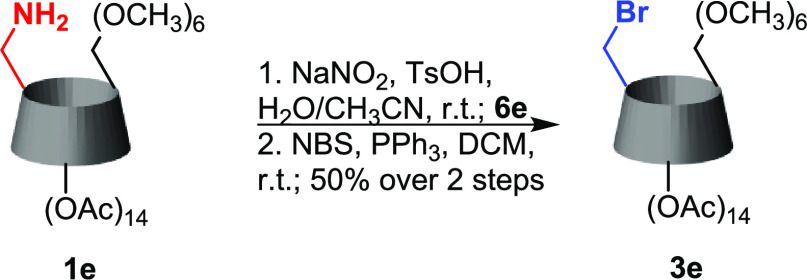
Two-Step Bromination of **1e**

Different conditions were tested to turn the amino group into hydroxyl. To promote the hydrolysis of the diazonium salt, the most effective strategy was to use a strong acid (TsOH) and acetonitrile/water mixture instead of pure water as the solvent. It seems that water molecules provide additional stabilization of the unstable intermediate, and in less polar media, it becomes prone to hydrolysis even more than usual. This method gave us a pretty good conversion of **1e** into the mono-hydroxylated substance **6e** that was extracted from the reaction mixture by chloroform together with several minor byproducts. Then, the crude mixture was put directly into the bromination process, which gave us the pure desired monobrominated product **3e** after column chromatography (Scheme 3). A relatively low yield (50%) is a consequence of these byproducts, whose amount after the first stage was significant.

### Monohalogenation of **1c** (6^A^-Amino-6^A^-deoxy-per-2,3-*O*-methyl-β-CD)

Our attempts to apply the diazotization conditions using TsOH to other chosen CD amines yielded different results, as **1a** and **1d** produced a significant amount of the monotosylated compound and the hydroxylated one. The *p*-toluenesulfonate ion can act effectively as a nucleophile in the reaction despite its low nucleophilic power and the unfavored molar ratio TsO^–^/H_2_O. **1c** stands out of all the compounds that we worked with because it formed no hydroxylated product at the given reaction conditions. Instead, it yielded a mixture of traces of the mono-tosylated compound **5c**, the starting material **1c**, and an unknown product whose *R*_F_ on a TLC plate was even lower than the starting amine. We consider the substance to be a relatively stable diazonium salt because adding sodium bromide to the reaction mixture pushed the reaction further, causing intense gas release and converting the third compound into monobromide **3c**. In the end, starting material **1c** was transformed into a mixture of monotosylate and monobromide, and a small part remained unreacted.

Optimizing the reaction conditions, we eventually devised two general approaches for all reactions based on the acid used to promote diazotization. In the first approach, a portion of a halide sodium salt was introduced to the reaction mixture as a source of nucleophiles before the addition of the acidic solution began. *p*-Toluenesulfonic acid is ruled out from the procedure and replaced with acetic acid. Though all halide anions are much stronger nucleophiles than the tosylate anion, the latter produces a small amount of monotosylate regardless of its lower strength. This phenomenon makes the behavior of the CD different from the aromatic diazonium salts, as the aromatic molecules rarely react with the tosylate ion.^23,36^ Oppositely, even stabilized aliphatic diazonium salts can react with even weaker nucleophiles, like fluorosulfate.^26^ Thus, the CDs obey the general rule. Acetic acid is acidic enough to promote diazotization but does not produce an abundance of acetate anions that may cause unwanted acetylation. The accumulation of acetate anions in the reaction is balanced by a vast excess of halides in the mixture. Moreover, a weak acid does not catalyze the undesired oxidation of a halide ion according to the following equation (eq 1) as much as we observed with strong acids:
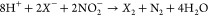
1

The direct one-step monohalogenation of **1c** in the presence of AcOH gave us the corresponding bromo (**3c**) and iodo (**4c**) products. Still, it worked worse for the chloro derivative (**2c**) because of chloride’s weaker nucleophilicity. So, for monochlorination, we treated the mixture of **1c**, sodium nitrite, and sodium chloride with a hydrochloric acid solution to avoid a competitive reaction with the acetate ion. Thus, the second approach that we used in several experiments is the treatment of a reaction mixture with hydrogen halide solution instead of acetic acid. This procedure is suitable when the competitive process with an acidic anion is significant. The monohalogenation of **1c** demonstrates the direct correlation between the isolated yield and the nucleophilic strength of the halide anion. As we also already mentioned, the treatment of **1c** by *p*-toluenesulfonic acid produces only traces of the tosylate **5c**. Our efforts to optimize the reaction conditions were unsuccessful, so this compound was not isolated in its pure form.

The preparation of **4c** deserves additional clarification because the oxidation process (eq 1) considerably reduces the conversion rate. The reaction is not finished before nitrite and iodide cease from the mixture, so additional equivalents of both reagents are required. Mild acidic conditions helped us to slow down the unwanted process, but to hamper it even more, we replaced water media with water/methanol solution. This change does not alter much the reaction rate or the composition of the products but allows complete conversion without loading additional equivalents of the reagents. Compounds **2c**–**4c** can be easily purified by simple extraction with chloroform; the organic phase with the monoiodo product **4c** was washed with sodium thiosulfate solution to remove molecular iodine.

### Monohalogenation of **1d** (6^A^-Amino-6^A^-deoxy-per-2,3,6-*O*-methyl-β-CD)

The optimized procedures discussed in the previous section were also applied to other entries. Thus, we utilized HX (X = Cl and Br for **2d** and **3d**) as the acid and the additional source of nucleophiles to prevent the undesired side processes. For the synthesis of **4d**, we used NaI/AcOH combination, and again the water/methanol mixture was picked as the solvent. Unlike **1c**, compound **1d** in some processes demonstrates the tendency to form the hydroxylated product, the whole reaction takes less time (up to 1 h for entries with **1d** against 5 h for entries with **1c**), and no starting material is observed when it is finished. The ratio between halogenated and hydroxylated products depends strongly on the nucleophilic power of halide. The iodide ion converts the amine into **4d** completely, the bromide ion gives a reasonable yield of **3d**, and finally, the chloride provides an almost equimolar mixture of **2d** and the hydroxylated products. All these estimations are based on TLC analysis and the height of peaks on MS without precise quantification; the isolated yields are expectedly smaller. Pure **4d** was obtained by chloroform extraction—thiosulfate washing with quantitative yield; other entries with **1d** required column chromatography.

The only published compounds analogous to the prepared permethylated monohalides with a described preparation procedure are 6-monotosyl-per-2,3,6-*O*-methyl-β-CD (**5d**)^4,18^ and the corresponding triflate derivative,^13^ prepared in two steps from the tosylate, that showed a much higher reactivity toward nucleophiles than the tosylate.^37^ Thus, the triflate can be considered the most suitable intermediate for preparing 6-monosubstituted permethylated CDs, and our monohalides might not be advantageous for preparing these types of CD derivatives.

### Monohalogenation of **1a** (6^A^-Amino-6^A^-deoxy-β-CD)

The preparation of monohalogenated CDs **2a**, **3a**, and **4a** via the diazonium salt does not have much practical value; nevertheless, we synthesized these compounds along with the well-known **5a**(38) from compound **1a** to estimate its reactivity and compare it to other studied amines. Surprisingly, we got a complete set of non-methylated CDs with chloro, bromo, iodo, and tosyl groups (**2a**–**5a**). Entries with **1a** demonstrate the most unfavorable product/byproduct ratios among all studied compounds, even with the strongest nucleophiles. Unexpectedly, all the reactions with the non-methylated CD had comparable yields regardless of the nucleophile’s strength.

### Monohalogenation of **1b** (6^A^-Amino-6^A^-deoxy-per-6-*O*-methyl-β-CD) and **1e**

The monohalogenation of compound **1b** was an essential reaction for us since there are not many monoderivatized primary-rim methylated β-CD derivatives known in the literature.^19^ The preparation of monoazido- and monoamino- compounds supplemented by monohalides can reveal many possibilities for further reactions of primary-rim methylated CDs and thus expand their potential applications.

All entries with **1b** (Table 1) had to be carried out in the methanol/water mixture because of solubility issues. Like compound **1c**, it reacts slower (around 5–6 h) than **1a** and **1d**, and the side process (eq 1) consumes a considerable amount of nitrite and halide, reducing the starting material’s conversion and yield. Unfortunately, a partial hydrolysis of the diazonium salt has been detected for all entries with **1b**, though it is significantly smaller than with **1a** and **1d**. All per-6-*O*-methyl-6-monohalo compounds are poorly soluble in the reaction mixture, so they can be purified by precipitation and recrystallization. The lowest conversion of the amine was observed in the synthesis of **2b** and **5b**, which, together with the considerable solubility of the latter compound, prevented us from isolating it in pure form. Compounds **3b** and **4b** are much less soluble in the water/methanol mixture than **2b** and **5b**, so the precipitation method for purification is much more reasonable for these substances.

**Table 1:** Synthesis of Monohalo-CDs

entry	starting material	product	procedure^a^	nucleophile/acid	solvent	time^c^ (h)	yield (%)
1	**1a**	**2a**	A	NaCl/HCl	H_2_O	0.5	28
2	**1a**	**3a**	A	NaBr/HBr	H_2_O		40
3	**1a**	**4a**	A	NaI/AcOH	H_2_O		43
4	**1a**	**5a**	A	TsONa/TsOH	H_2_O		38
5	**1b**	**2b**	A	NaCl/HCl	H_2_O/MeOH	5.5	30
6	**1b**	**3b**	A	NaBr/AcOH	H_2_O/MeOH		59
7	**1b**	**4b**	A	NaI/AcOH	H_2_O/MeOH		74
8	**1b**	**5b**	A	TsONa/TsOH	H_2_O/MeOH		-b
9	**1c**	**2c**	A	NaCl/HCl	H_2_O	5	34
10	**1c**	**3c**	A	NaBr/HBr	H_2_O		75
11	**1c**	**4c**	A	NaI/AcOH	H_2_O/MeOH		85
12	**1c**	**5c**	A	TsONa/TsOH	H_2_O		-b
13	**1d**	**2d**	A	NaCl/HCl	H_2_O	1	34
14	**1d**	**3d**	A	NaBr/HBr	H_2_O		72
15	**1d**	**4d**	A	NaI/AcOH	H_2_O/MeOH		99
16	**1d**	**5d**	A	TsONa/TsOH	H_2_O		40
17	**1e**	**3e**	B	-/TsOH	H_2_O/MeCN	1^d^	50
18	**1e**	**4e**	A	NaI/AcOH	H_2_O/MeOH	0.5	29

a A: according to Scheme 2, B: according to Scheme 3.

b The pure compound was not isolated.

c Average reaction time for the specified starting compound.

d For the first step, according to Scheme 3.

We also converted acetyl-protected compound **1e** into monoiodide **4e** by the same method (see Table 1). The yield and the difficulty of the purification do not allow us to consider this process as a handy alternative for other suggested protocols.

### Confirmation of Structures

The accepted opinion of those part of the scientific society that worked with aliphatic diazonium salts is that the instability of these intermediates makes the reaction outcome unpredictable since their degradation leads to unstabilized carbocations prone to undergo fragmentations and rearrangements.^26^ However, those products that have been synthesized before by using conventional methods perfectly matched our compounds prepared according to the diazotization protocol. Mass spectroscopy identified only one substance with a molecular weight equal to the desired monohalo-CD derivative for all our compounds. We also used the ^13^C-NMR chemical shift of the atom directly connected to a halogen as a reliable confirmation of the structure. The spectra of all obtained products contained only one signal corresponding to halogenated carbon in the ranges of 45.4–45.7 ppm for monochloro-CDs, 33.4–35.4 ppm for monobromo-CDs, and 6.8–10.3 ppm for monoiodo-CDs; moreover, the DEPT spectrum unambiguously proves the connection of C-6 to the halogen.

## Conclusions

We have successfully applied the classical diazotization procedure with slight alternations to prepare a set of β-cyclodextrin derivatives with one leaving group on its primary side and different degrees of methylation of its rims. In most cases, the prepared compounds have not been reported yet in the literature. The studied CD amines with a different degree of methylation possess different reactivities under the same reaction conditions. Non-methylated, permethylated, and permethylated-acetylated CDs can be quickly and completely transformed into a mixture of halogenated and hydroxylated products. The ratio between halogenated and hydroxylated products for the permethylated CD and the percentage of the starting amine converted into the product are directly related to the nucleophile’s strength. Oppositely, a nucleophile’s strength contributes little to the reaction outcome with the non-methylated and permethylated-acetylated CDs. On the other hand, both selectively methylated CD amines require more reaction time and form monohalides predominately with all the tested halides. The overall effectiveness of the amine’s displacement by halide is inversely related to the reaction rate.

The obtained compounds, especially per-6-*O*-methyl-CD and per-2,3-*O*-methyl-CD derivatives, are valuable intermediates for further reactions of the selectively methylated cyclodextrins with other nucleophiles, such as amines and thiols, opening new possibilities for preparing selectively methylated cyclodextrin-based organocatalysts, selective supramolecular hosts, or chemosensors.

Our current project dealing with CD chemosensors confirmed the usability of the selectively methylated monohalo-CDs for binding to amine linkers. The study of their use for preparing cyclodextrin-drug conjugates with a controlled release of the active compound is underway.

## Experimental Section

### Materials

β-Cyclodextrin was purchased from Wako Chemicals; the other chemicals were bought from Merck. SiliaFlash P60 40-63 μm from SiliCycle was used for column chromatography. The solvents were supplied by Penta and were distilled before use. The course of the reactions was followed on TLC Silica gel 60F_254_ bought from Merck.

### Methods

Low-resolution mass spectra were measured with a Shimadzu LCMS-2020. The drying and nebulizer gas was nitrogen. High-resolution mass spectra were measured with an Agilent Technologies 6530 Accurate-Mass Q-TOF LC/MS. Samples were ionized by electrospray technique (ESI) and detected by quadrupole or TOF. ^1^H, ^13^C, and 2D-NMR spectra were measured on a Bruker Avance III HD 400. For TLC detection of CDs, we charred a TLC plate with 50% sulfuric acid water solution at 250 °C. The prepared compounds were dried at reduced pressure to constant weight.

### Synthesis

The starting azido-CDs (**a**, **e**, **c**, and **d**) were prepared according to the published procedures^5,19,34^ as well as compound **1a**.^39^

#### 6^A^-Amino-6^A^-deoxy-6^B-G^-hexa-*O*-methyl-cyclomaltoheptaose (**1b**)

Compound **1e** (500 mg; 0.277 mmol) was dissolved in 6 mL of methanol and treated with 1 mL of 1 M sodium methoxide solution for 1 h. After that, the reaction mixture was diluted by 10 mL of MeOH and neutralized with 300 mg of Amberlite 120 ion exchanger. Then, the resin was filtered off, and the pure product **1b** (365 mg; 99% yield) was obtained after drying.

^1^H NMR (400 MHz, DMSO-*d*_6_): 5.58–6.07 ppm (14H; 2-OH, 3-OH), 4.68–4.96 ppm (7H; H1), 3.43–3.87 ppm (26H; H3, H5, H6), 3.10–3.43 ppm (32H; H2, H4, 6-OCH_3_, overlapped with H_2_O), 2.79–2.90 ppm (2H; H6^A^); ^13^C-DEPT NMR (100 MHz, DMSO-*d*_6_): 102.4–102.7 ppm (C1), 82.3–83.9 ppm (C4), 72.8–73.5 ppm (C2, C5), 71.0–71.6 ppm (C6^B,C,D,E,F,G^), 70.6–70.9 ppm (C3), 58.4–58.9 ppm (6-CH_3_), 41.7 ppm (C6^A^). HRMS (ESI): *m/z* calcd for C_48_H_84_NO_34_^+^ [M + H^+^]: 1218.48693 found: 1218.48380. IR (KBr): 3303, 2924, 2817, 1663, 1558, 1367, 1151, 1032, 858 cm^–1^.

#### 6^A^-Amino-6^A^-deoxy-2^A-G^,3^A-G^-tetradeca-*O*-methyl-cyclomaltoheptaose (**1c**)

For the preparation of compound **1c**, the starting azide (0.371 mmol) was mixed with triphenylphosphine (0.25 g; 0.96 mmol) in a THF/H_2_O 8/1 (8 mL) mixture at room temperature, and after 1 h, the reaction mixture was refluxed overnight. Then, it was diluted with an excess of 1 M hydrochloric acid, and all organic byproducts were extracted by diethyl ether. Then, the acidic solution was neutralized by sodium carbonate, and the pure product was extracted by chloroform and dried (0,47 g; 95% yield). The spectra of the obtained compound match the literature data.^17^

#### 6^A^-Amino-6^A^-deoxy-2^A-G^,3^A-G^,6^B-G^-icosa-*O*-methyl-cyclomaltoheptaose (**1d**)

Compound **1d** was prepared similar to compound **1c**. From the starting azide (0.5 g; 0.346 mmol), we obtained the product (0.49 g; 98% yield). The spectra of the product match the literature data.^5^

#### 2^A-G^,3^A-G^-Tetradeca-*O*-acetyl-6^A^-amino-6^A^-deoxy-6^B-G^-hexa-*O*-methyl-cyclomaltoheptaose (**1e**)

The starting azide (1 g; 0.54 mmol) and 10% wt. Pd/C (100 mg) were stirred in methanol (12 mL), and the flask was connected to a balloon filled with hydrogen. After the night, the suspension was filtered on celite, concentrated, and chromatographed on a silica gel column with a chloroform/methanol mixture (0.77 g; 78% yield).

^1^H NMR (400 MHz, CDCl_3_): 5.21–5.48 ppm (7H; H3), 5.01–5.21 ppm (7H; H1), 4.71–4.95 ppm (7H; H2), 3.66–4.19 ppm (21H; H4, H5, H6), 3.51–3.66 ppm (7H; H6), 3.34–3.51 ppm (18H; 6-OCH_3_), 1.95–2.23 ppm (42H; C(O)-CH_3_); ^13^C-DEPT NMR (100 MHz, CDCl_3_): 96.0–97.2 ppm (C1), 75.1–77.3 ppm (C5), 70.1–71.9 ppm (C2, C3, C4, C6), 59.3 ppm (6-OCH_3_), 20.7–20.9 ppm (C(O)-CH_3_). HRMS (ESI): *m/z* calcd for C_76_H_108_NaO_48_^+^ [M + Na^+^]: 1828.6168 found: 1828.61593. IR (KBr): 2933, 2816, 1741, 1435 1369, 1215, 889, 602, 467 cm^–1^.

#### General Procedure for the Monohalogenation of an Amino-CD (**A**)

An amino-CD (0.1 mmol) was dissolved in 2 mL of solvent (see Table 1) together with sodium nitrite (35 mg; 0.5 mmol) and sodium halide (0.8 mmol). The reaction mixture was stirred slowly, and a portion of acid (see Table 1; 0.4 mmol) dissolved in 0.2 mL of water was added dropwise to the solution. The progress of the reaction was monitored by TLC. The purification process was different depending on the starting compound. For entries with **1a**, the reaction mixture was poured into acetone, the formed precipitate was filtered off and redissolved in water, and then, the product was chromatographed on a reverse-phase silica gel column with a water/methanol mixture. For entries with **1c**, **1d**, and **1e**, the product was extracted with chloroform and chromatographed on a standard silica gel column with a chloroform/methanol mixture if additional purification was required. For entries with **1b**, the formed precipitate was centrifuged and dissolved in 2 mL of a water/methanol 1/1 mixture at 60 °C. The compound was repeatedly recrystallized until completely purified.

#### 6^A^-Chloro-6^A^-deoxy-2^A-G^,3^A-G^-tetradeca-*O*-methyl-cyclomaltoheptaose (**2c**)

Starting from amino-CD **1c** (200 mg; 0.152 mmol), using the general procedure A, the compound was isolated as a colorless solid in 34% yield (73 mg).

^1^H NMR (400 MHz, CDCl_3_): 5.06–5.23 ppm (7H; H1), 4.18–4.26 ppm (1H; H6), 3.43–4.08 ppm (76H; H3, H4, H5, H6, 2-CH_3_, 3-CH_3_), 3.12–3.29 ppm (7H, H2); ^13^C-DEPT NMR (100 MHz, CDCl_3_): 98.2–99.0 ppm (C1), 79.1–82.1 ppm (C2, C3, C4), 70.9–72.7 ppm (C5), 61.6–62.1 ppm (C6^B,C,D,E,F,G^), 61.2–62.1 ppm (3-O-CH_3_), 58.4–58.8 ppm (2-O-CH_3_), 45.7 ppm (C6^A^). HRMS (ESI): *m/z* calcd for C_56_H_98_ClO_34_^+^ [M + H^+^]: 1349.56225 found: 1349.56105. IR (KBr): 3400, 2927, 2833, 1635, 1367, 1105, 1016, 852, 546 cm^–1^.

#### 6^A^-Bromo-6^A^-deoxy-2^A-G^,3^A-G^-tetradeca-*O*-methyl-cyclomaltoheptaose (**3c**)

Starting from amino-CD **1c** (150 mg; 0.112 mmol), using the general procedure A, the compound was isolated as a colorless solid in 75% yield (117 mg).

^1^H NMR (400 MHz, CDCl_3_): 5.05–5.26 ppm (7H; H1), 4.27–4.51 ppm (6H; 6-OH), 3.42–4.09 ppm (77H; H3, H4, H5, H6, 2-CH_3_, 3-CH_3_), 3.12–3.29 ppm (7H, H2); ^13^C-DEPT NMR (100 MHz, CDCl_3_): 98.1–99.0 ppm (C1), 78.8–82.8 ppm (C2, C3, C4), 70.4–72.8 ppm (C5), 61.5–62.0 ppm (C6^B,C,D,E,F,G^), 61.1–61.4 ppm (3-O-CH_3_), 58.3–59.0 ppm (2-O-CH_3_), 35.3 ppm (C6^A^). HRMS (ESI): *m/z* calcd for C_56_H_98_BrO_34_^+^ [M + H^+^]: 1393.51174 found: 1393.50696. IR (KBr): 3411, 2929, 2831, 1632, 1446, 1367, 1105, 1014, 852 cm^–1^.

#### 6^A^-Deoxy-6^A^-iodo-2^A-G^,3^A-G^-tetradeca-*O*-methyl-cyclomaltoheptaose (**4c**)

Starting from amino-CD **1c** (120 mg; 0.09 mmol), using the general procedure A, the compound was isolated as a brownish solid in 85% yield (110 mg).

^1^H NMR (400 MHz, CDCl_3_): 5.06–5.22 ppm (7H; H1), 3.42–4.01 ppm (77H; H3, H4, H5, H6, 2-CH_3_, 3-CH_3_), 3.15–3.26 ppm (7H, H2); ^13^C-DEPT NMR (100 MHz, CDCl_3_): 97.7–98.7 ppm (C1), 77.1–84.9 ppm (C2, C3, C4), 70.1–72.8 ppm (C5), 61.7–62.1 ppm (C6^B,C,D,E,F,G^), 61.3–61.7 ppm (3-O-CH_3_), 58.3–59.0 ppm (2-O-CH_3_), 10.1 (C6^A^). HRMS (ESI): *m/z* calcd for C_56_H_98_IO_34_^+^ [M + H^+^]: 1441.49787 found: 1441.49614. IR (KBr): 3408, 2927, 2831, 1663, 1367, 1014,852, 750, 546 cm^–1^.

#### 6^A^-Chloro-6^A^-deoxy-2^A-G^,3^A-G^,6^B-G^-icosa-*O*-methyl-cyclomaltoheptaose (**2d**)

Starting from amino-CD **1d** (140 mg; 0.1 mmol), using the general procedure A, the compound was isolated as a colorless oil in 34% yield (48 mg).

^1^H NMR (400 MHz, CDCl_3_): 5.08–5.19 ppm (7H; H1), 3.45–4.06 ppm (77H; H3, H4, H5, H6, 2-CH_3_, 3-CH_3_), 3.35–3.45 ppm (6-O-CH_3_), 3.16–3.24 ppm (7H, H2); ^13^C-DEPT NMR (100 MHz, CDCl_3_): 98.3–99.4 ppm (C1), 79.9–82.5 ppm (C2, C3, C4), 71.4 ppm (C6^B,C,D,E,F,G^), 71.0 ppm (C5), 61.6 ppm (3-O-CH_3_), 58.2–59.1 ppm (2-O-CH_3_, 6-O-CH_3_), 45.4 ppm (C6^A^). HRMS (ESI): *m/z* calcd for C_62_H_110_ClO_34_^+^ [M + H^+^]: 1433.65615 found: 1433.66255. IR(KBr): 2929, 2835, 1456, 1367, 1138, 1034, 968, 754, 544 cm^–1^.

#### 6^A^-Bromo-6^A^-deoxy-2^A-G^,3^A-G^,6^B-G^-icosa-*O*-methyl-cyclomaltoheptaose (**3d**)

Starting from amino-CD **1d** (500 mg; 0.34 mmol), using the general procedure A, the compound was isolated as a yellowish oil in 72% yield (360 mg).

^1^H NMR (400 MHz, CDCl_3_): 5.12–5.18 ppm (7H; H1), 4.01–4.06 ppm (1H; C6), 3.76–3.96 ppm (14H; H5, H6), 3.44–3.73 ppm (63H; H3, H4, 2-CH_3_, 3-CH_3_), 3.37–3.44 ppm (6-O-CH_3_), 3.17–3.24 ppm (7H, H2); ^13^C-DEPT NMR (100 MHz, CDCl_3_): 98.4–99.2 ppm (C1), 80.1–83.5 ppm (C2, C3, C4), 71.2–71.6 ppm (C6^B,C,D,E,F,G^), 70.8–71.6 ppm (C5), 61.7 ppm (3-O-CH_3_), 58.5–59.2 ppm (2-O-CH_3_, 6-O-CH_3_), 34.7 ppm (C6^A^). HRMS (ESI): *m/z* calcd for C_62_H_113_BrNO_34_^+^ [M + NH_4_^+^]: 1494.63219 found: 1494.63356. IR(KBr): 2974, 2924, 2833, 1456, 1138, 1018, 968, 752, 553 cm^–1^.

#### 6^A^-Deoxy-6^A^-iodo-2^A-G^,3^A-G^,6^B-G^-icosa-*O*-methyl-cyclomaltoheptaose (**4d**)

Starting from amino-CD **1d** (120 mg; 0.085 mmol), using the general procedure A, the compound was isolated as a yellowish oil in 99% yield (128 mg).

^1^H NMR (400 MHz, CDCl_3_): 5.11–5.19 ppm (7H; H1), 4.05–4.10 ppm (1H; H6), 3.71–3.93 ppm (14H; H5, H6), 3.46–3.71 ppm (63H; H3, H4, 2-CH_3_, 3-CH_3_), 3.34–3.45 ppm (6-O-CH_3_), 3.17–3.24 ppm (7H, H2); ^13^C-DEPT NMR (100 MHz, CDCl_3_): 98.1–99.6 ppm (C1), 79.7–82.0 ppm (C2, C3, C4), 70.4 ppm (C6^B,C,D,E,F,G^), 70.1–70.4 ppm (C5), 61.3 ppm (3-O-CH_3_), 58.4–59.2 ppm (2-O-CH_3_, 6-O-CH_3_), 9.1 (C6^A^). HRMS (ESI): *m/z* calcd for C_62_H_110_IO_34_^+^ [M + H^+^]: 1525.5918 found: 1525.5932. IR (KBr): 2976, 2925, 2833, 1456, 1365, 1136, 1030, 752, 552 cm^–1^.

#### 6^A^-Deoxy-2^A-G^,3^A-G^,6^B-G^-icosa-*O*-methyl-6^A^-*p*-toluenesulfonyl-cyclomaltoheptaose (**5d**)

Starting from amino-CD **1d** (250 mg; 0.177 mmol), using the general procedure A, the compound was isolated as a colorless oil in 40% yield (128 mg).

^1^H NMR (400 MHz, CDCl_3_): 7.79–8.1 ppm (2H; Ar-H), 7.39–7.41 ppm (2H; Ar-H), 5.01–5.22 ppm (7H; H1), 4.50–4.52 ppm (1H; CD), 4.15–4.21 ppm (1H; CD), 3.15–4.05 ppm (99H; H2, H3, H4, H5, H6, 2-O-CH_3_, 3-O-CH_3_, 6-O-CH_3_), 3.05 ppm (1H; H2), 2.48 ppm (3H; Ar-CH_3_). MS (ESI): *m/z* calcd for C_69_H_116_NaO_37_S^+^ [M + Na^+^]: 1591.68 found: 1592. Corresponds to the literature data.^4^

#### 6^A^-Chloro-6^A^-deoxy-cyclomaltoheptaose (**2a**)

Starting from amino-CD **1a** (300 mg; 0.265 mmol), using the general procedure A, the compound was isolated as a colorless solid in 28% yield (85 mg).

^1^H NMR (400 MHz, DMSO-*d*_6_): 5.64–5.88 ppm (14H; C1-OH, C2-OH), 4.78–4.91 ppm (7H; H1), 4.41–4.54 ppm (6H; C6-OH), 3.25–3.96 (42H; H2. H3, H4, H5, H6); ^13^C-DEPT NMR (100 MHz, DMSO-*d*_6_): 102.3–102.7 ppm (C1), 81.7–82.3 ppm (C4), 72.3–73.7 ppm (C2, C3, C5), 60.1–60.6 ppm (C6^B,C,D,E,F,G^), 45.7 ppm (C6^A^). HRMS (ESI): *m/z* calcd for C_42_H_70_ClO_34_^+^ [M + H^+^]: 1153.34315 found: 1153.33904. IR (KBr): 3288, 2924, 1645, 1412, 1151, 1020, 945, 849, 575 cm^–1^.

#### 6^A^-Bromo-6^A^-deoxy-cyclomaltoheptaose (**3a**)

Starting from amino-CD **1a** (600 mg; 0.529 mmol), using the general procedure A, the compound was isolated as a colorless solid in 40% yield (253 mg).

^1^H NMR (400 MHz, DMSO-*d*_6_): 5.61–5.80 ppm (14H; C1-OH, C2-OH), 4.79–4.89 ppm (7H; H1), 4.40–4.55 ppm (6H; C6-OH), 3.26–3.82 (42H; H2. H3, H4, H5, H6); ^13^C-DEPT NMR (100 MHz, DMSO-*d*_6_): 102.1–102.7 ppm (C1), 81.7–82.3 ppm (C4), 72.4–73.5 ppm (C2, C3, C5), 60.2–60.5 ppm (C6^B,C,D,E,F,G^), 33.4 ppm (C6^A^). HRMS (ESI): *m/z* calcd for C_42_H_73_BrNO_34_^+^ [M + NH_4_^+^]: 1214.31919 found: 1214.31877. IR (KBr): 3306, 2925, 1658, 1410, 1153, 1026, 945, 891, 575 cm^–1^. ^13^C NMR contradicts the data from ref (40); however, the spectrum of the compound prepared according to ref (40) matches the spectrum of the compound prepared using our procedure.

#### 6^A^-Deoxy-6^A^-iodo-cyclomaltoheptaose (**4a**)

Starting from amino-CD **1a** (300 mg; 0.265 mmol), using the general procedure A, the compound was isolated as a colorless solid in 43% yield (143 mg).

^1^H NMR (400 MHz, DMSO-*d*_6_): 5.60–5.81 ppm (14H; C1-OH, C2-OH), 4.78–4.89 ppm (7H; H1), 4.41–4.55 ppm (6H; C6-OH), 3.15–3.79 (42H; H2. H3, H4, H5, H6); ^13^C-DEPT NMR (100 MHz, DMSO-*d*_6_): 102.1–102.8 ppm (C1), 81.7–82.3 ppm (C4), 72.3–73.6 ppm (C2, C3, C5), 60.1–60.5 ppm (C6^B,C,D,E,F,G^), 10.3 ppm (C6^A^). HRMS (ESI): *m/z* calcd for C_42_H_70_IO_34_^+^ [M + H^+^]: 1245.27877 found: 1245.28134. IR (KBr): 3303, 2925, 1645, 1412, 1151, 1022, 945, 845, 575 cm^–1^. ^13^C NMR contradicts the data from ref (40); however, the spectrum of the compound prepared according to ref (40) matches the spectrum of the compound prepared in the current research.

#### 6^A^-*O*-*p*-Toluenesulfonyl-cyclomaltoheptaose (**5a**)

Starting from amino-CD **1a** (300 mg; 0.265 mmol), using the general procedure A, the compound was isolated as a colorless solid in 38% yield (128 mg).

^1^H NMR (400 MHz, DMSO-*d*_6_): 7.74 ppm (phenyl; 2H), 7.42 ppm (phenyl; 2H), 5.57–5.82 ppm (14H; 2-OH, 3-OH), 4.74–4.86 ppm (7H; H1), 4.11–4.55 ppm (6H; 6-OH), 3.17–3.72 ppm (42H; H2, H3, H4, H5, H6), 2.41 ppm (phenyl-CH_3_). MS (ESI): *m/z* calcd for C_49_H_77_O_37_S^+^ [M + H^+^]: 1289.38 found: 1289. The data is in agreement with the literature.^38^

#### 6^A^-Chloro-6^A^-deoxy-6^B-G^-hexa-*O*-methyl-cyclomaltoheptaose (**2b**)

Starting from amino-CD **1b** (120 mg; 0.1 mmol), using the general procedure A, the compound was isolated as a colorless powder in 30% yield (37 mg).

^1^H NMR (400 MHz, DMSO-*d*_6_): 5.63–5.90 ppm (14H; 2-OH, 3-OH), 4.72–4.88 ppm (7H; H1), 3.81–4.03 ppm (2H; H6), 3.45–3.81 ppm (26H; H3, H5, H6), 3.15–3.43 ppm (32H; H2, H4, 6-OCH_3_, overlapped with H_2_O); ^13^C-DEPT NMR (100 MHz, DMSO-*d*_6_): 101.8 ppm (C1), 82.8 ppm (C4), 70.9–73.6 ppm (C2, C3, C5, C6^B,C,D,E,F,G^), 58.7 ppm (6-CH_3_), 45.6 ppm (C6^A^). HRMS (ESI): *m/z* calcd for C_48_H_83_ClO_34_^+^ [M + H^+^]: 1237.43705 found: 1237.43497. IR (KBr): 3300, 2925, 2817, 1635, 1331, 1151, 1078, 1036, 945 cm^–1^.

#### 6^A^-Bromo-6^A^-deoxy-6^B-G^-hexa-*O*-methyl-cyclomaltoheptaose (**3b**)

Starting from amino-CD **1b** (120 mg; 0.1 mmol), using the general procedure A, the compound was isolated as a yellowish powder in 59% yield (75 mg).

^1^H NMR (400 MHz, DMSO-*d*_6_): 5.60–5.87 ppm (14H; 2-OH, 3-OH), 4.72–4.86 ppm (7H; H1), 3.47–3.75 ppm (28H; H3, H5, H6), 3.19–3.39 ppm (32H; H2, H4, 6-OCH_3_, overlapped with H_2_O); ^13^C-DEPT NMR (100 MHz, DMSO-*d*_6_): 101.9 ppm (C1), 82.8 ppm (C4), 70.8–71.5 ppm (C2, C3, C5, C6^B,C,D,E,F,G^), 58.6 ppm (6-CH_3_), 35.2 ppm (C6^A^). HRMS (ESI): *m/z* calcd for C_48_H_83_BrO_34_^+^ [M + H^+^]: 1281.38654 found: 1281.38380. IR (KBr): 3311, 2925, 2816, 1558, 1412, 1151, 1080, 1036, 860 cm^–1^.

#### 6^A^-Deoxy-6^A^-iodo-6^B-G^-hexa-*O*-methyl-cyclomaltoheptaose (**4b**)

Starting from amino-CD **1b** (150 mg; 0.123 mmol), using the general procedure A, the compound was isolated as a brownish powder in 76% yield (124 mg).

^1^H NMR (400 MHz, DMSO-*d*_6_): 5.61–5.95 ppm (14H; 2-OH, 3-OH), 4.73–4.89 ppm (7H; H1), 3.43–3.78 ppm (28H; H3, H5, H6), 3.15–3.43 ppm (32H; H2, H4, 6-OCH_3_, overlapped with H_2_O); ^13^C-DEPT NMR (100 MHz, DMSO-*d*_6_): 102.9 ppm (C1), 82.3–86.9 ppm (C4), 70.3–73.6 ppm (C2, C3, C5, C6), 58.6–58.9 ppm (6-CH_3_), 10.0 ppm (C6^A^). HRMS (ESI): *m/z* calcd for C_48_H_86_INO_34_^+^ [M + NH_4_^+^]: 1346.3992 found: 1346.39756. IR (KBr): 3302, 2925, 2816, 1633, 1329, 1151, 1034, 945, 754 cm^–1^.

#### 2^A-G^,3^A-G^-Tetradeca-*O*-acetyl-6^A^-bromo-6^A^-deoxy-6^B-G^-hexa-*O*-methyl-β-cyclodexin (**3e**)

##### Two-Step Monobromination of **1e** (**B**)

Amine **1e** (400 mg; 0.222 mmol) and sodium nitrite (40 mg; 0.5 mmol) were dissolved in 6 mL of water/acetonitrile 2/1 mixture. A portion of TsOH (84 mg; 0.443 mmol) dissolved in 1 mL of water was dropwise added to the reaction mixture. After 4 h, the products were extracted with chloroform, and the organic phase was dried over anhydrous MgSO_4_. The concentrated crude product was dissolved in DCM (10 mL) with NBS (200 mg; 1.12 mmol) and triphenylphosphine (300 mg; 1.14 mmol) and left under stirring overnight. The pure product (210 mg; 51% yield) was obtained after silica gel chromatography as a colorless solid.

^1^H NMR (400 MHz, CDCl_3_): 5.24–5.50 ppm (7H; H3), 5.01–5.24 ppm (7H; H1), 4.75–4.91 ppm (7H; H2), 4.22–4.29 ppm (1H; H5), 3.80–4.15 ppm (20H; H4, H5 H6) 3.50–3.70 ppm (7H; H6), 3.37–3.45 ppm (18H; 6-OCH_3_), 1.97–2.13 ppm (42H; C(O)-CH_3_); ^13^C-DEPT NMR (100 MHz, CDCl_3_): 96.1–97.2 ppm (C1), 75.1–79.3 ppm (C5), 69.8–72.2 ppm (C2, C3, C4, C6^B,C,D,E,F,G^), 59.3 ppm (6-OCH_3_), 33.4 ppm (C6^A^), 20.9 ppm (C(O)-CH_3_). HRMS (ESI): *m/z* calcd for C_76_H_113_BrNO_48_^+^ [M + NH_4_^+^]: 1886.56099 found: 1886.55762. IR (KBr): 2933, 2816, 1741, 1437, 1369, 1215, 1026, 889, 540 cm^–1^.

#### 2^A-G^,3^A-G^-Tetradeca-*O*-acetyl-6^A^-deoxy-6^A^-iodo-6^B-G^-hexa-*O*-methyl-cyclomaltoheptaose (**4e**)

Starting from amino-CD **1e** (150 mg; 0.083 mmol), using the general procedure A, the compound was isolated as a colorless solid in 29% yield (46 mg).

^1^H NMR (400 MHz, CDCl_3_): 5.00–5.49 ppm (14H; H1, H3), 4.76–4.91 ppm (7H; H2), 3.74–4.27 ppm (20H; H4, H5, H6), 3.49–3.69 ppm (8H; H5, H6), 3.37–3.45 ppm (18H; 6-OCH_3_), 1.97–2.13 ppm (42H; C(O)-CH_3_); ^13^C-DEPT NMR (100 MHz, CDCl_3_): 95.9–97.3 ppm (C1), 74.8–77.2 ppm (C5), 69.3–72.7 ppm (C2, C3, C4, C6^B,C,D,E,F,G^), 59.3 ppm (6-OCH_3_), 20.8 ppm (C(O)-CH_3_), 6.8 ppm (C6^A^). HRMS (ESI): *m/z* calcd for C_76_H_110_IO_48_^+^ [M + H^+^]: 1917.5206 found: 1917.52342. IR (KBr): 2931, 2816, 1743, 1433, 1369, 1215, 1024, 889, 602 cm^–1^.

## Supporting Information Available

^1^H, ^13^C-DEPT, 2D-COSY, and 2D-HSQC NMR spectra (PDF)
