# Supplementary material for: Preparation of 6-Monohalo-β-cyclodextrin Derivatives with Selectively Methylated Rims via Diazonium Salts
Source: ACS Omega. 2023 Jul 27;8(31):28268–76. doi: 10.1021/acsomega.3c01950 (PMC10413458; doi:10.1021/acsomega.3c01950)
Supplement: Supplementary file 1 — ao3c01950_si_001.pdf [file ao3c01950_si_001.pdf]

Supporting Information

for

The preparation of 6-monohalo-β-cyclodextrin derivatives with selectively methylated rims via diazonium salts

Konstantin Lebedinskiy, Jindřich Jindrich*

Copies of NMR spectra of the prepared compounds

Contents

6^A-Amino-6^A-deoxy-6^{B-G}-hexa-*O*-methyl-cyclomaltoheptaose (**1b**) 4
Fig. S1 ^{1}H-NMR-400 MHz spectrum of **1b** in DMSO-d_6 at 25 °C 4
Fig. S2 ^{13}C-DEPT-101 MHz spectrum of **1b** in DMSO-d_6 at 25 °C 5
Fig. S3 2D-COSY spectrum of **1b** in DMSO-d_6 at 25 °C 6
Fig. S4 2D-HSQC spectrum of **1b** in DMSO-d_6 at 25 °C 7
6^A-Chloro-6^A-deoxy-2^{A-G},3^{A-G}-tetradeca-*O*-methyl-cyclomaltoheptaose (**2c**) 8
Fig. S5 ^{1}H-NMR-400 MHz spectrum of **2c** in $CDCl_3$ at 25 °C 8
Fig. S6 ^{13}C-DEPT-101 MHz spectrum of **2c** in $CDCl_3$ at 25 °C 9
Fig. S7 2D-COSY spectrum of **2c** in $CDCl_3$ at 25 °C 10
Fig. S8 2D-HSQC spectrum of **2c** in $CDCl_3$ at 25 °C 11
6^A-Bromo-6^A-deoxy-2^{A-G},3^{A-G}-tetradeca-*O*-methyl-cyclomaltoheptaose (**3c**) 12
Fig. S9 ^{1}H-NMR-400 MHz spectrum of **3c** in $CDCl_3$ at 25 °C 12
Fig. S10 ^{13}C-DEPT-101 MHz spectrum of **3c** in $CDCl_3$ at 25 °C 13
Fig. S11 2D-COSY spectrum of **3c** in $CDCl_3$ at 25 °C 14
Fig. S12 2D-HSQC spectrum of **3c** in $CDCl_3$ at 25 °C 15
6^A-Deoxy-6^A-iodo-2^{A-G},3^{A-G}-tetradeca-*O*-methyl-cyclomaltoheptaose (**4c**) 16
Fig. S13 ^{1}H-NMR-400 MHz spectrum of **4c** in $CDCl_3$ at 25 °C 16
Fig. S14 ^{13}C-DEPT-101 MHz spectrum of **4c** in $CDCl_3$ at 25 °C 17
Fig. S15 2D-COSY spectrum of **4c** in $CDCl_3$ at 25 °C 18
Fig. S16 2D-HSQC spectrum of **4c** in $CDCl_3$ at 25 °C 19
6^A-Chloro-6^A-deoxy-2^{A-G},3^{A-G},6^{B-G}-icosa-*O*-methyl-cyclomaltoheptaose (**2d**) 20

Fig. S17 ^{1}H-NMR-400 MHz spectrum of **2d** in $CDCl_3$ at 25 °C....20
Fig. S18 ^{13}C-DEPT-101 MHz spectrum of **2d** in $CDCl_3$ at 25 °C....21
Fig. S19 2D-COSY spectrum of **2d** in $CDCl_3$ at 25 °C....22
Fig. S20 2D-HSQC spectrum of **2d** in $CDCl_3$ at 25 °C....23
6^{A}-Bromo-6^{A}-deoxy-2^{A-G},3^{A-G},6^{B-G}-icosa-*O*-methyl-cyclomaltoheptaose (**3d**)....24
Fig. S21 ^{1}H-NMR-400 MHz spectrum of **3d** in $CDCl_3$ at 25 °C....24
Fig. S22 ^{13}C-DEPT-101 MHz spectrum of **3d** in $CDCl_3$ at 25 °C....25
Fig. S23 2D-COSY spectrum of **3d** in $CDCl_3$ at 25 °C....26
Fig. S24 2D-HSQC spectrum of **3d** in $CDCl_3$ at 25 °C....27
6^{A}-Deoxy-6^{A}-iodo-2^{A-G},3^{A-G},6^{B-G}-icosa-*O*-methyl-cyclomaltoheptaose (**4d**)....28
Fig. S25 ^{1}H-NMR-400 MHz spectrum of **4d** in $CDCl_3$ at 25 °C....28
Fig. S26 ^{13}C-DEPT-101 MHz spectrum of **4d** in $CDCl_3$ at 25 °C....29
Fig. S27 2D-COSY spectrum of **4d** in $CDCl_3$ at 25 °C....30
Fig. S28 2D-HSQC spectrum of **4d** in $CDCl_3$ at 25 °C....31
6^{A}-Chloro-6^{A}-deoxy-cyclomaltoheptaose(**2a**)....32
Fig. S29 ^{1}H-NMR-400 MHz spectrum of **2a** in DMSO-d_6 at 25 °C....32
Fig. S30 ^{13}C-DEPT-101 MHz spectrum of **2a** in DMSO-d_6 at 25 °C....33
Fig. S31 2D-COSY spectrum of **2a** in DMSO-d_6 at 25 °C....34
Fig. S32 2D-HSQC spectrum of **2a** in DMSO-d_6 at 25 °C....35
6^{A}-Bromo-6^{A}-deoxy-cyclomaltoheptaose (**3a**)....36
Fig. S33 ^{1}H-NMR-400 MHz spectrum of **3a** in DMSO-d_6 at 25 °C....36
Fig. S34 ^{13}C-DEPT-101 MHz spectrum of **3a** in DMSO-d_6 at 25 °C....37
Fig. S35 2D-COSY spectrum of **3a** in DMSO-d_6 at 25 °C....38
Fig. S36 2D-HSQC spectrum of **3a** in DMSO-d_6 at 25 °C....39
6^{A}-Deoxy-6^{A}-iodo-cyclomaltoheptaose (**4a**)....40
Fig. S37 ^{1}H-NMR-400 MHz spectrum of **4a** in DMSO-d_6 at 25 °C....40
Fig. S38 ^{13}C-DEPT-101 MHz spectrum of **4a** in DMSO-d_6 at 25 °C....41
Fig. S39 2D-COSY spectrum of **4a** in DMSO-d_6 at 25 °C....42
Fig. S40 2D-HSQC spectrum of **4a** in DMSO-d_6 at 25 °C....43
6^{A}-Chloro-6^{A}-deoxy-6^{B-G}-hexa-*O*-methyl-cyclomaltoheptaose (**2b**)....44
Fig. S41 ^{1}H-NMR-400 MHz spectrum of **2b** in DMSO-d_6 at 25 °C....44
Fig. S42 ^{13}C-DEPT-101 MHz spectrum of **2b** in DMSO-d_6 at 25 °C....45
Fig. S43 2D-COSY spectrum of **2b** in DMSO-d_6 at 25 °C....46

Fig. S44 2D-HSQC spectrum of **2b** in DMSO-d_6 at 25 °C....47
6^A-Bromo-6^A-deoxy-6^{B-G}-hexa-*O*-methyl-cyclomaltoheptaose (**3b**)....48
Fig. S45 H-NMR-400 MHz spectrum of **3b** in DMSO-d_6 at 25 °C....48
Fig. S46 ^{13}C-DEPT-101 MHz spectrum of **3b** in DMSO-d_6 at 25 °C....49
Fig. S47 2D-COSY spectrum of **3b** in DMSO-d_6 at 25 °C....50
Fig. S48 2D-HSQC spectrum of **3b** in DMSO-d_6 at 25 °C....51
6^A-Deoxy-6^A-iodo-6^{B-G}-hexa-*O*-methyl-cyclomaltoheptaose (**4b**)....52
Fig. S49 ^{1}H-NMR-400 MHz spectrum of **4b** in DMSO-d_6 at 25 °C....52
Fig. S50 ^{13}C-DEPT-101 MHz spectrum of **4b** in DMSO-d_6 at 25 °C....53
Fig. S51 2D-COSY spectrum of **4b** in DMSO-d_6 at 25 °C....54
Fig. S52 2D-HSQC spectrum of **4b** in DMSO-d_6 at 25 °C....55
2^{A-G},3^{A-G}-Tetradeca-*O*-acetyl-6^A-amino-6^A-deoxy-6^{B-G}-hexa-*O*-methyl-cyclomaltoheptaose (**1e**)....56
Fig. S53 ^{1}H-NMR-400 MHz spectrum of **1e** in $CDCl_3$ at 25 °C....56
Fig. S54 ^{13}C-DEPT-101 MHz spectrum of **1e** in $CDCl_3$ at 25 °C....57
Fig. S55 2D-COSY spectrum of **1e** in $CDCl_3$ at 25 °C....58
Fig. S56 2D-HSQC spectrum of **1e** in $CDCl_3$ at 25 °C....59
2^{A-G},3^{A-G}-Tetradeca-*O*-acetyl-6^A-bromo-6^A-deoxy-6^{B-G}-hexa-*O*-methyl-cyclomaltoheptaose (**3e**)....60
Fig. S57 ^{1}H-NMR-400 MHz spectrum of **3e** in $CDCl_3$ at 25 °C....60
Fig. S58 ^{13}C-DEPT-101 MHz spectrum of **3e** in $CDCl_3$ at 25 °C....61
Fig. S59 2D-COSY spectrum of **3e** in $CDCl_3$ at 25 °C....62
Fig. S60 2D-HSQC spectrum of **3e** in $CDCl_3$ at 25 °C....63
2^{A-G},3^{A-G}-Tetradeca-*O*-acetyl-6^A-deoxy-6^A-iodo-6^{B-G}-hexa-*O*-methyl-cyclomaltoheptaose (**4e**)....64
Fig. S61 ^{1}H-NMR-400 MHz spectrum of **4e** in $CDCl_3$ at 25 °C....64
Fig. S62 ^{13}C-DEPT-101 MHz spectrum of **4e** in $CDCl_3$ at 25 °C....65
Fig. S63 2D-COSY spectrum of **4e** in $CDCl_3$ at 25 °C....66
Fig. S64 2D-HSQC spectrum of **4e** in $CDCl_3$ at 25 °C....67

6^{A}-Amino-6^{A}-deoxy-6$^{B\text{-}G}$-hexa-*O*-methyl-cyclomaltoheptaose (**1b**)

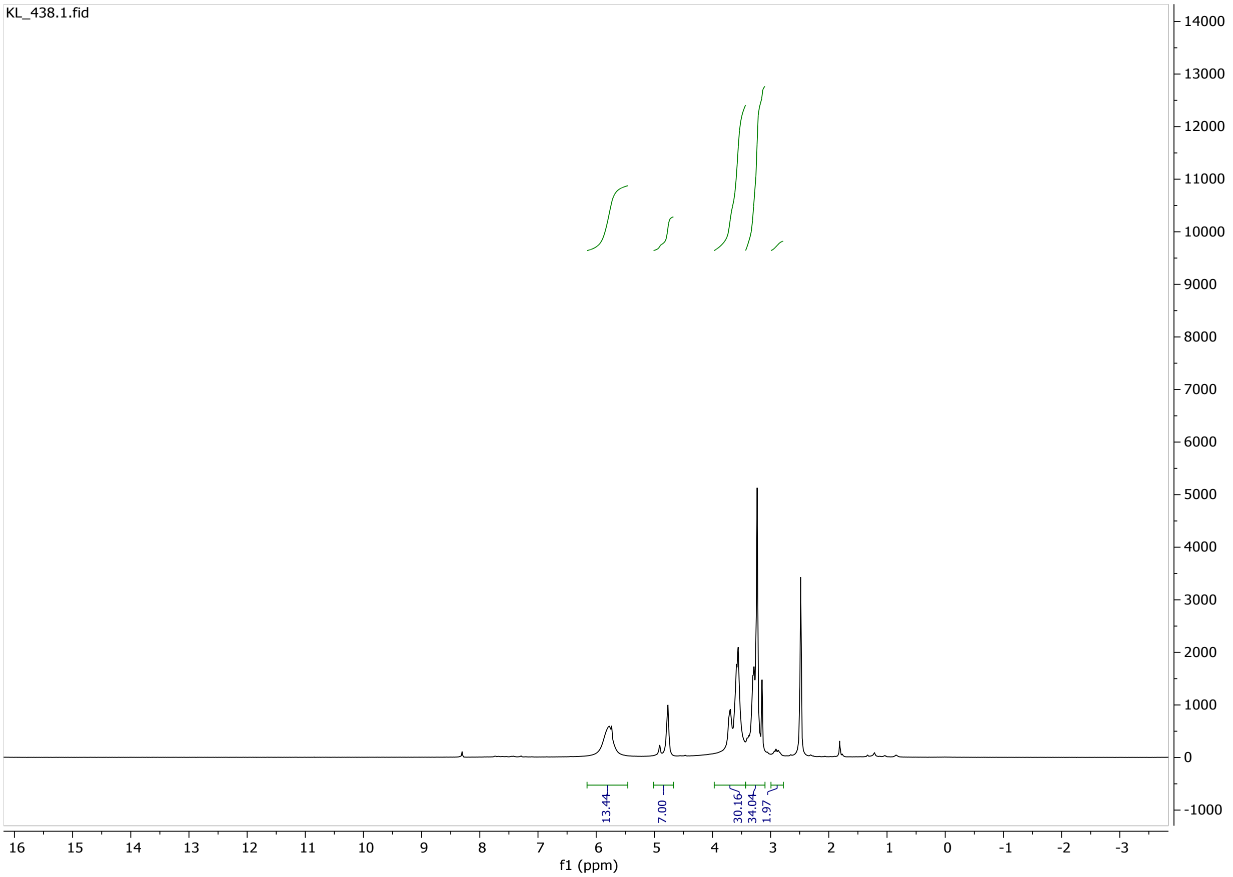

Fig. S1 ^{1}H-NMR-400 MHz spectrum of **1b** in DMSO-d_6 at 25 °C

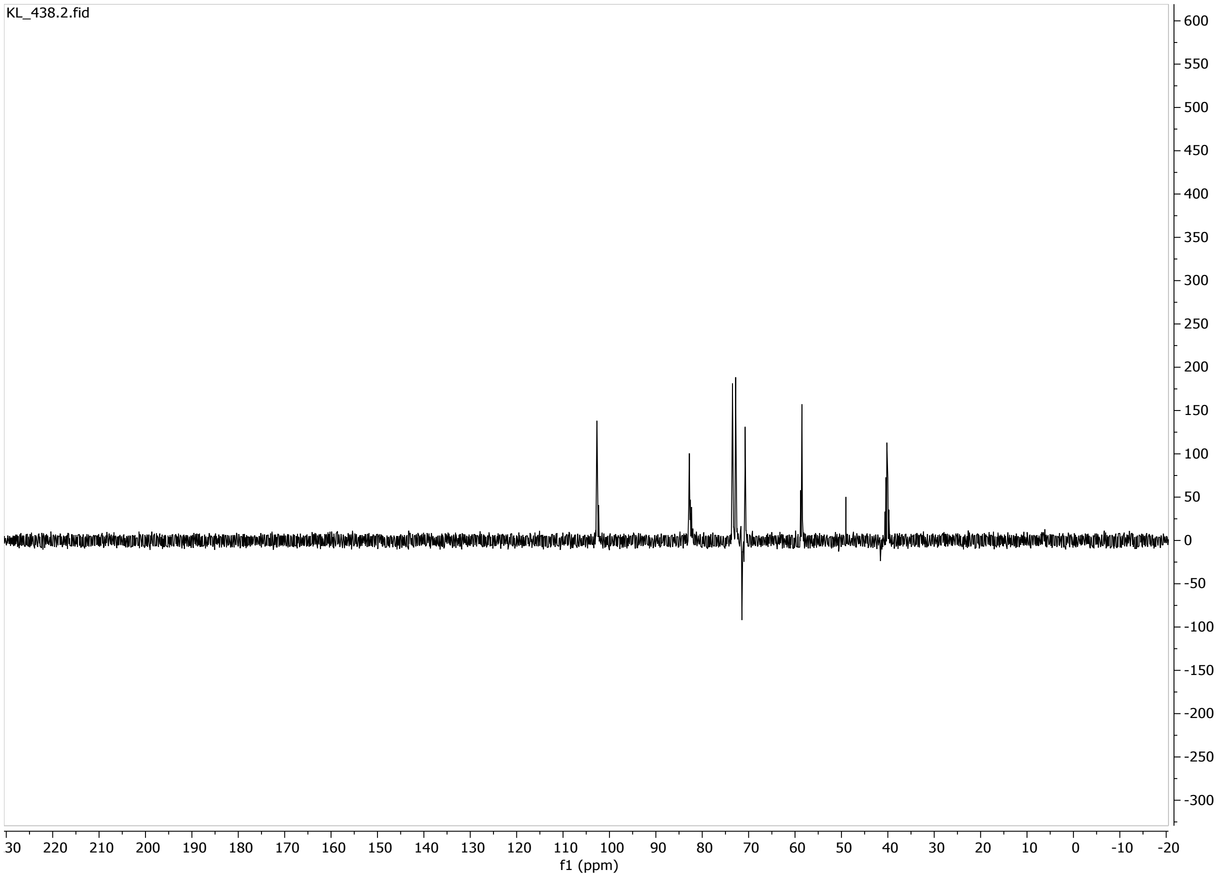

Fig. S2 ^{13}C-DEPT-101 MHz spectrum of **1b** in DMSO-d_6 at 25 °C

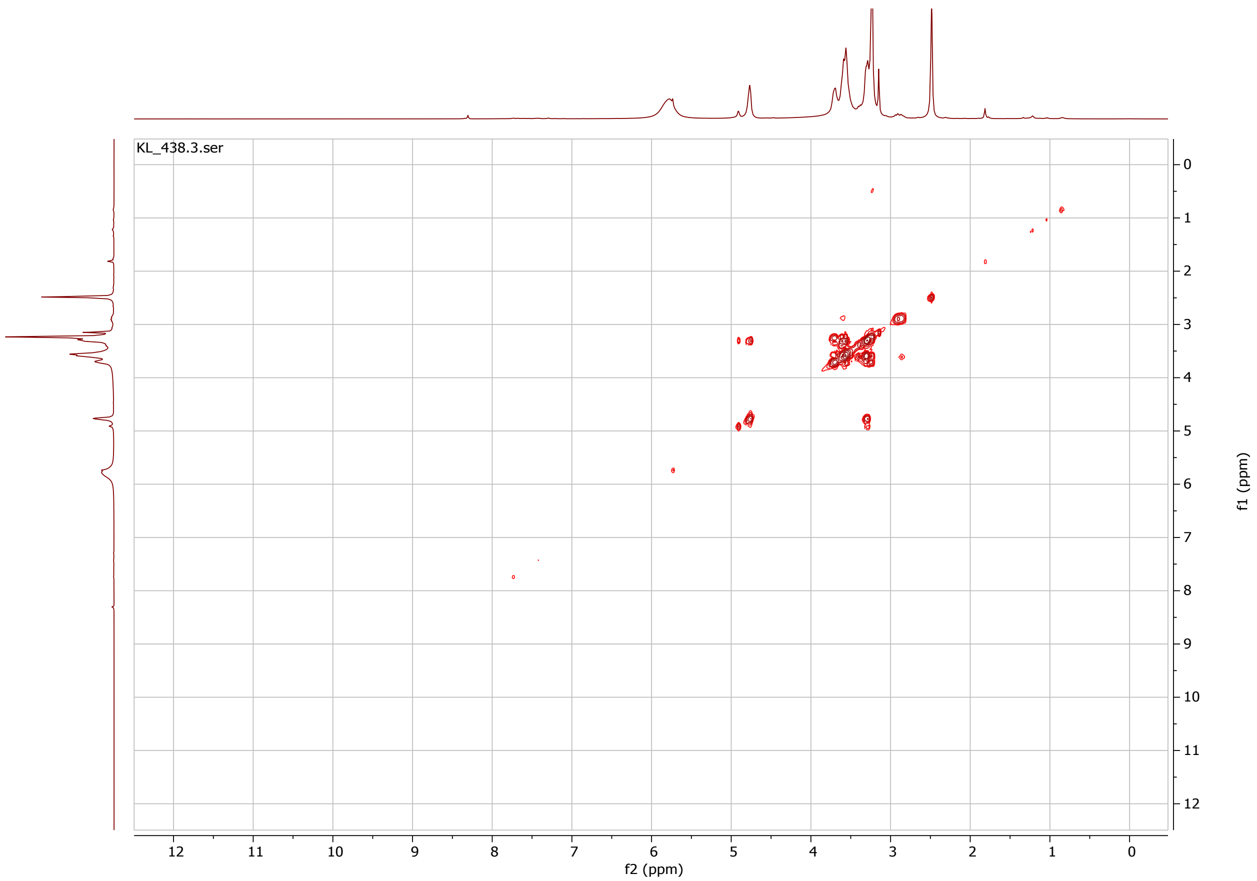

Fig. S3 2D-COSY spectrum of **1b** in DMSO-d_6 at 25 °C

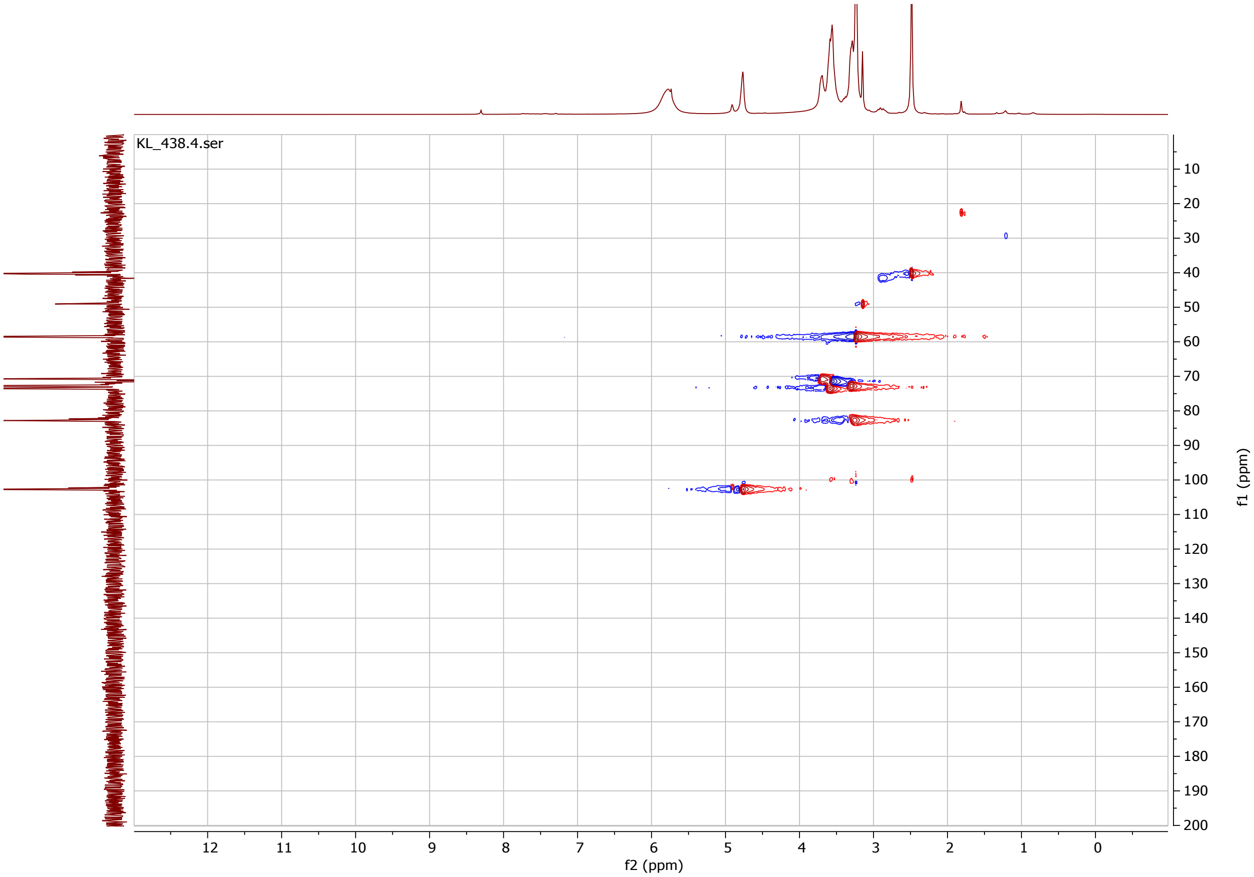

Fig. S4 2D-HSQC spectrum of **1b** in DMSO-d_6 at 25 °C

6^A-Chloro-6^A-deoxy-2^{A-G},3^{A-G}-tetradeca-*O*-methyl-cyclomaltoheptaose (**2c**)

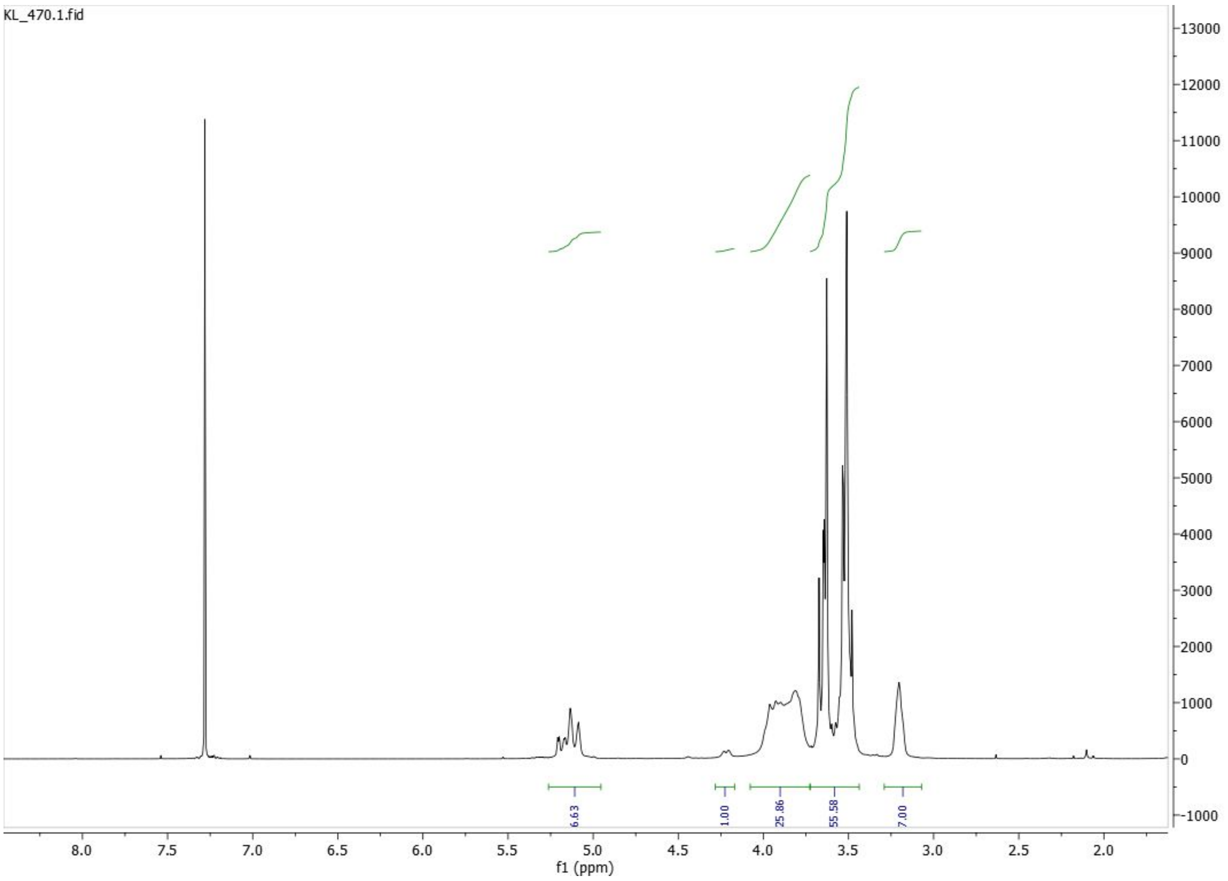

Fig. S5 ^{1}H-NMR-400 MHz spectrum of **2c** in $CDCl_3$ at 25 °C

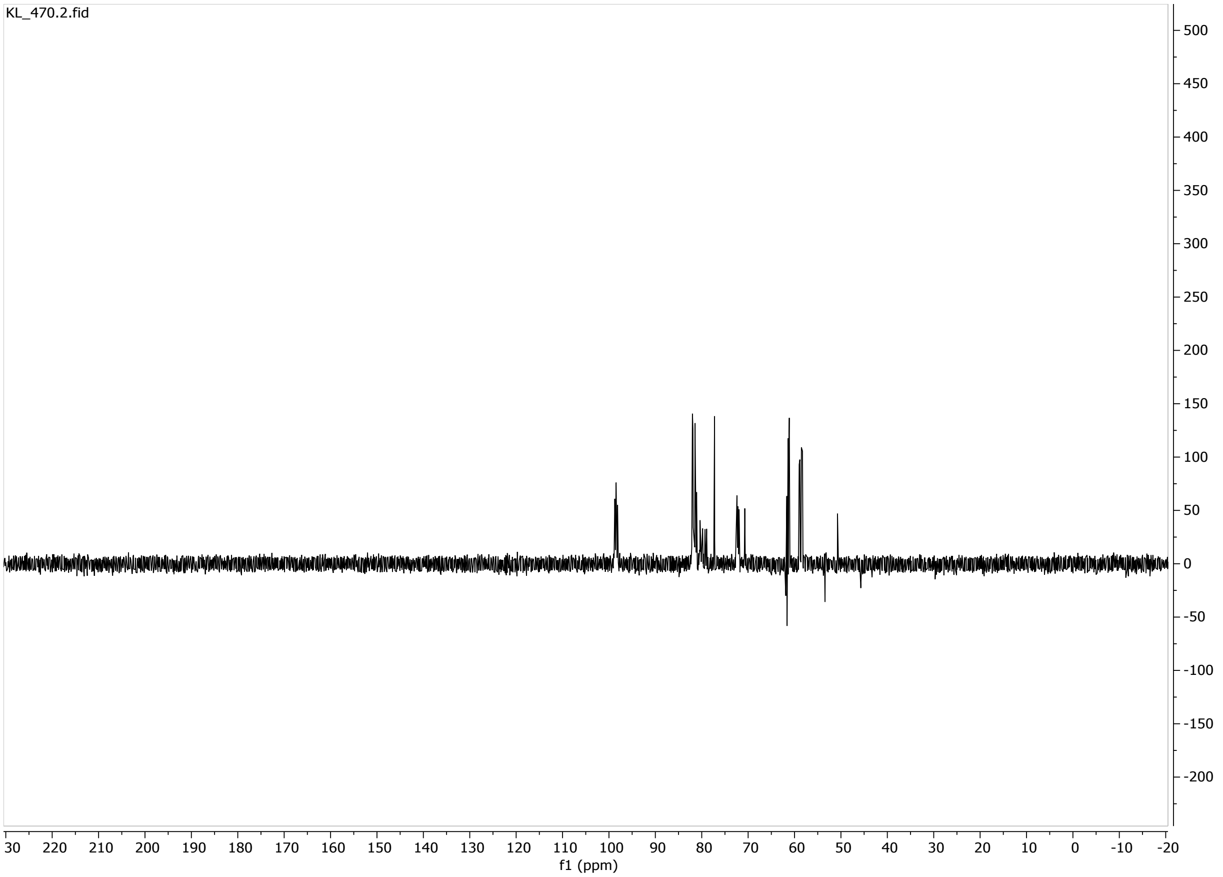

Fig. S6 ^{13}C-DEPT-101 MHz spectrum of **2c** in $CDCl_3$ at 25 °C

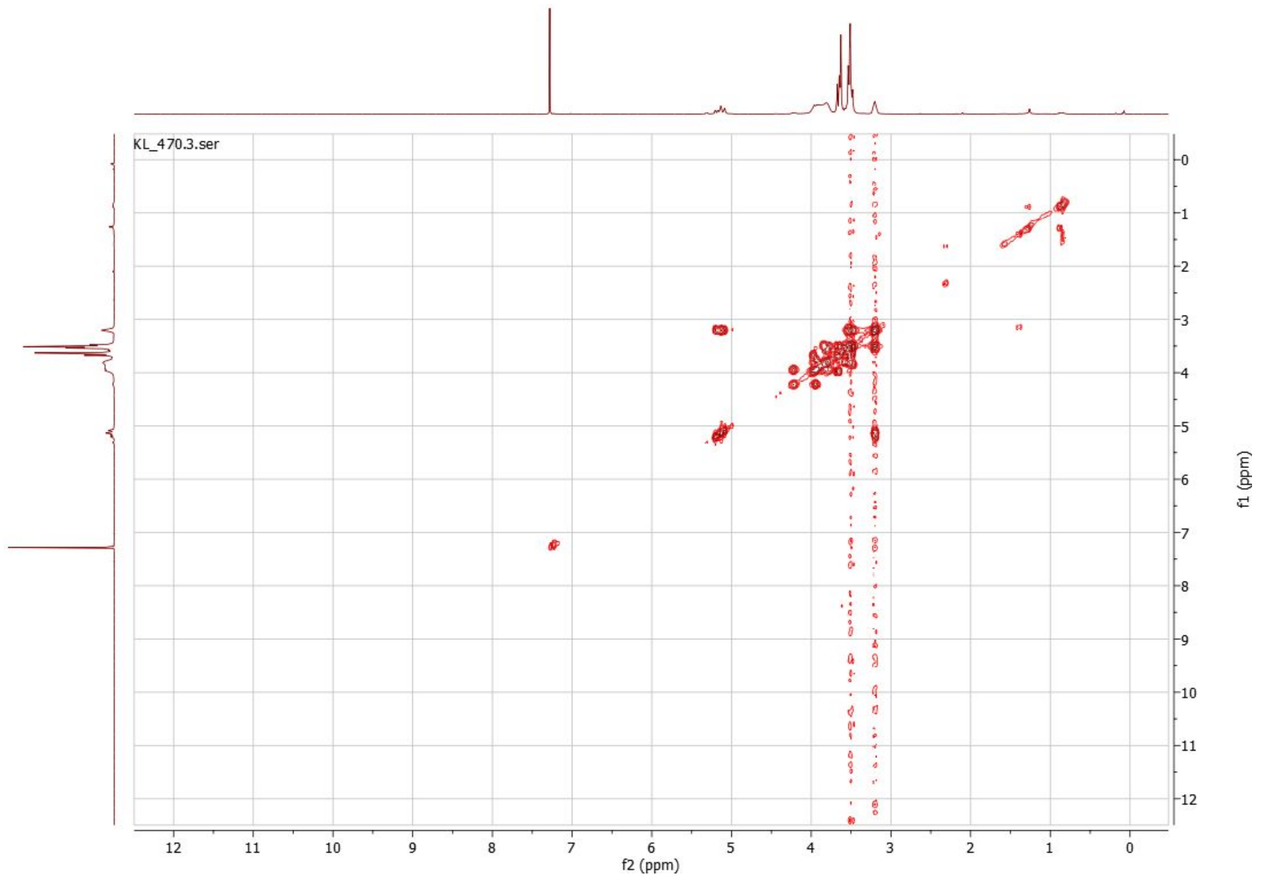

Fig. S7 2D-COSY spectrum of **2c** in $CDCl_3$ at 25 °C

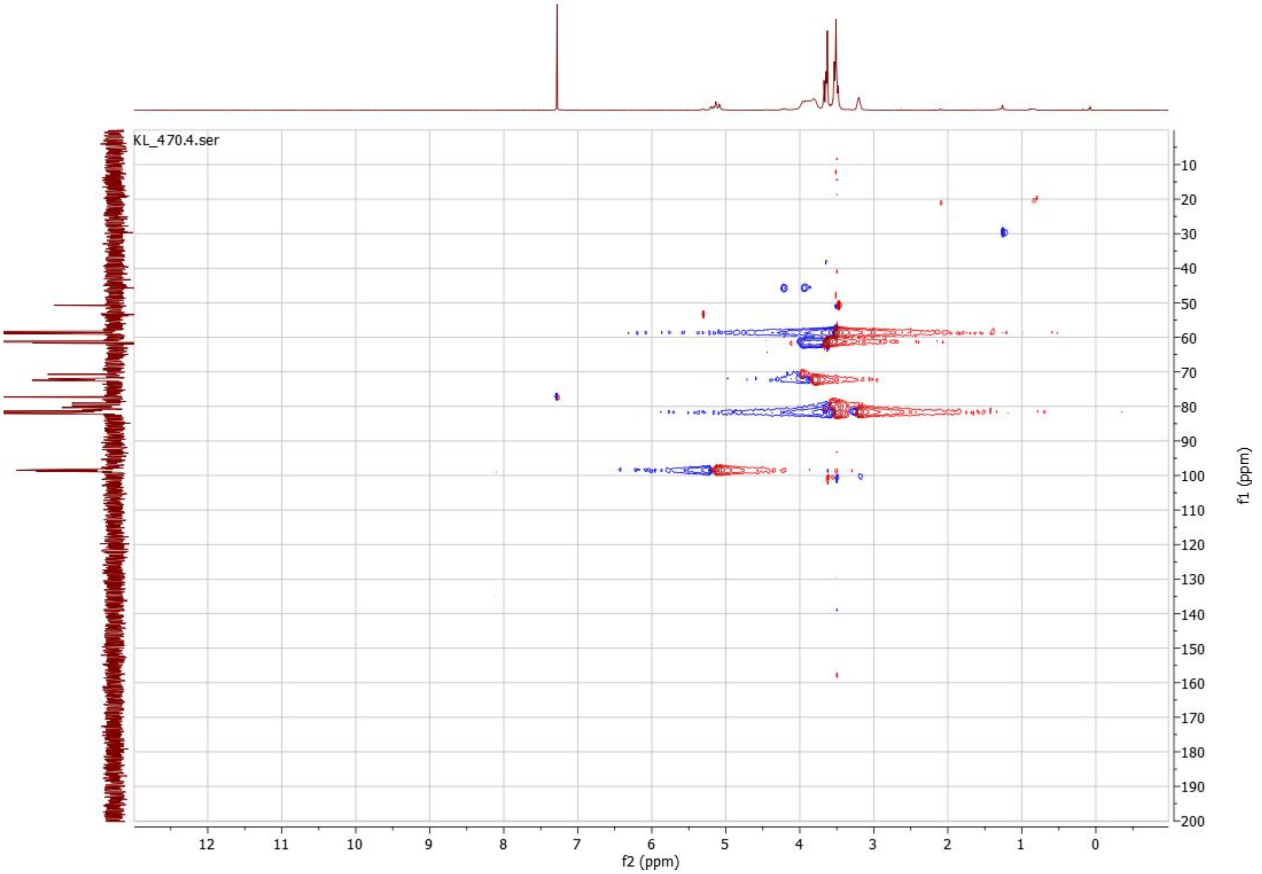

Fig. S8 2D-HSQC spectrum of **2c** in $CDCl_3$ at 25 °C

6^A-Bromo-6^A-deoxy-2^A-G,3^A-G-tetradeca-*O*-methyl-cyclomaltoheptaose (**3c**)

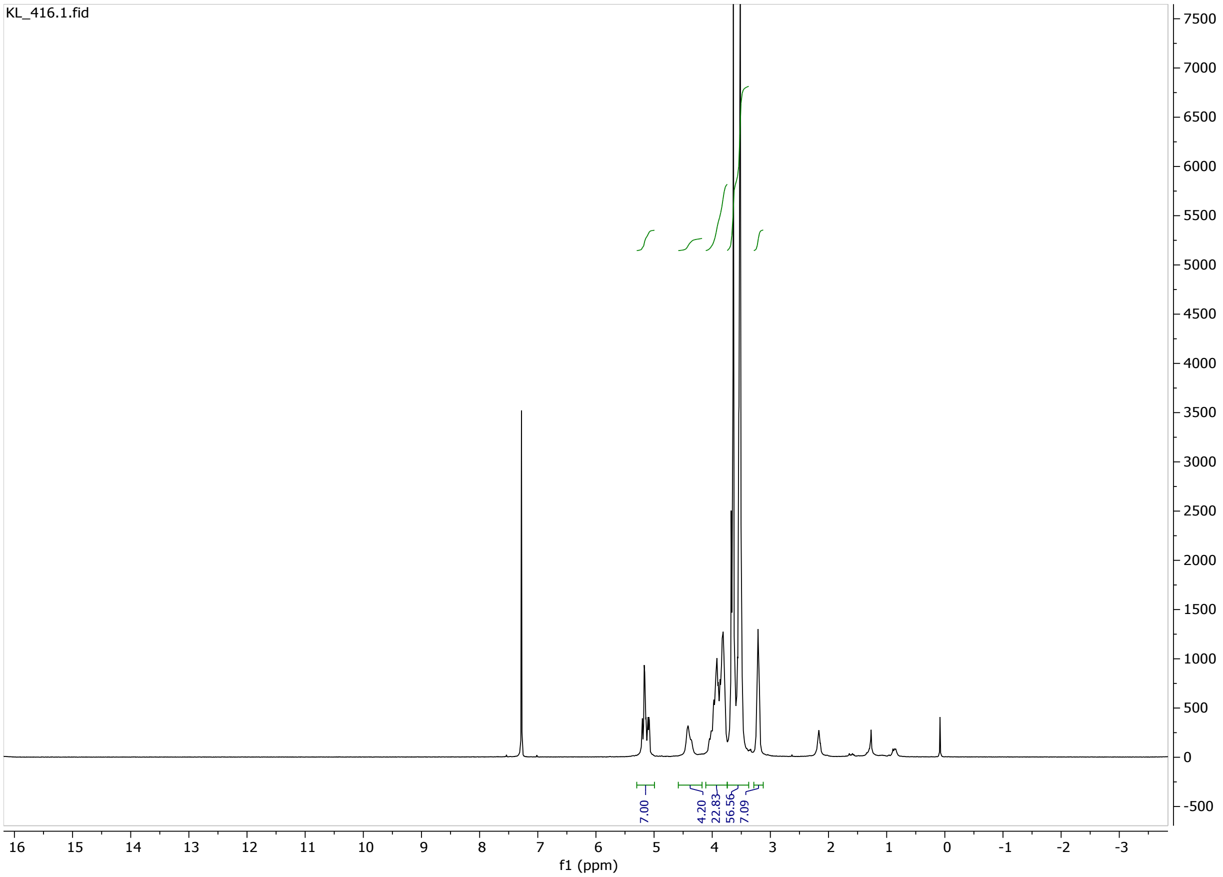

Fig. S9 ^{1}H-NMR-400 MHz spectrum of **3c** in $CDCl_3$ at 25 °C

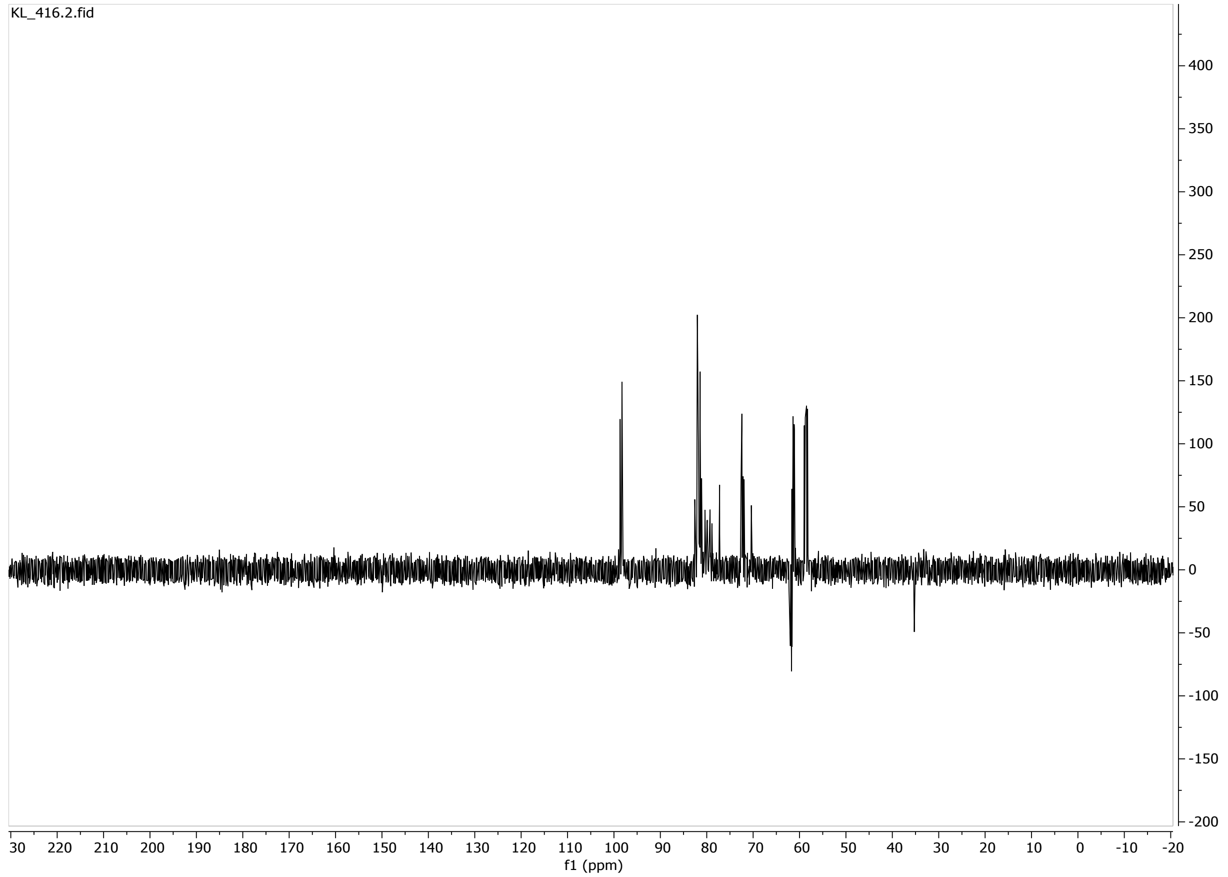

Fig. S10 ^{13}C-DEPT-101 MHz spectrum of **3c** in $CDCl_3$ at 25 °C

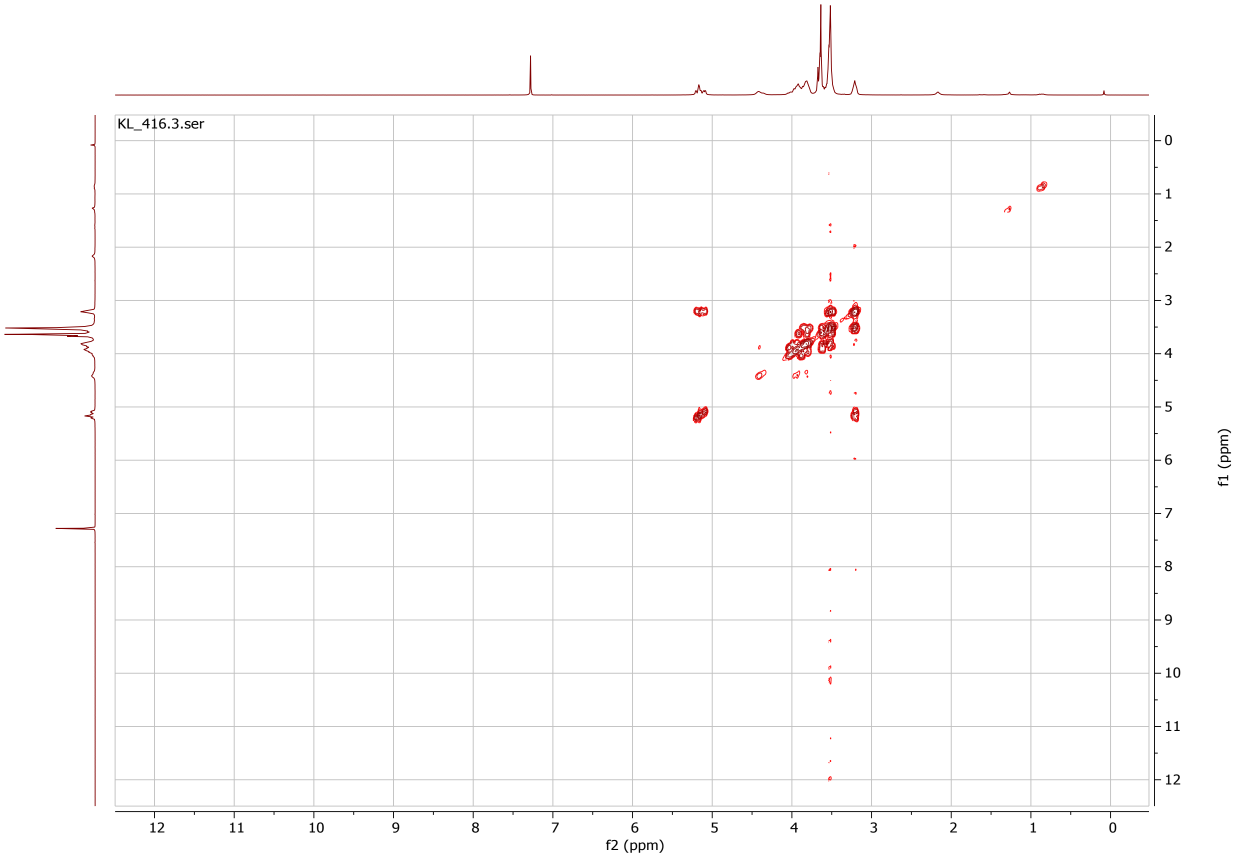

Fig. S11 2D-COSY spectrum of **3c** in $CDCl_3$ at 25 °C

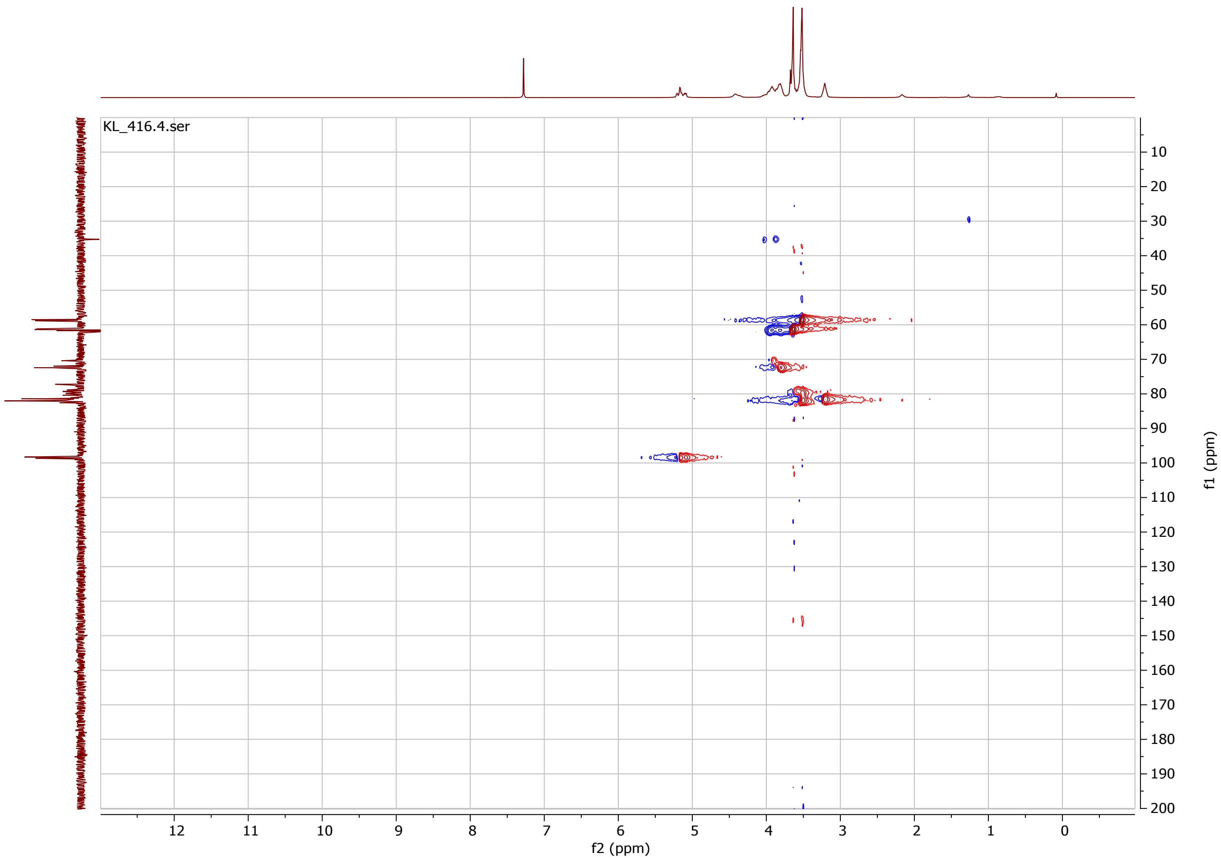

Fig. S12 2D-HSQC spectrum of **3c** in $CDCl_3$ at 25 °C

6^{A}-Deoxy-6^{A}-iodo-2^{A-G},3^{A-G}-tetradeca-*O*-methyl-cyclomaltoheptaose (**4c**)

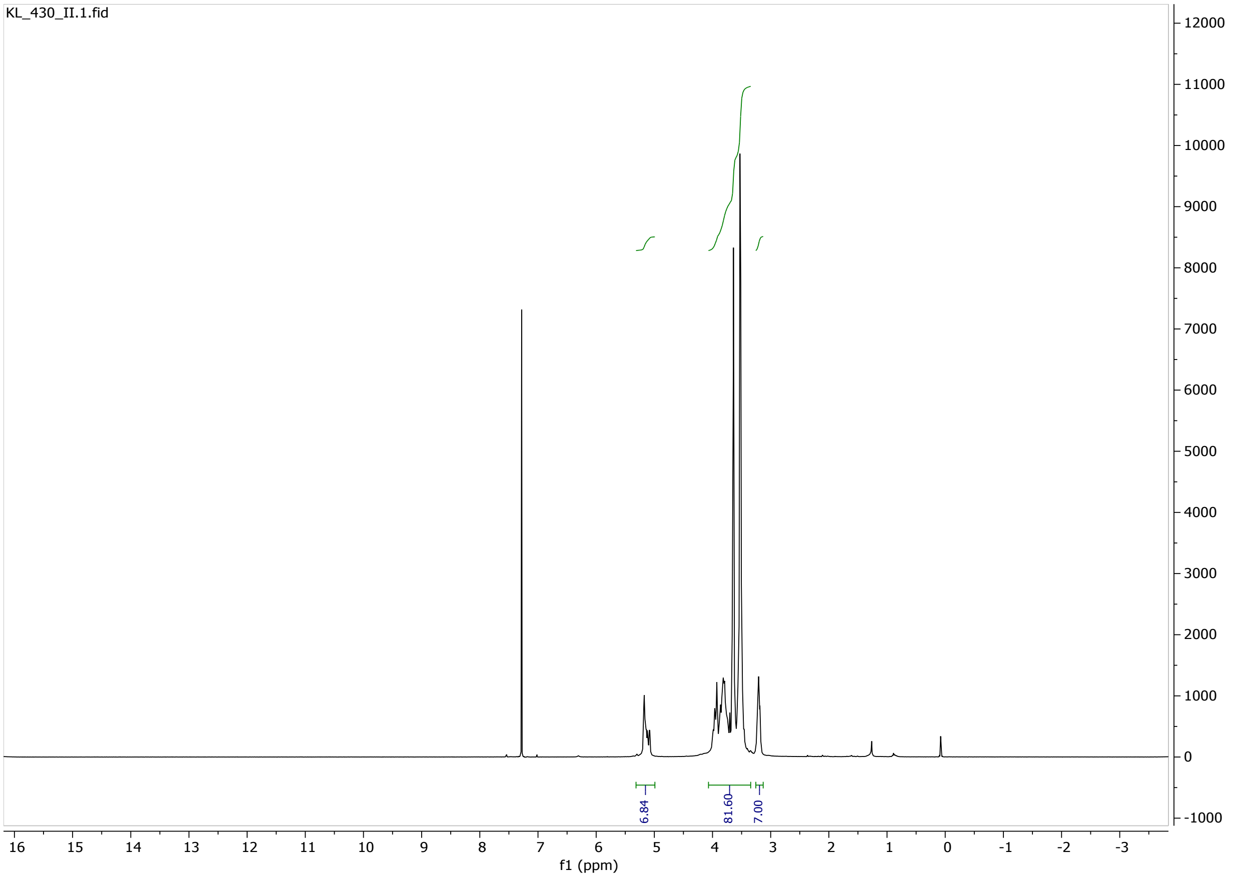

Fig. S13 ^{1}H-NMR-400 MHz spectrum of **4c** in $CDCl_3$ at 25 °C

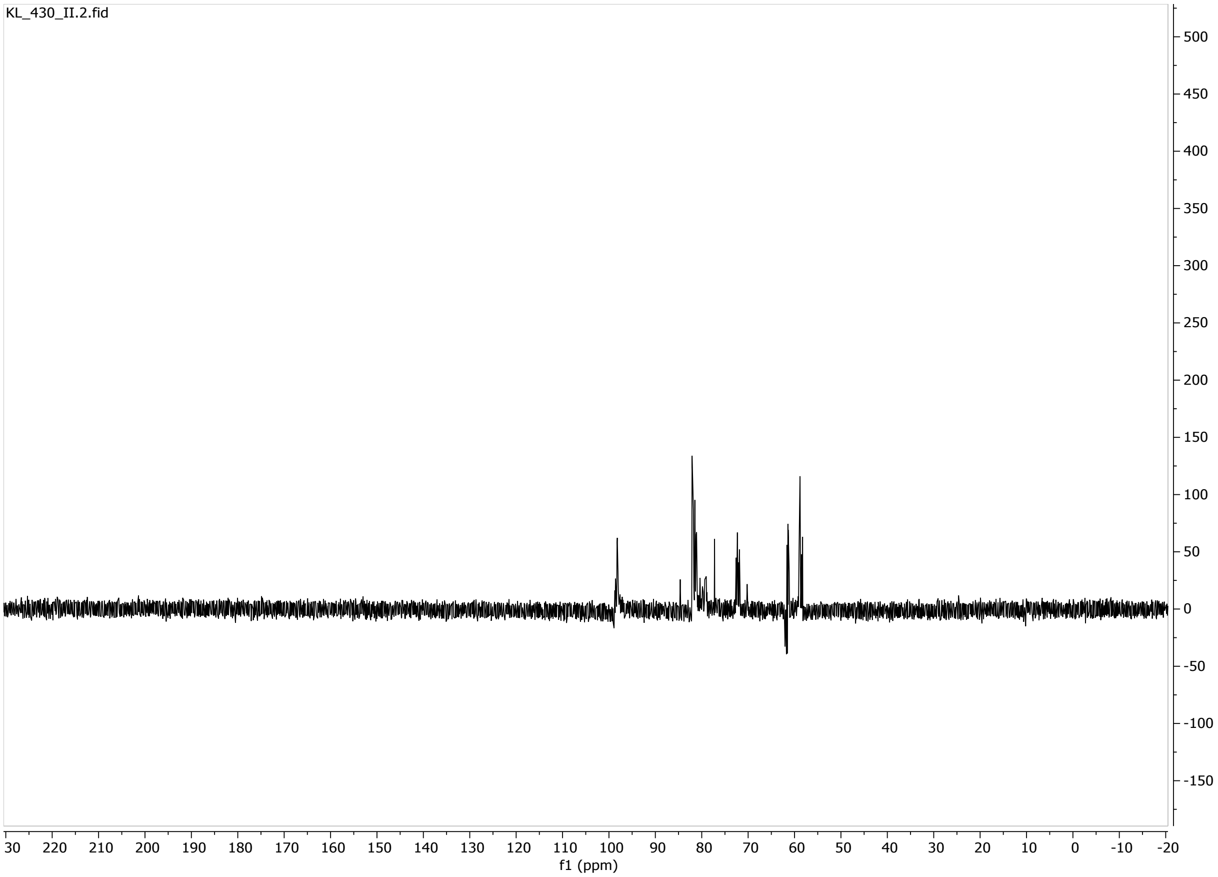

Fig. S14 ^{13}C-DEPT-101 MHz spectrum of **4c** in $CDCl_3$ at 25 °C

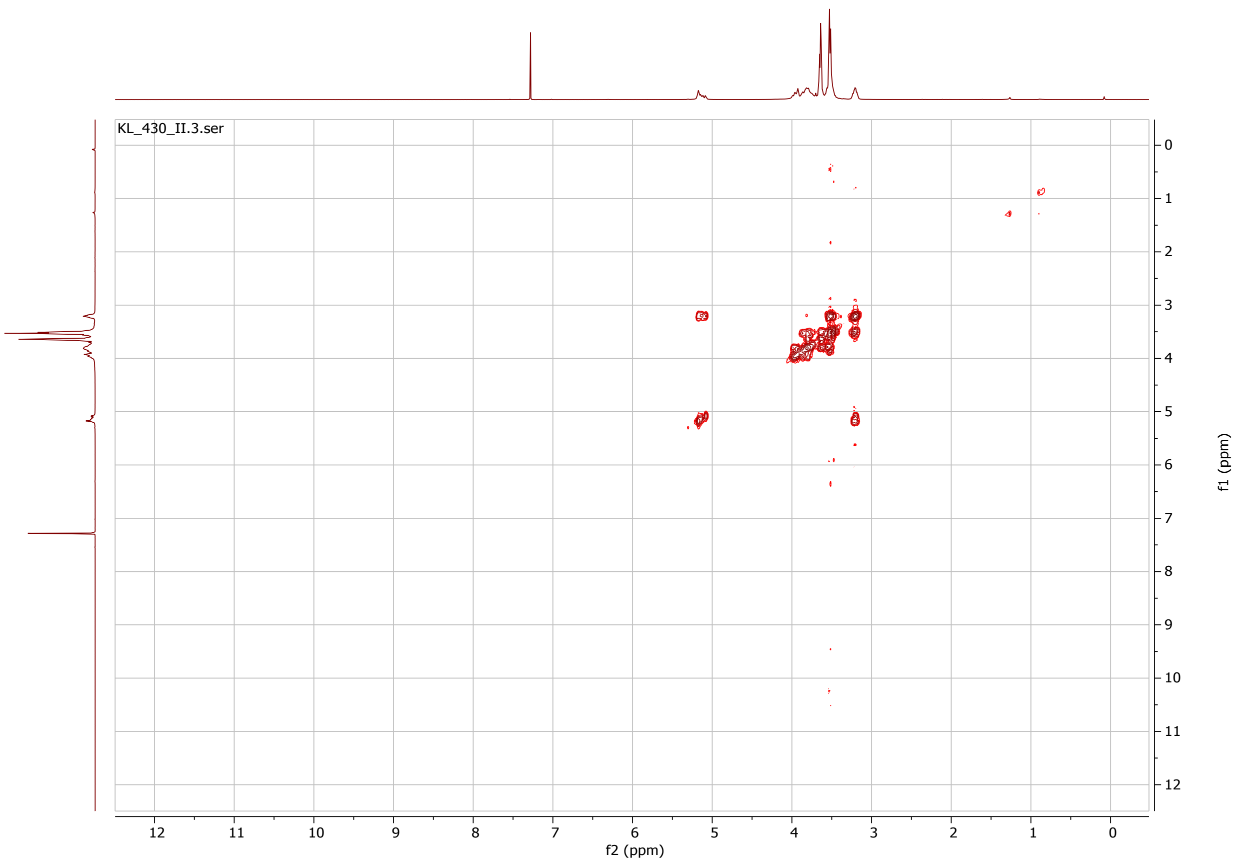

Fig. S15 2D-COSY spectrum of **4c** in $CDCl_3$ at 25 °C

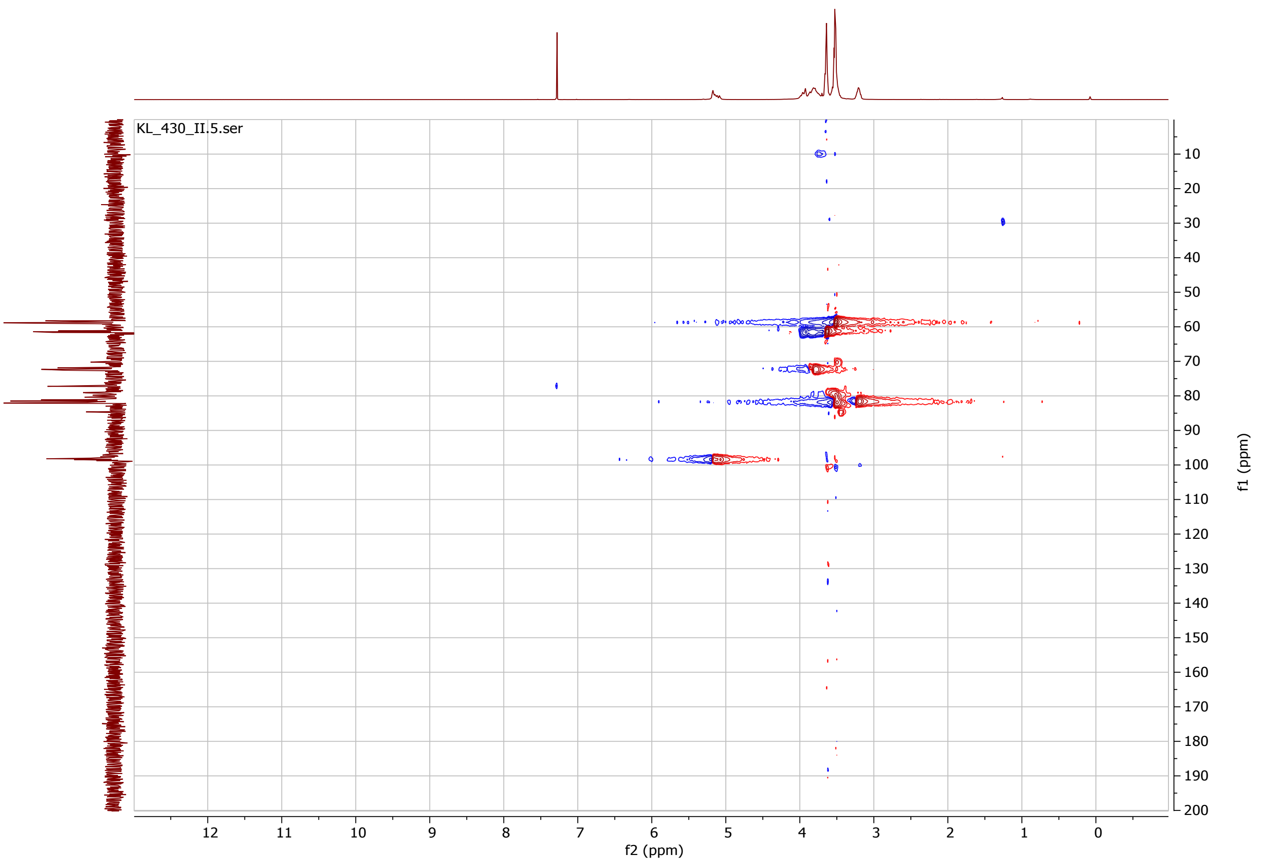

Fig. S16 2D-HSQC spectrum of **4c** in $CDCl_3$ at 25 °C

6^{A}-Chloro-6^{A}-deoxy-2^{A-G},3^{A-G},6^{B-G}-icosa-*O*-methyl-cyclomaltoheptaose (**2d**)

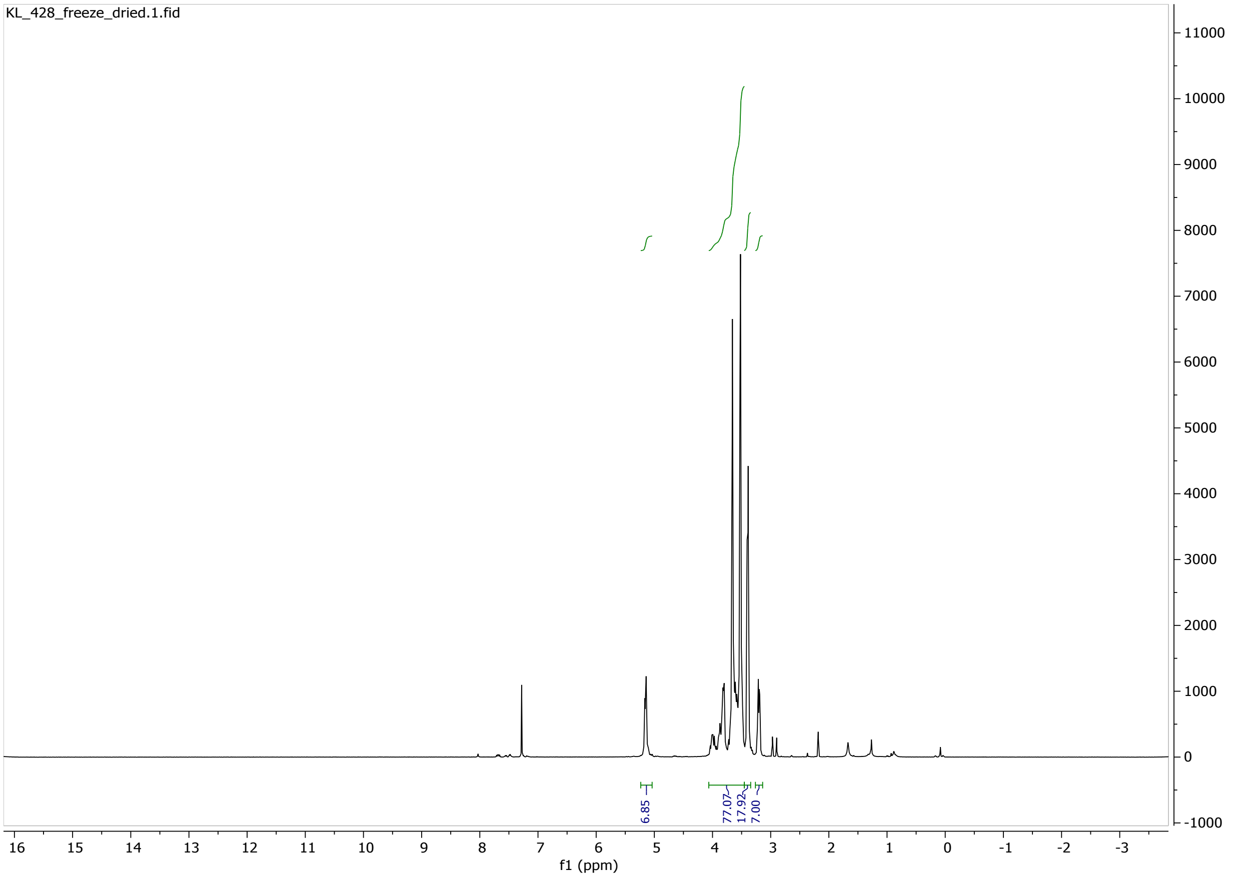

Fig. S17 ^{1}H-NMR-400 MHz spectrum of **2d** in $CDCl_3$ at 25 °C

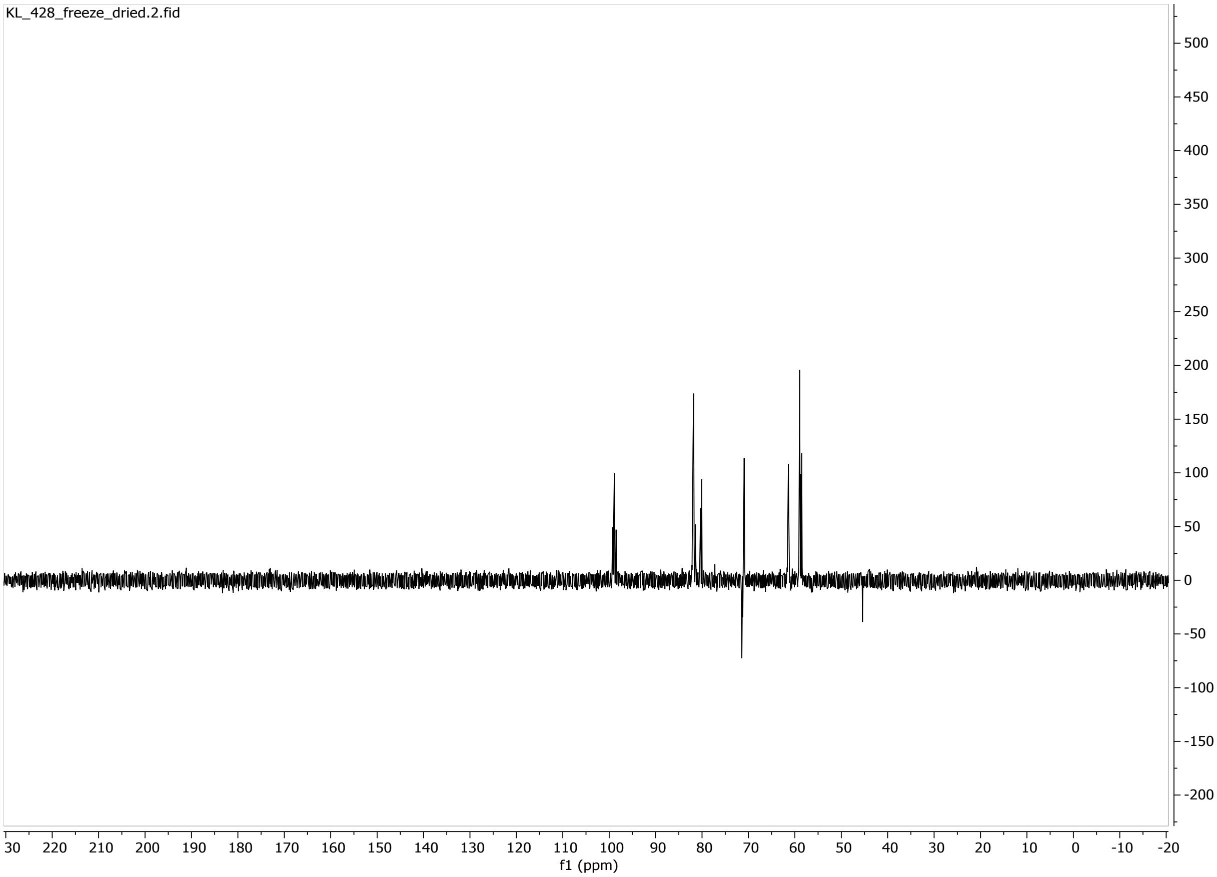

Fig. S18 ^{13}C-DEPT-101 MHz spectrum of **2d** in $CDCl_3$ at 25 °C

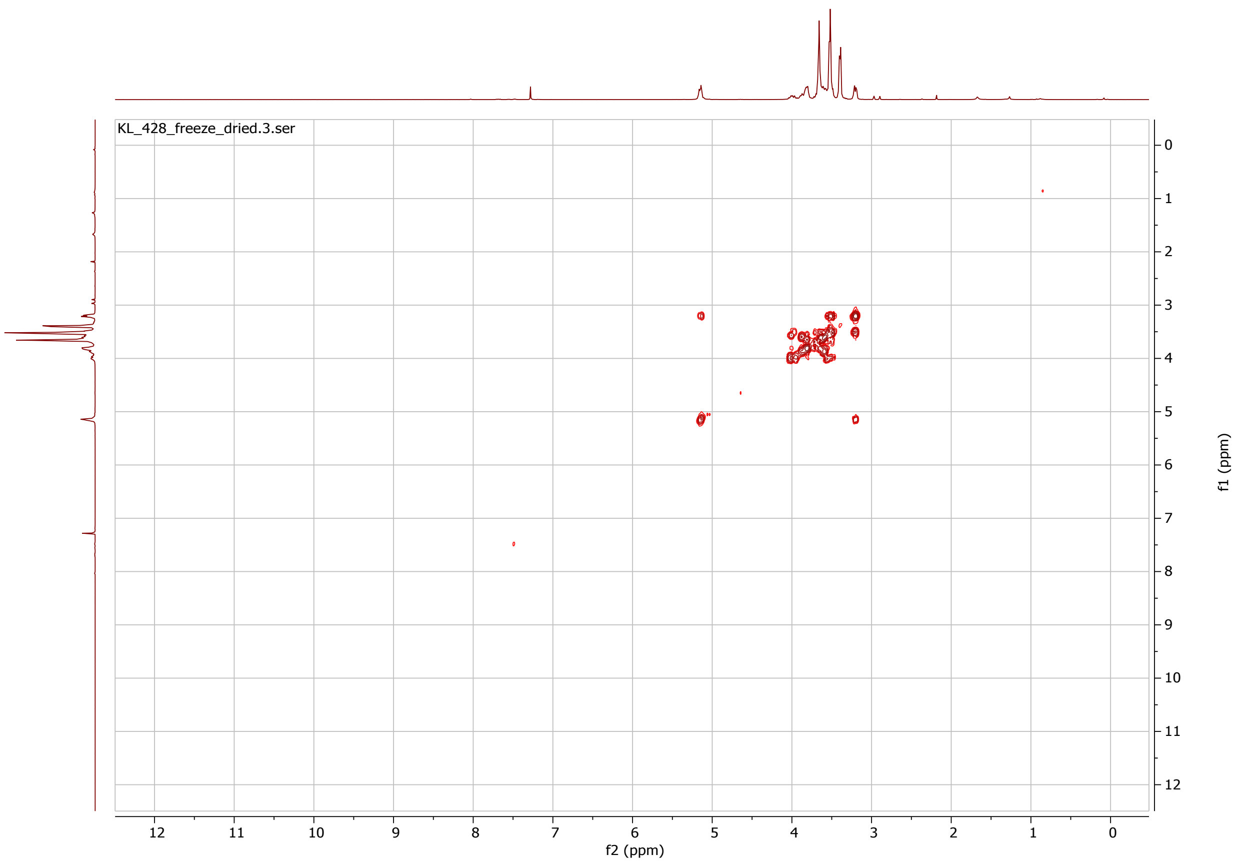

Fig. S19 2D-COSY spectrum of **2d** in $CDCl_3$ at 25 °C

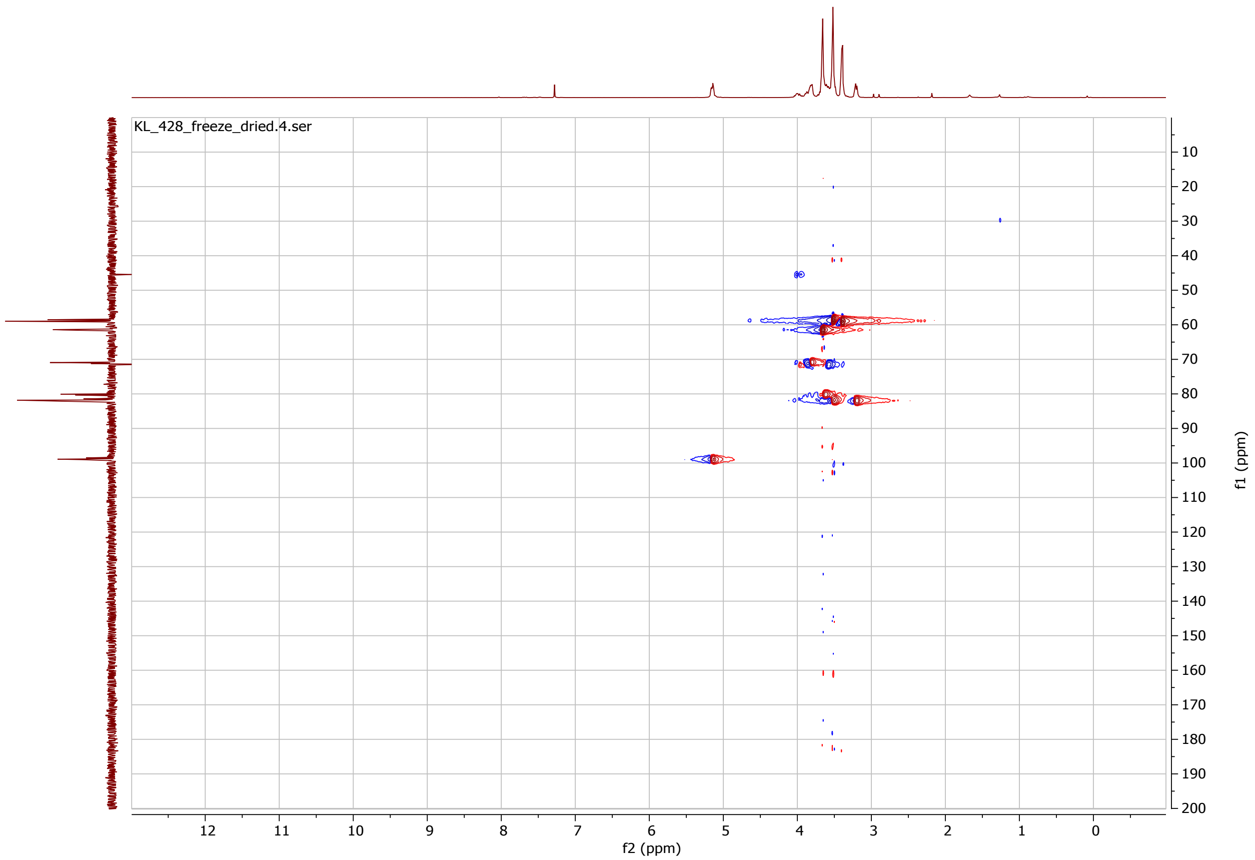

Fig. S20 2D-HSQC spectrum of **2d** in $CDCl_3$ at 25 °C

6^A-Bromo-6^A-deoxy-2^{A-G},3^{A-G},6^{B-G}-icosa-*O*-methyl-cyclomaltoheptaose (**3d**)

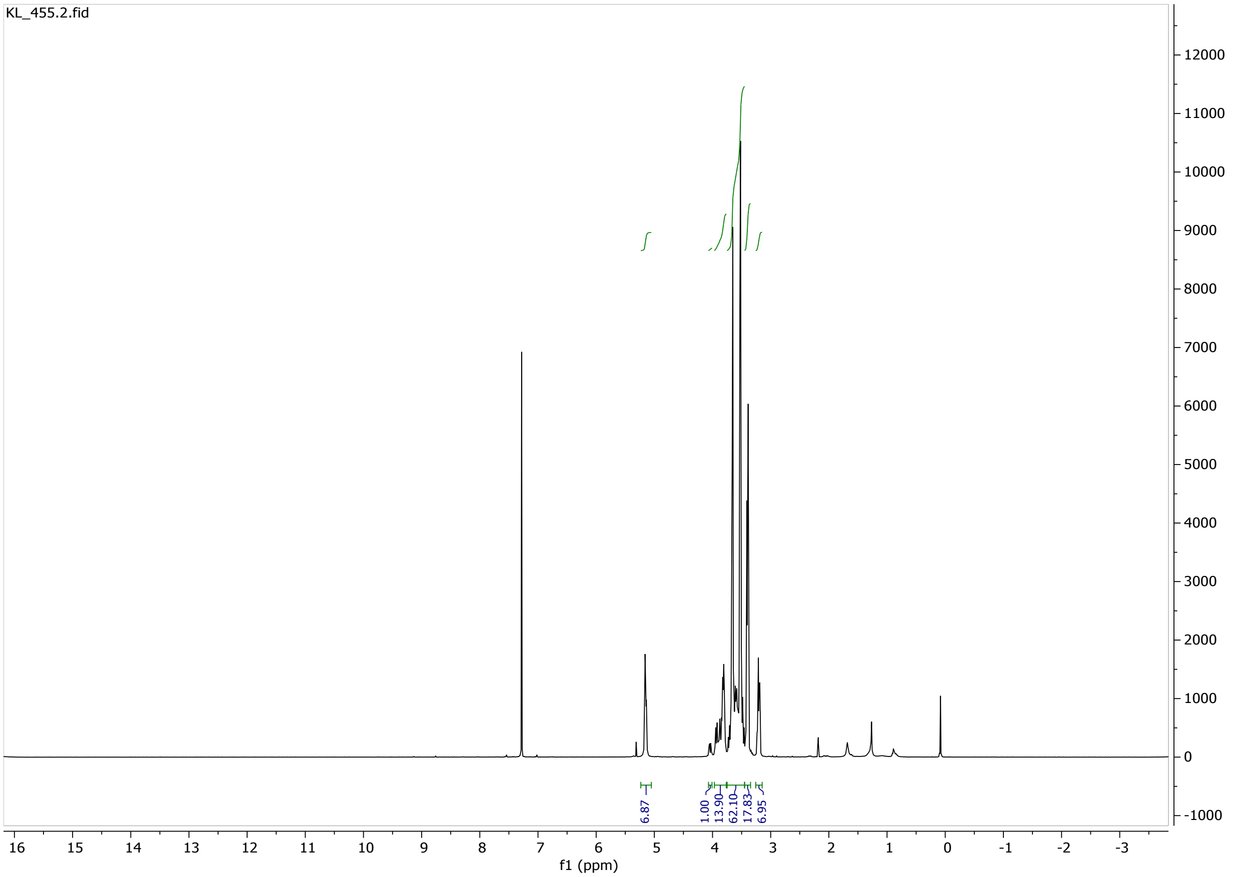

Fig. S21 ^{1}H-NMR-400 MHz spectrum of **3d** in $CDCl_3$ at 25 °C

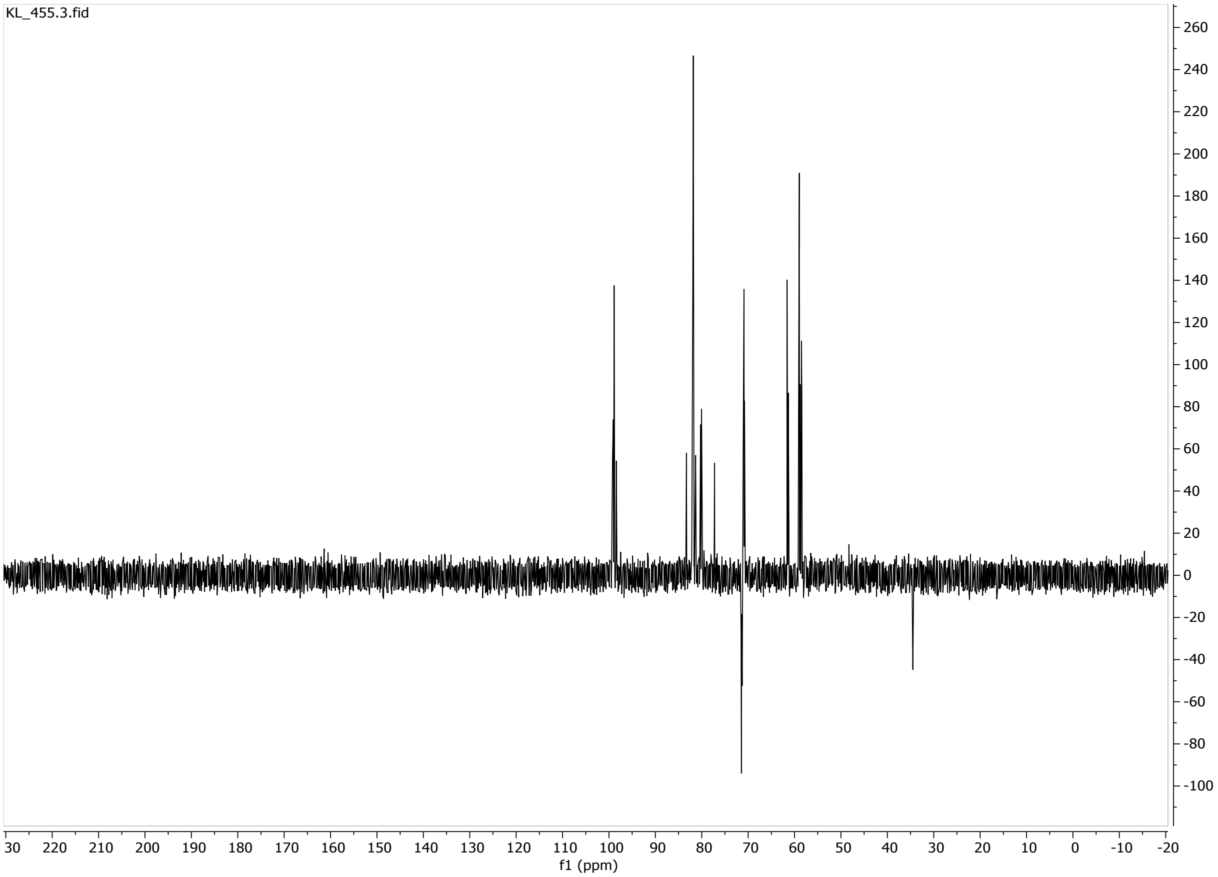

Fig. S22 ^{13}C-DEPT-101 MHz spectrum of **3d** in $CDCl_3$ at 25 °C

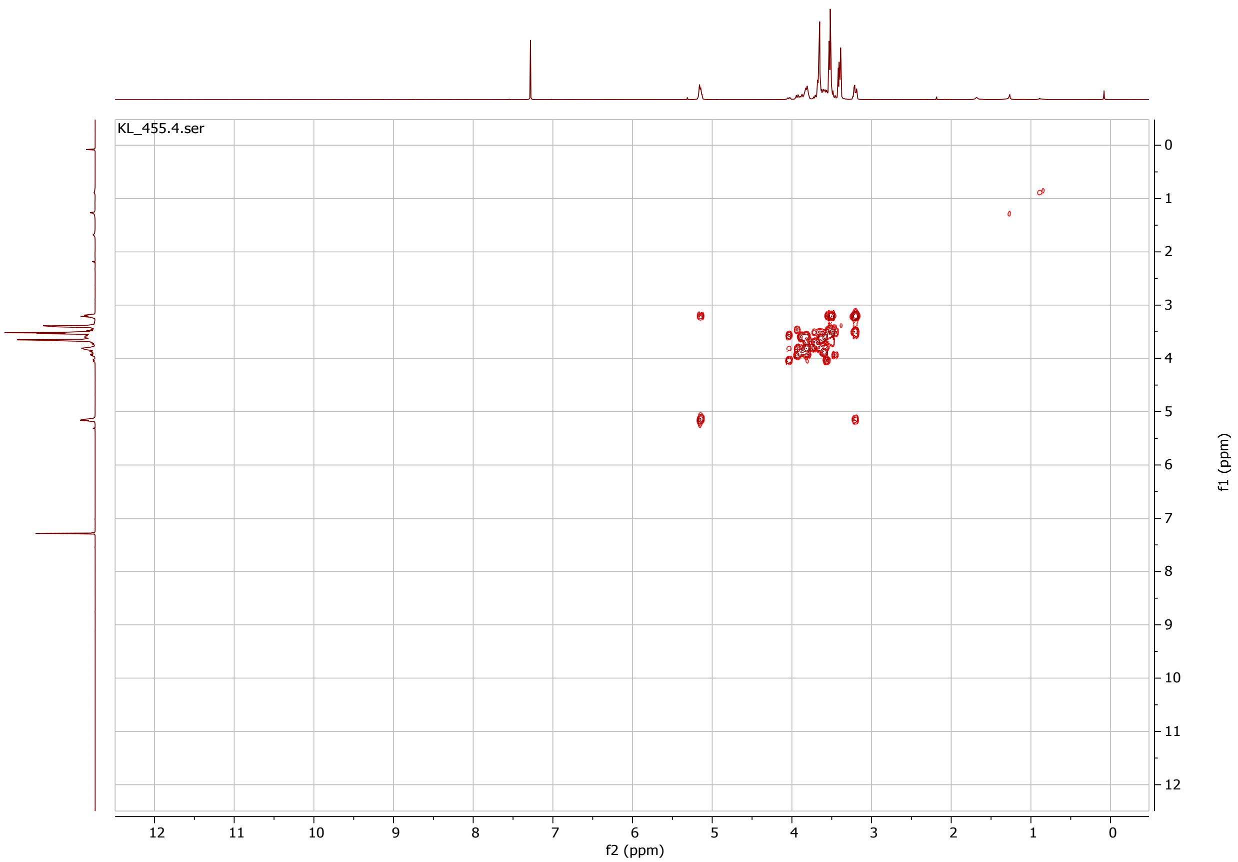

Fig. S23 2D-COSY spectrum of **3d** in $CDCl_3$ at 25 °C

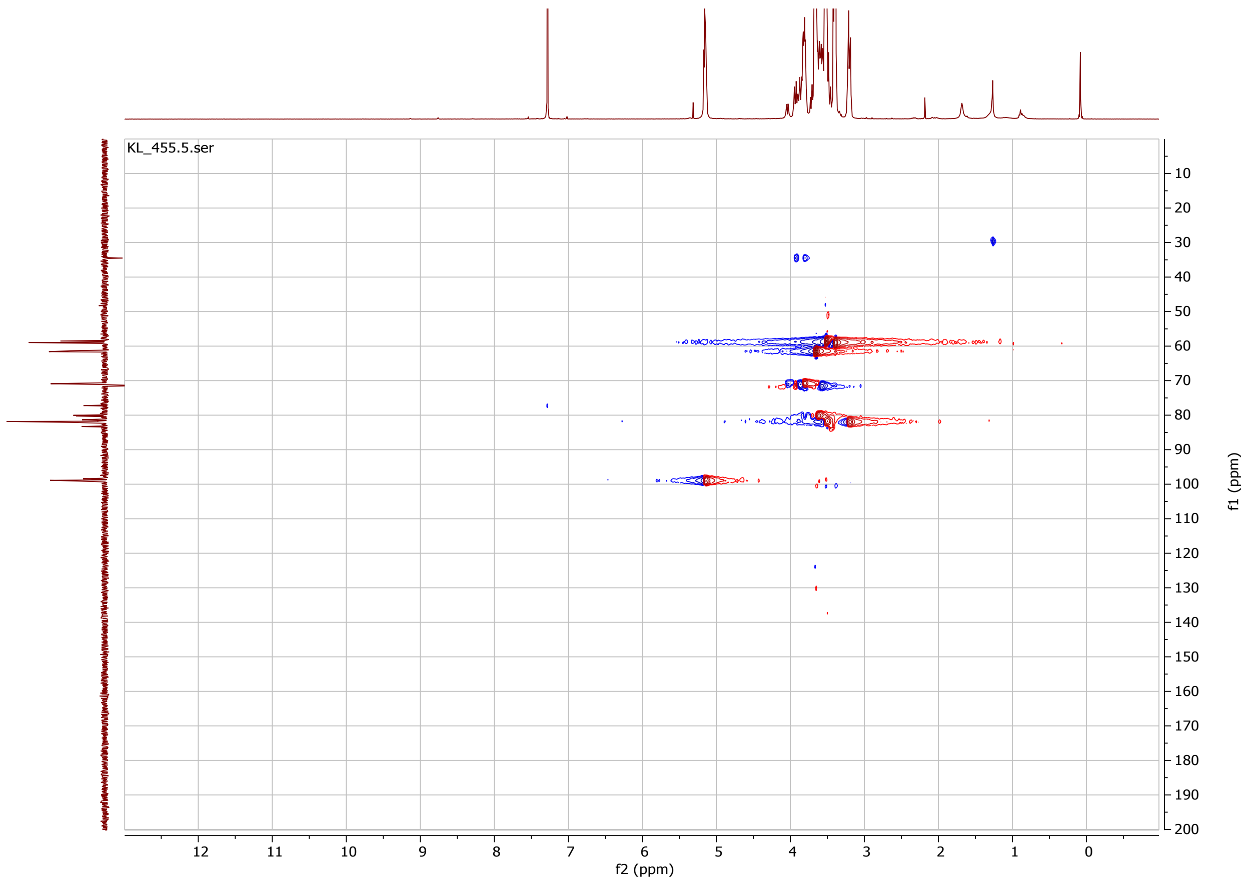

Fig. S24 2D-HSQC spectrum of **3d** in $CDCl_3$ at 25 °C

6^A-Deoxy-6^A-iodo-2^{A-G},3^{A-G},6^{B-G}-icosa-*O*-methyl-cyclomaltoheptaose (**4d**)

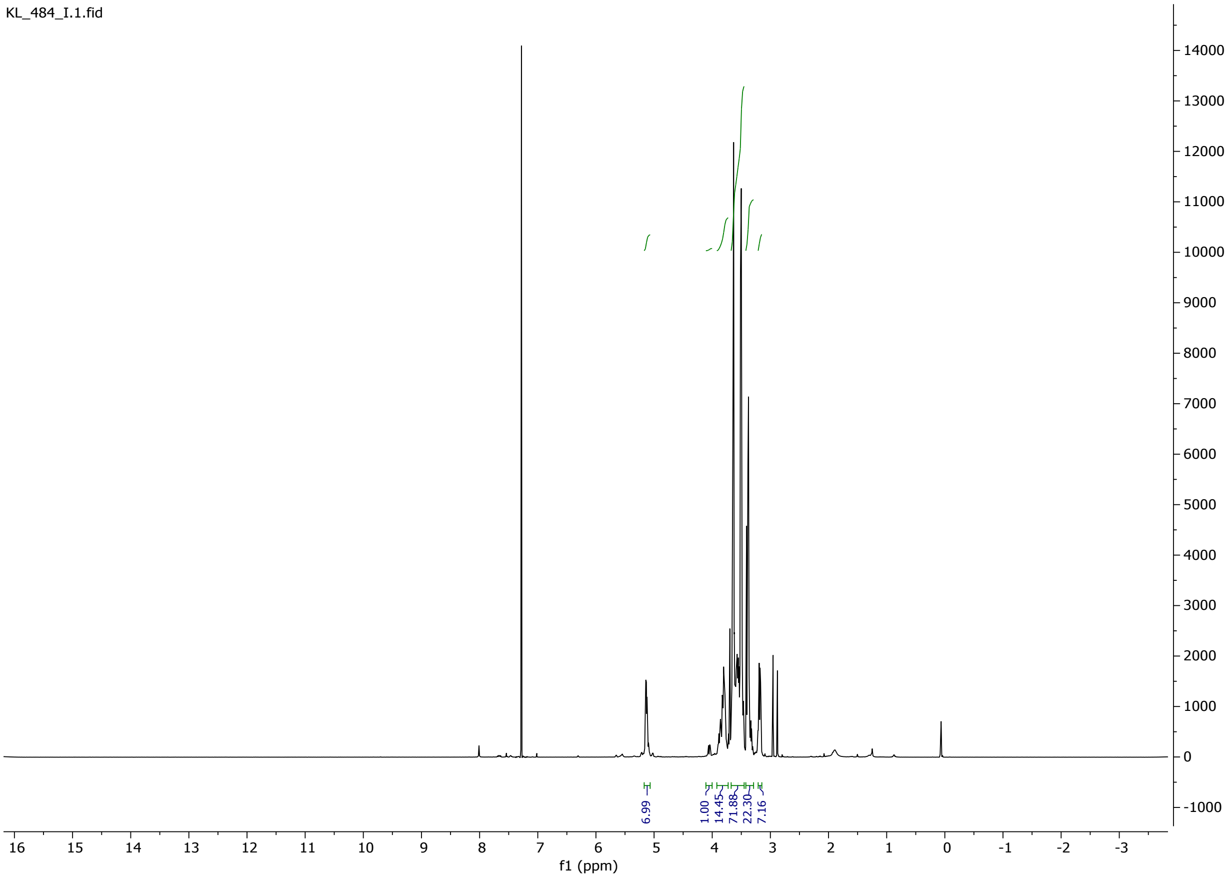

Fig. S25 ^{1}H-NMR-400 MHz spectrum of **4d** in $CDCl_3$ at 25 °C

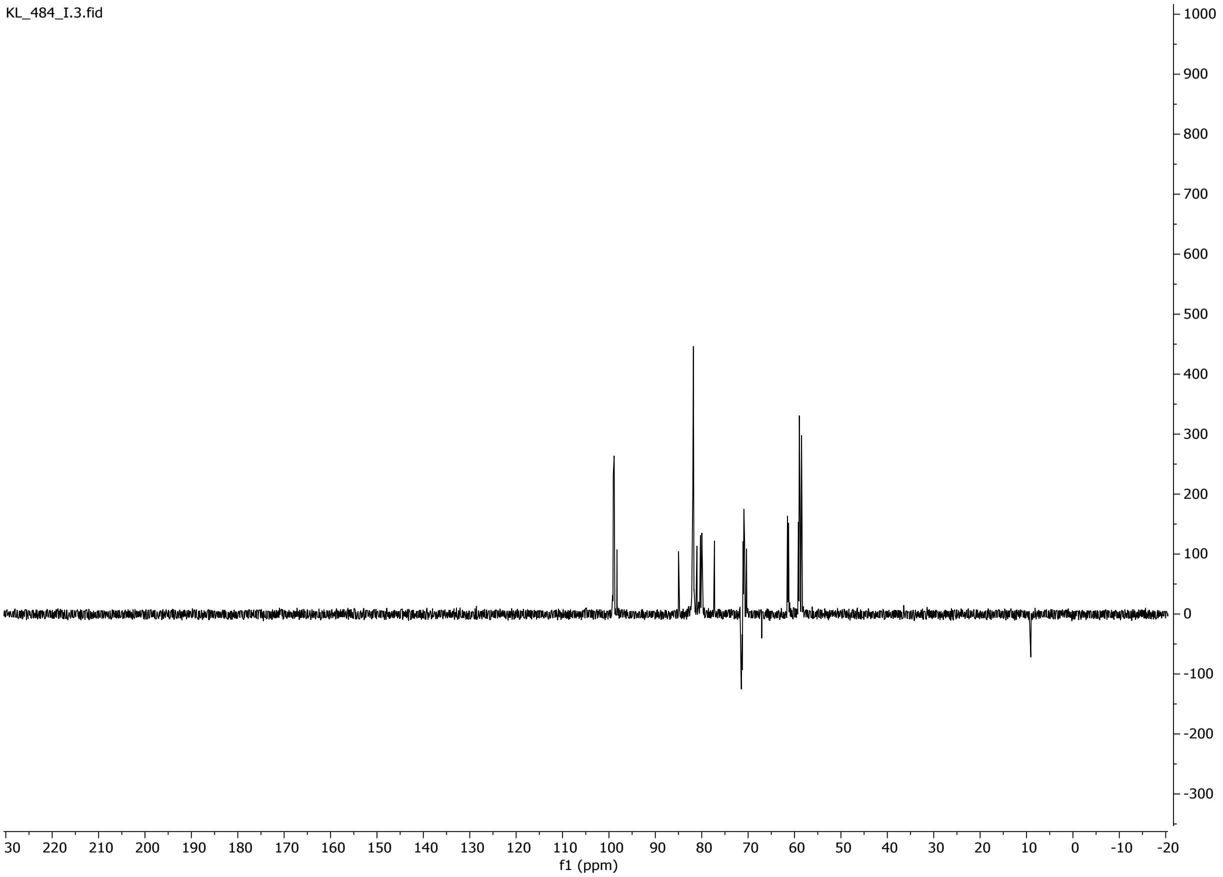

Fig. S26 ^{13}C-DEPT-101 MHz spectrum of **4d** in $CDCl_3$ at 25 °C

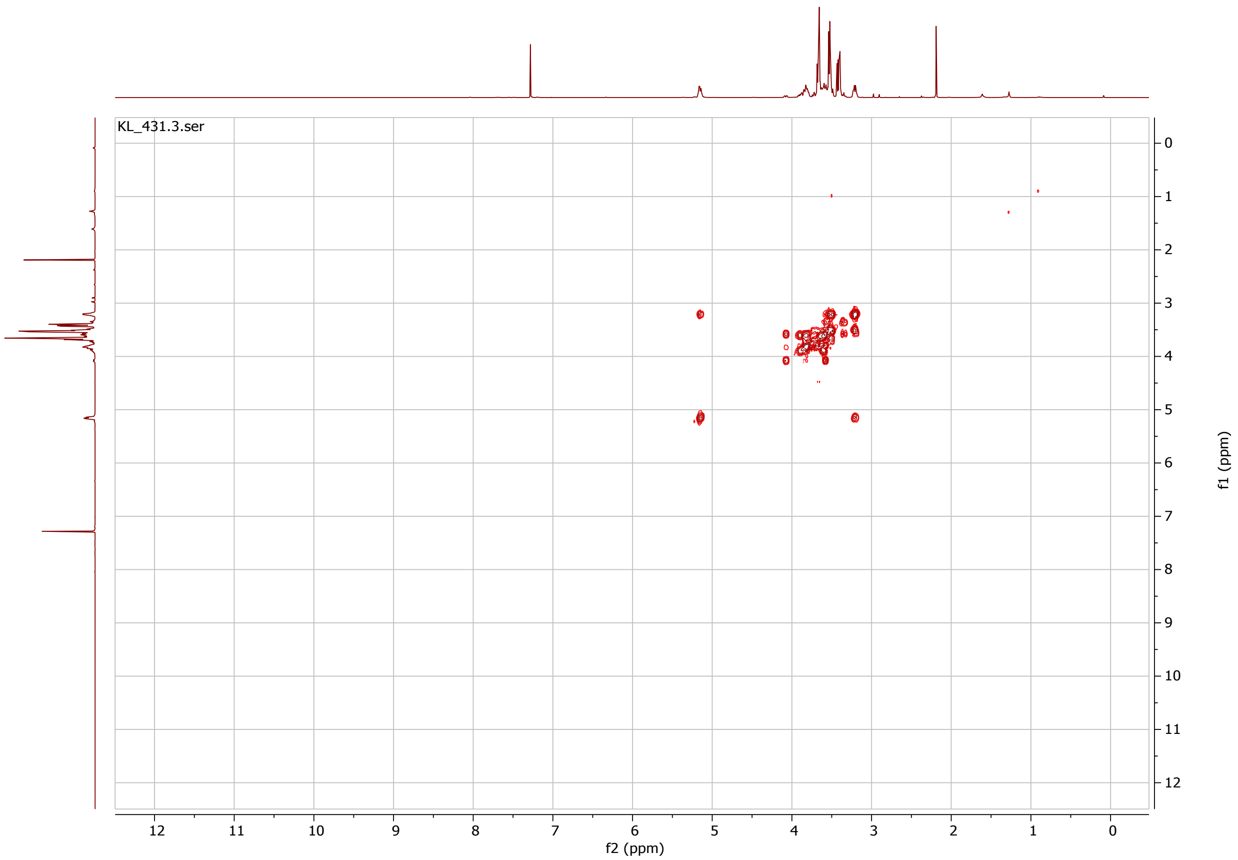

Fig. S27 2D-COSY spectrum of **4d** in $CDCl_3$ at 25 °C

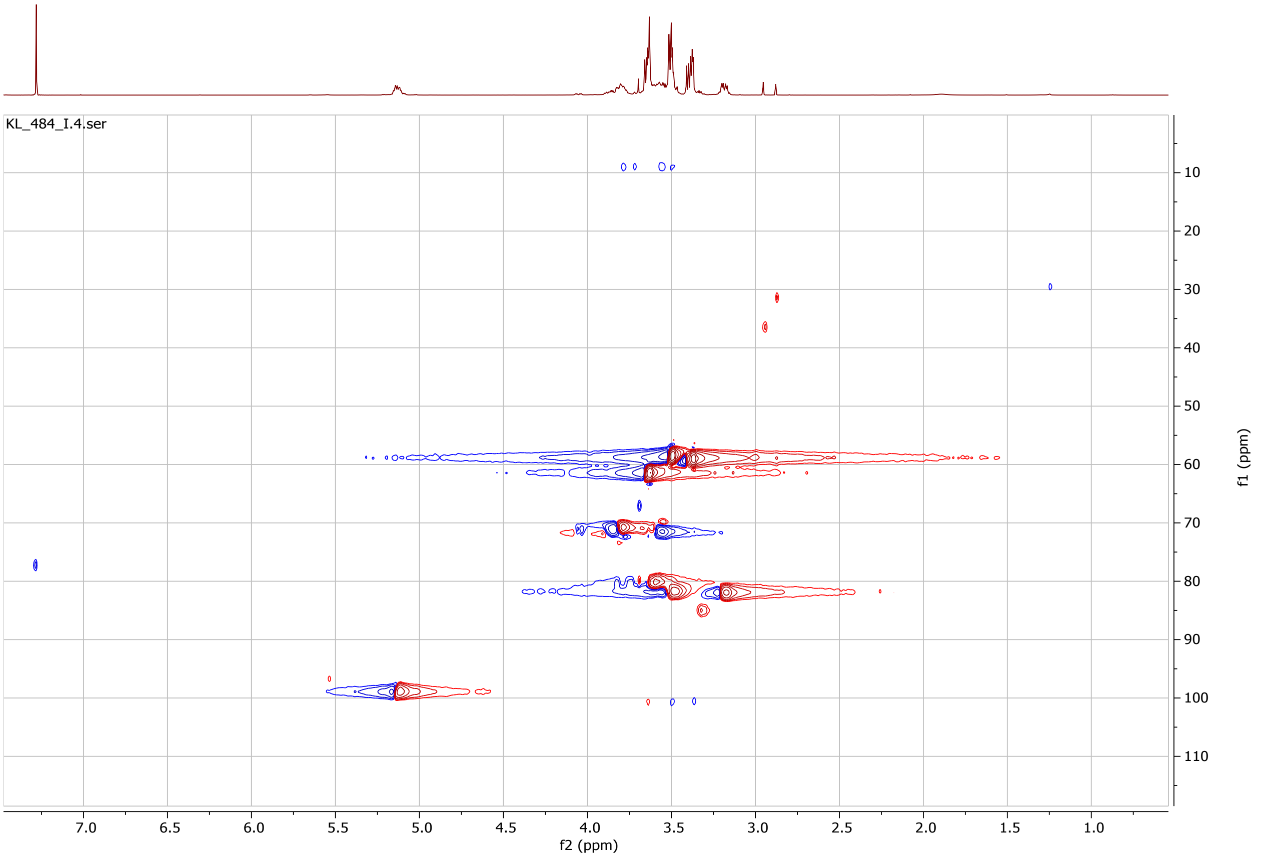

Fig. S28 2D-HSQC spectrum of **4d** in $CDCl_3$ at 25 °C

6^A-Chloro-6^A-deoxy-cyclomaltoheptaose(**2a**)

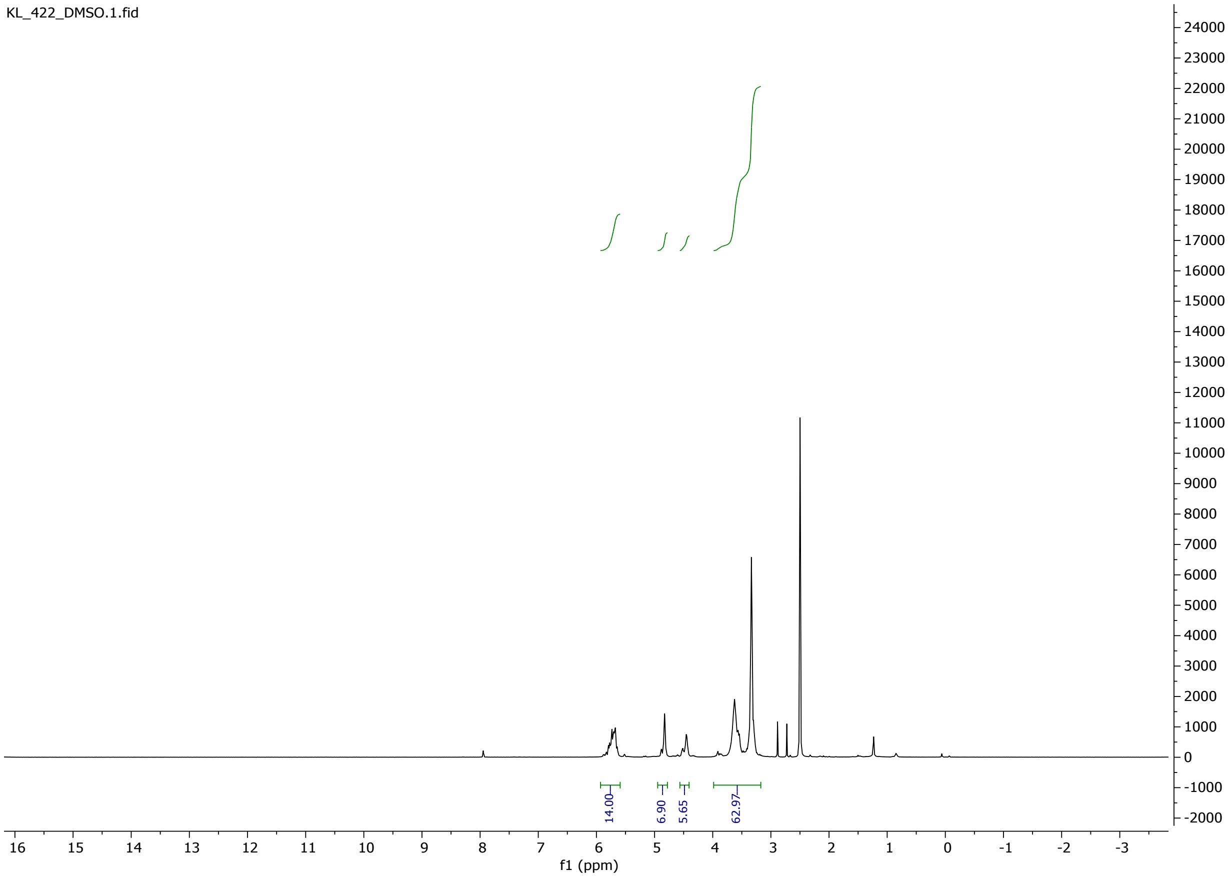

Fig. S29 ^{1}H-NMR-400 MHz spectrum of **2a** in DMSO-d_6 at 25 °C

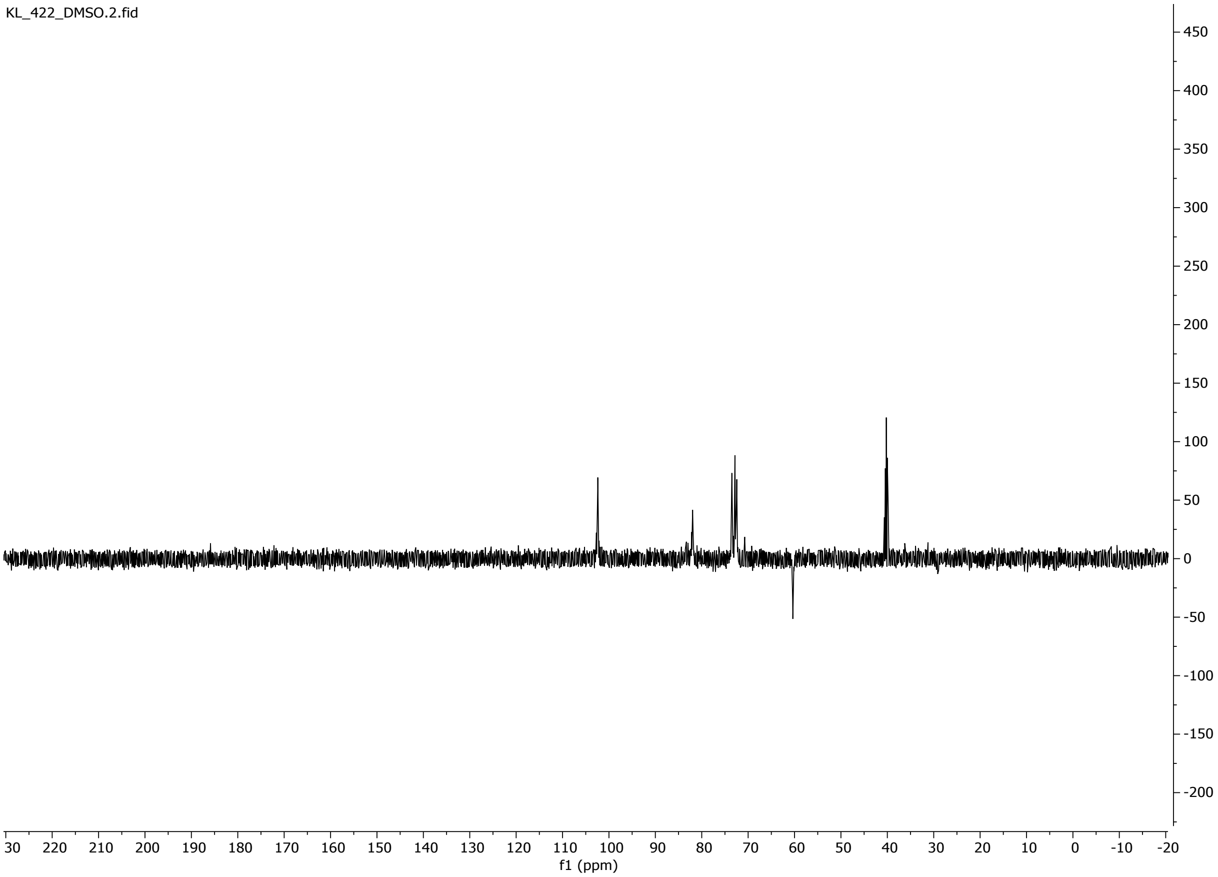

Fig. S30 ^{13}C-DEPT-101 MHz spectrum of **2a** in DMSO-d_6 at 25 °C

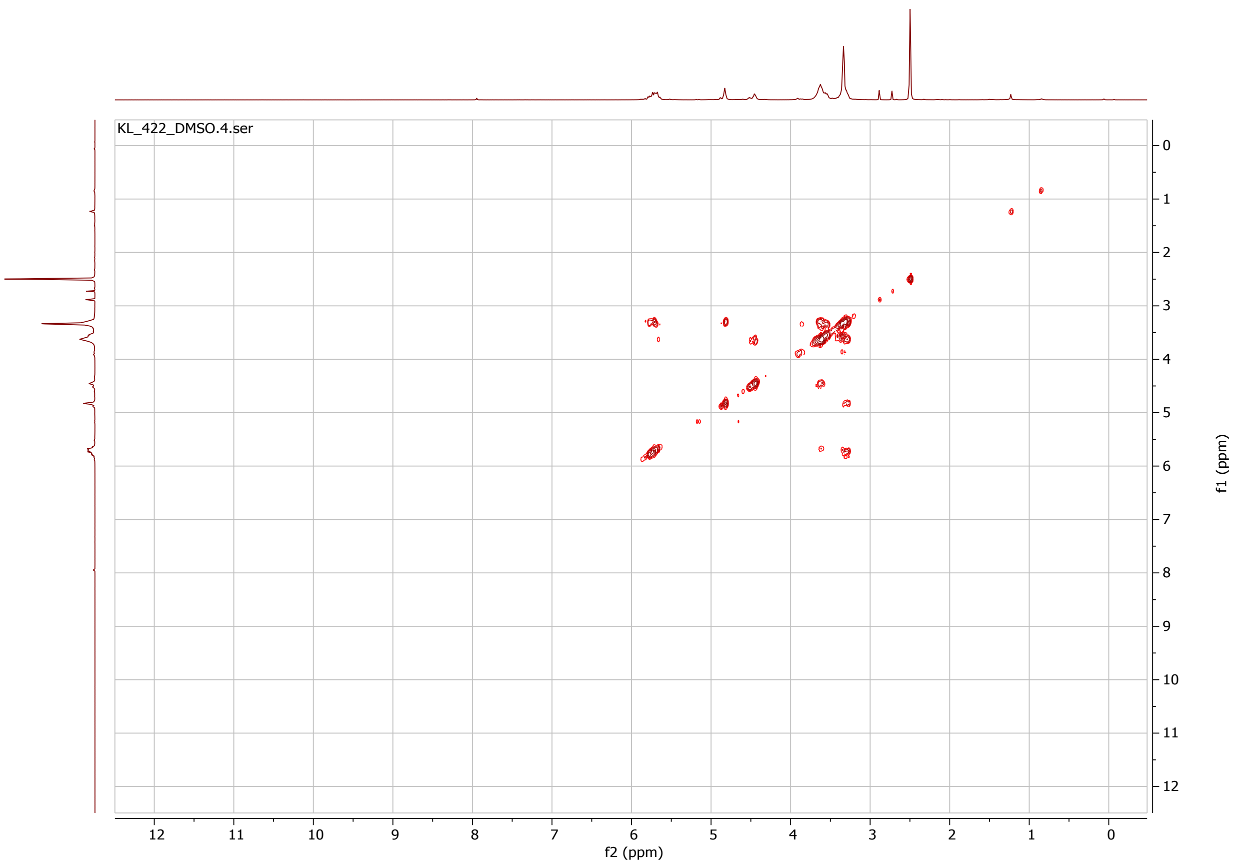

Fig. S31 2D-COSY spectrum of **2a** in DMSO-d_6 at 25 °C

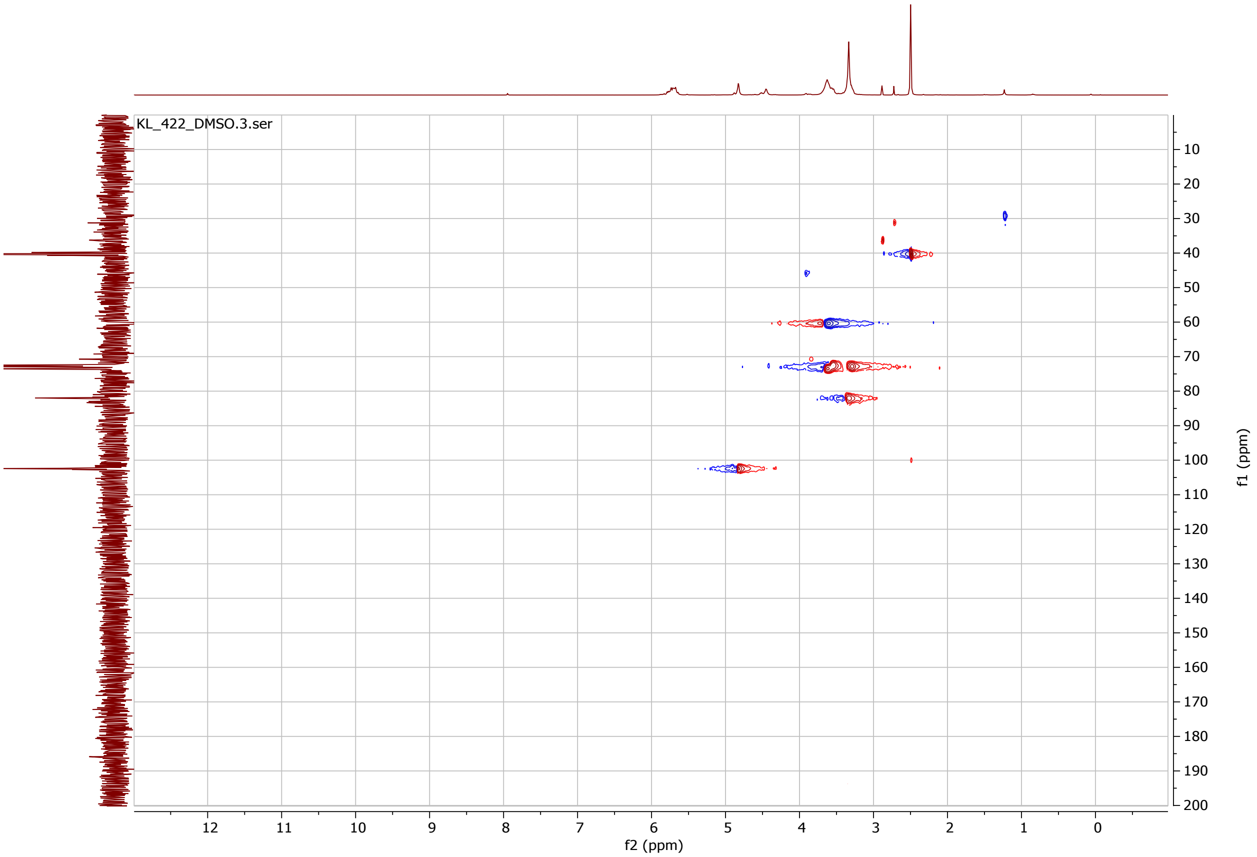

Fig. S32 2D-HSQC spectrum of **2a** in DMSO-d_6 at 25 °C

6^A^-Bromo-6^A^-deoxy-cyclomaltoheptaose (**3a**)

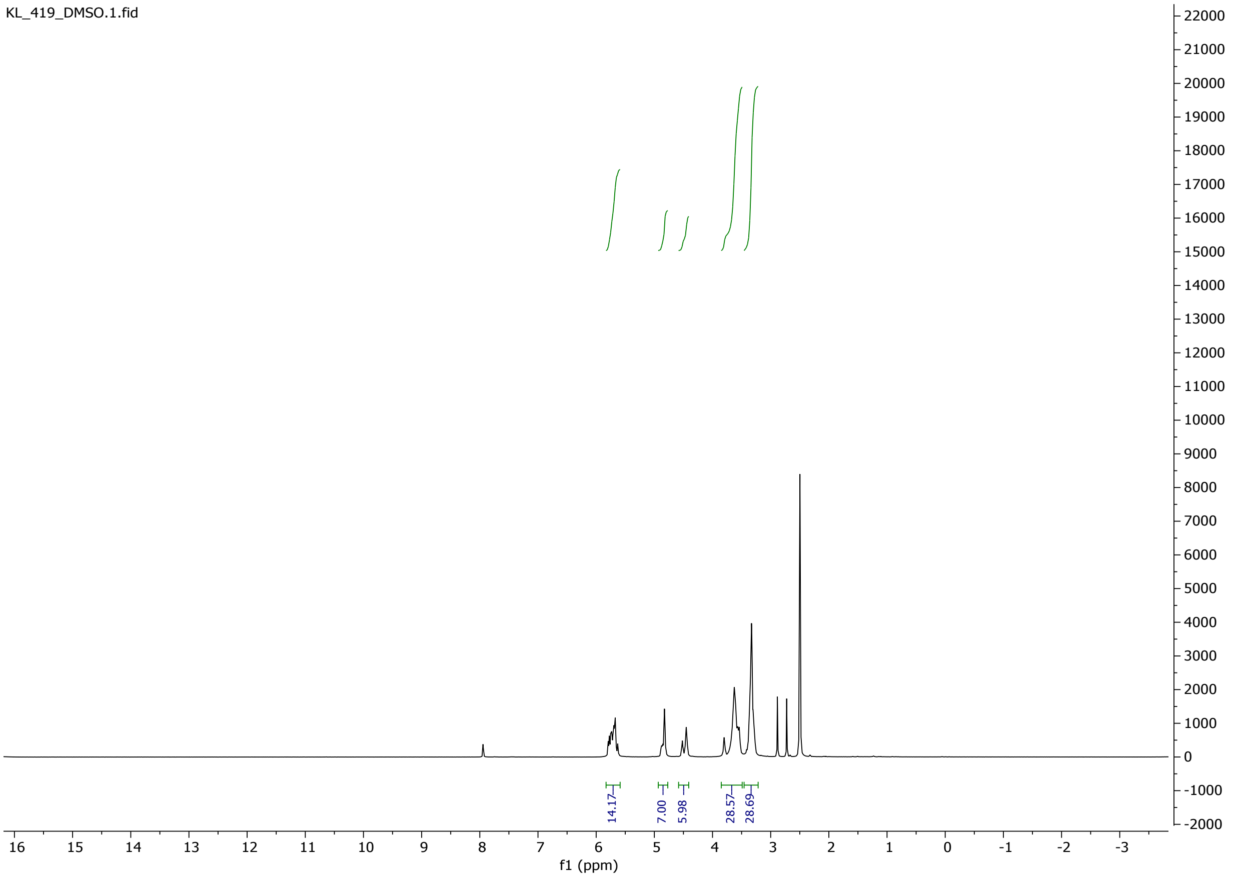

Fig. S33 ^{1}H-NMR-400 MHz spectrum of **3a** in DMSO-d_6 at 25 °C

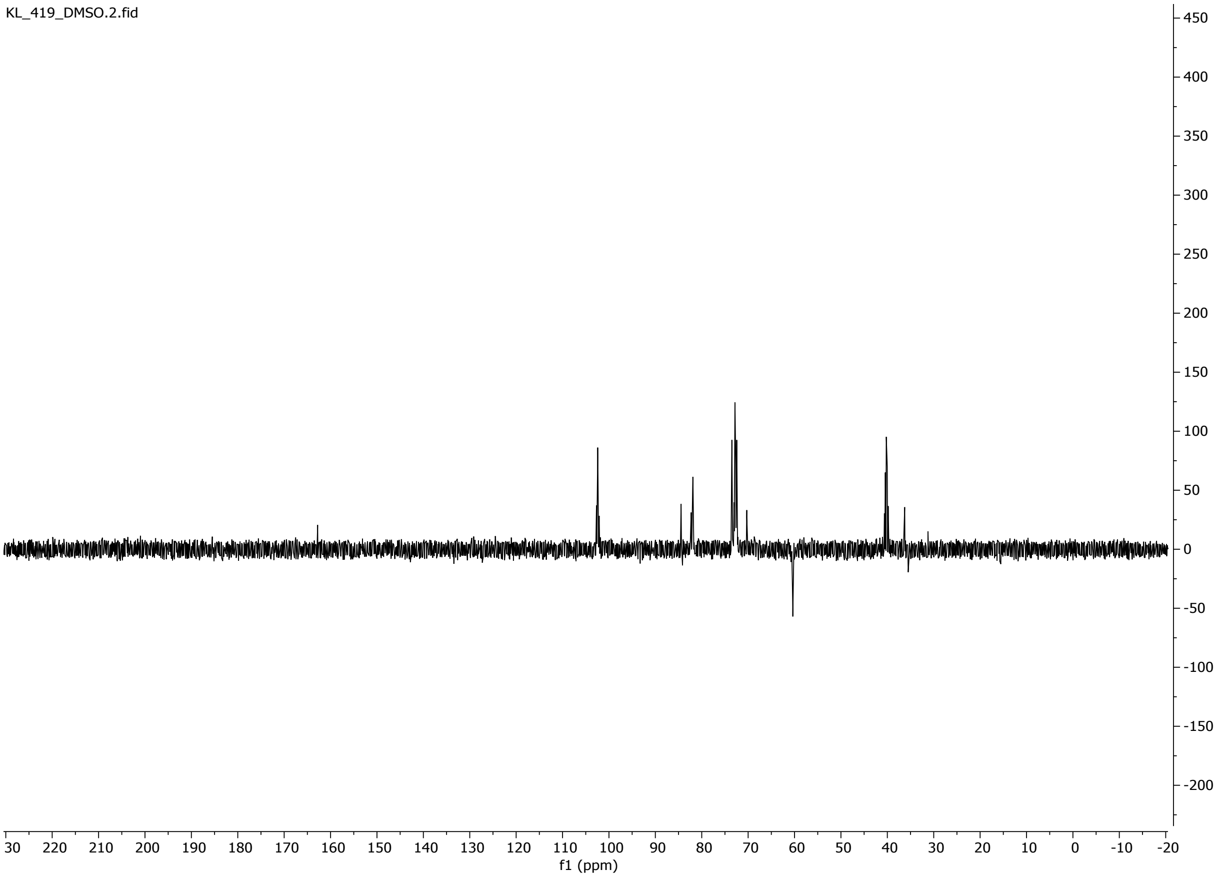

Fig. S34 ^{13}C-DEPT-101 MHz spectrum of **3a** in DMSO-d_6 at 25 °C

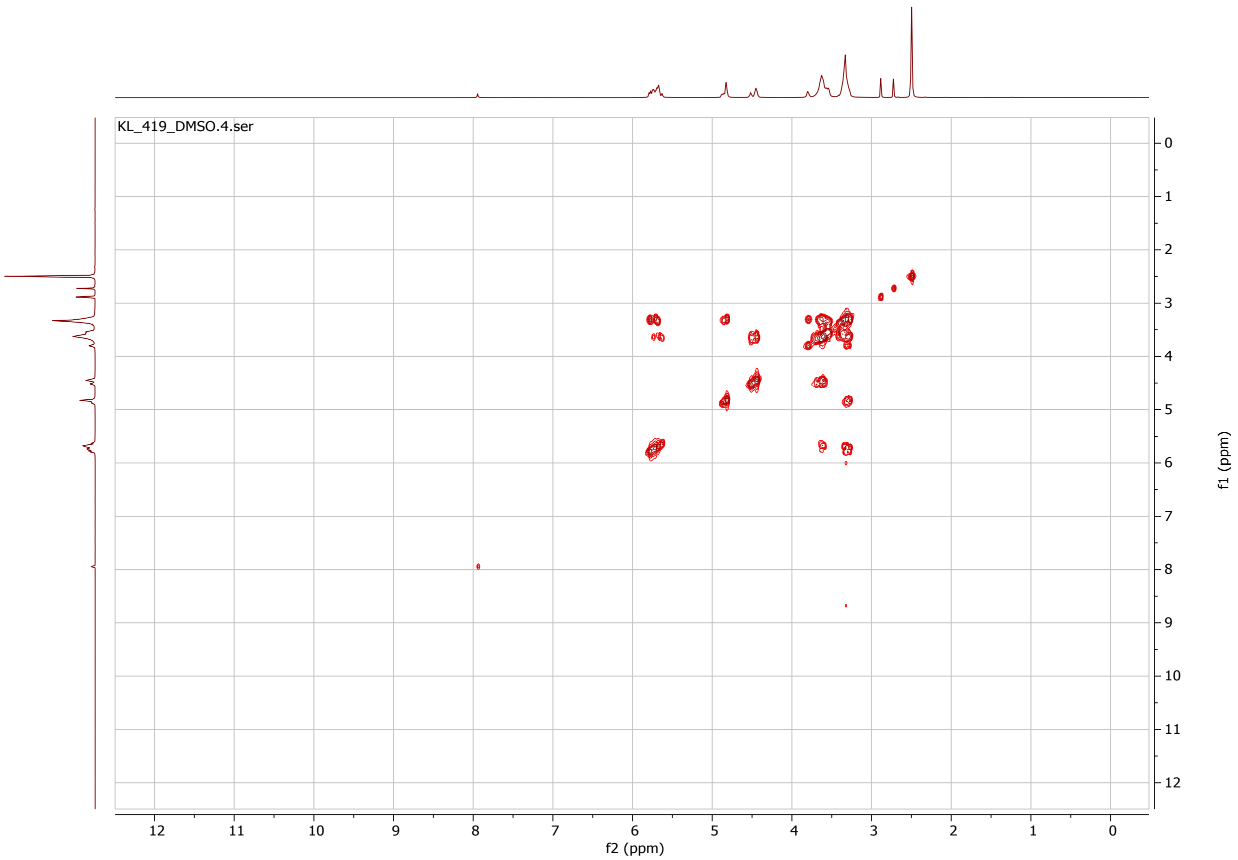

Fig. S35 2D-COSY spectrum of **3a** in DMSO-d_6 at 25 °C

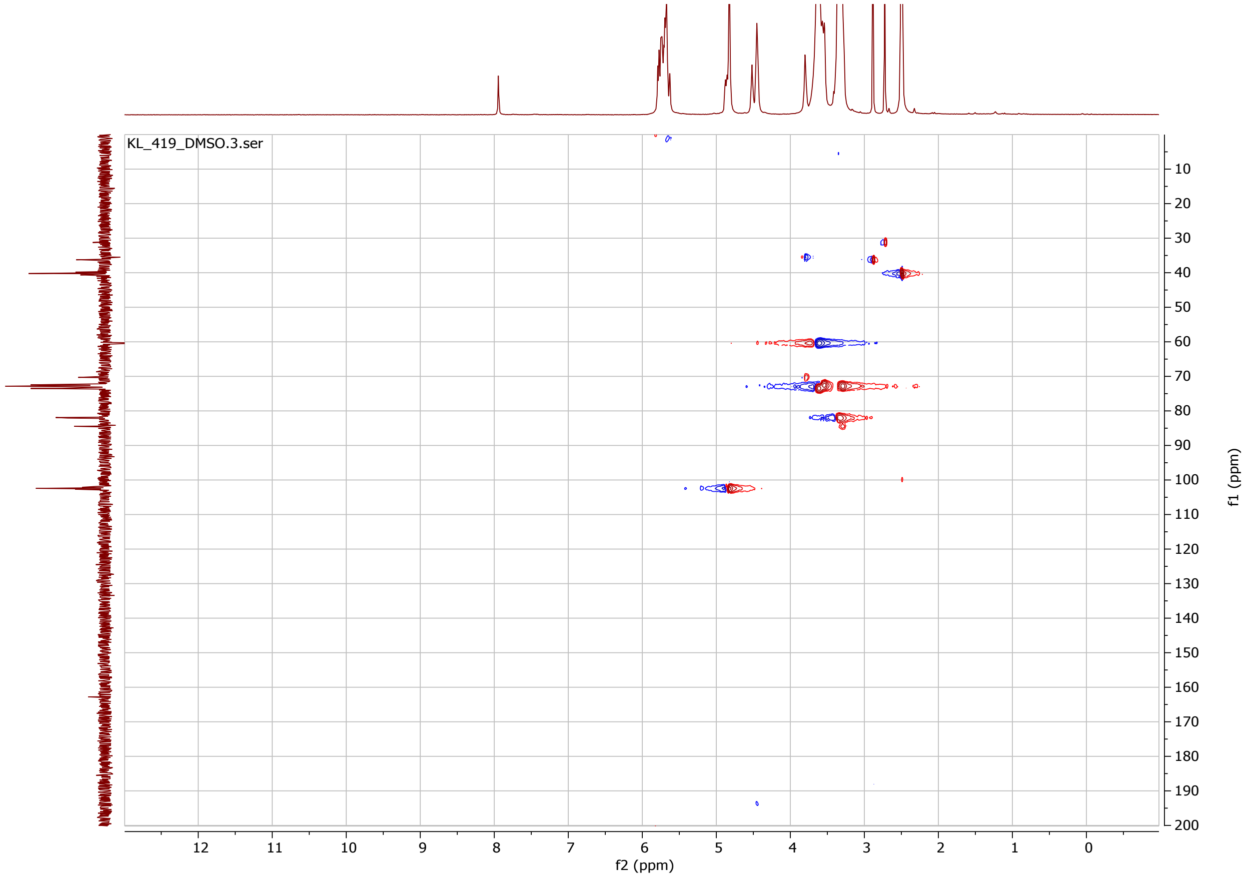

Fig. S36 2D-HSQC spectrum of **3a** in DMSO-d_6 at 25 °C

6^A-Deoxy-6^A-iodo-cyclomaltoheptaose (**4a**)

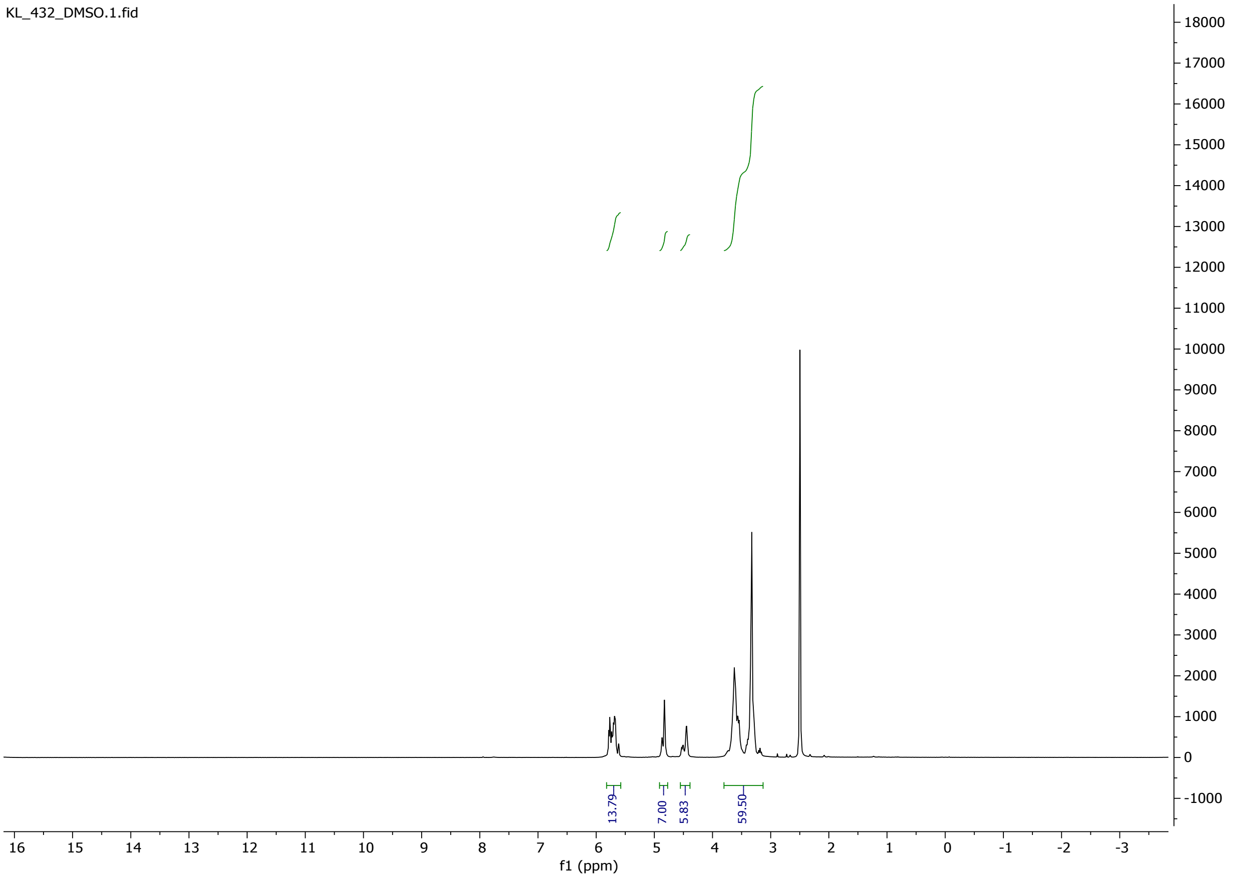

Fig. S37 ^{1}H-NMR-400 MHz spectrum of **4a** in DMSO-d_6 at 25 °C

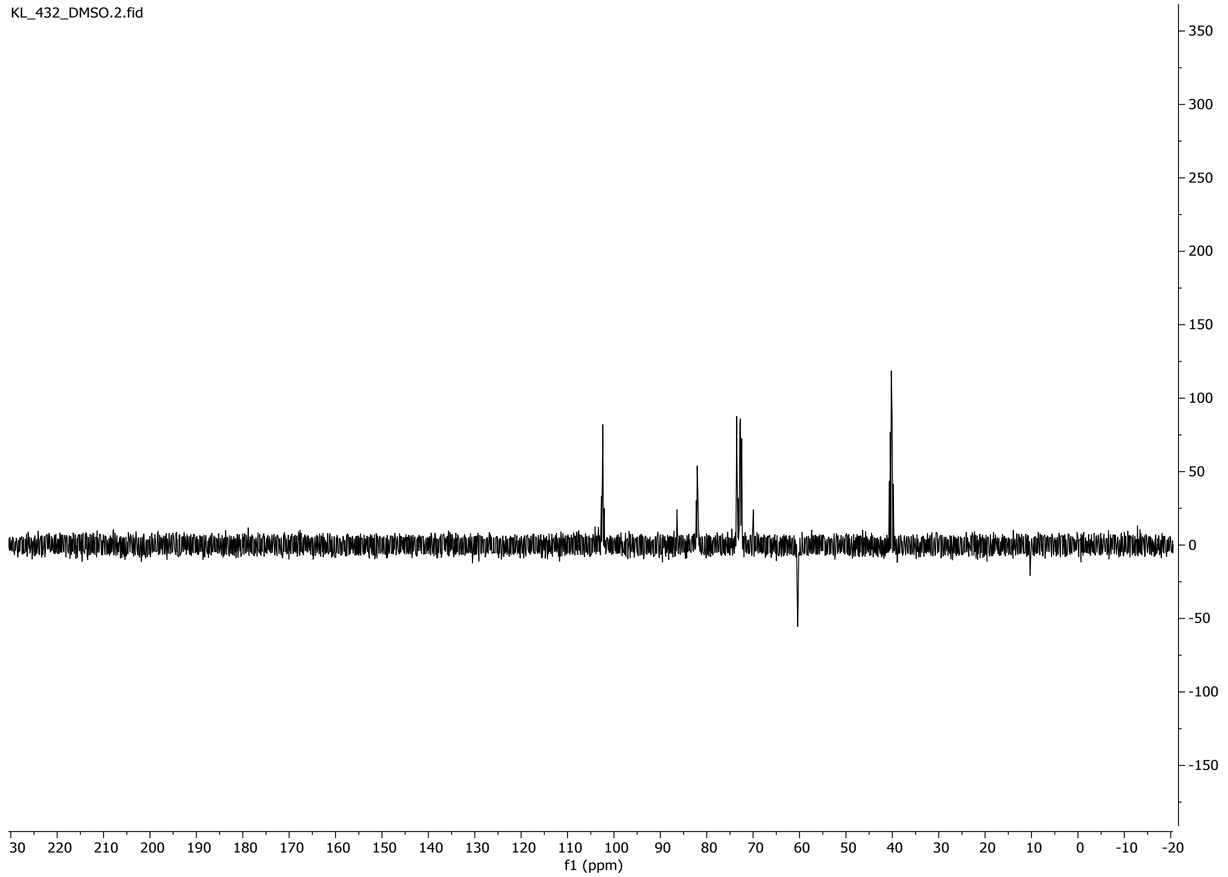

Fig. S38 ^{13}C-DEPT-101 MHz spectrum of **4a** in DMSO-d_6 at 25 °C

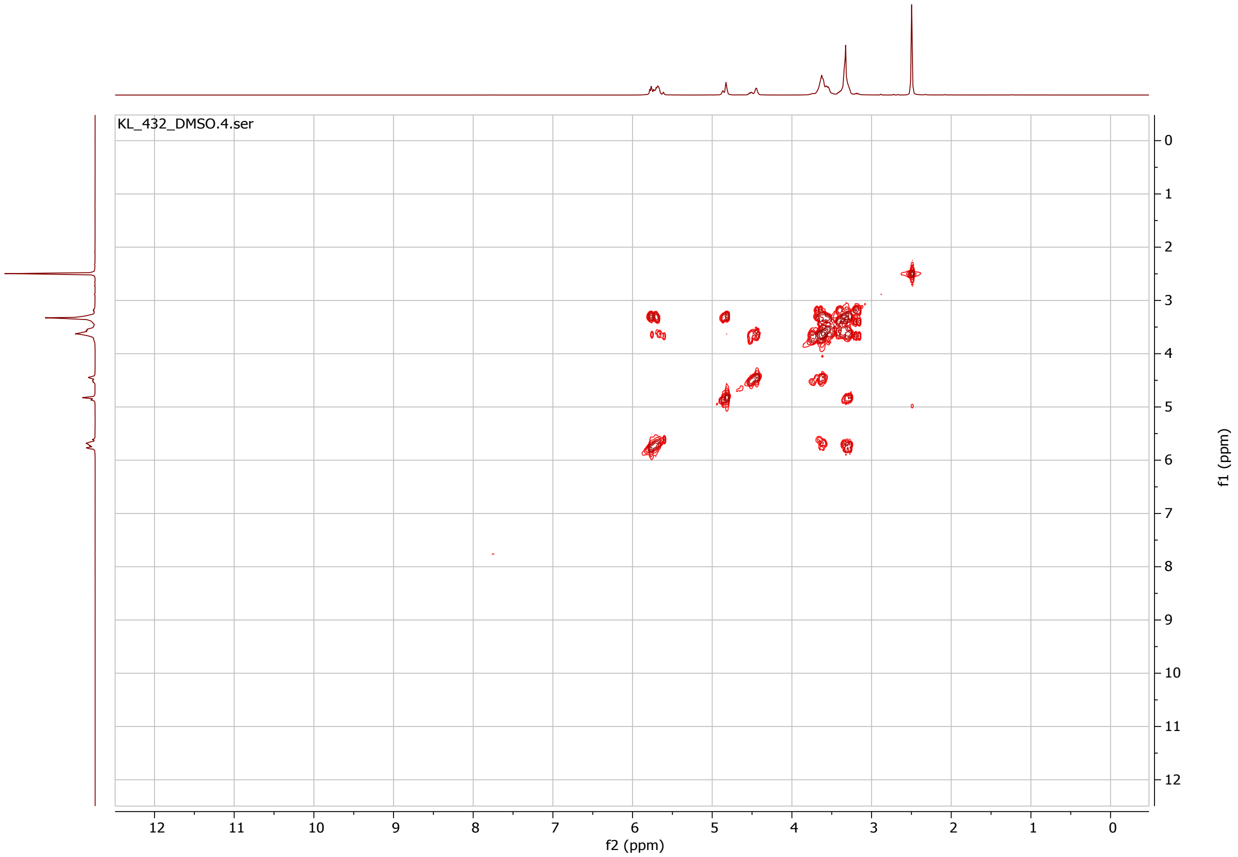

Fig. S39 2D-COSY spectrum of **4a** in DMSO-d_6 at 25 °C

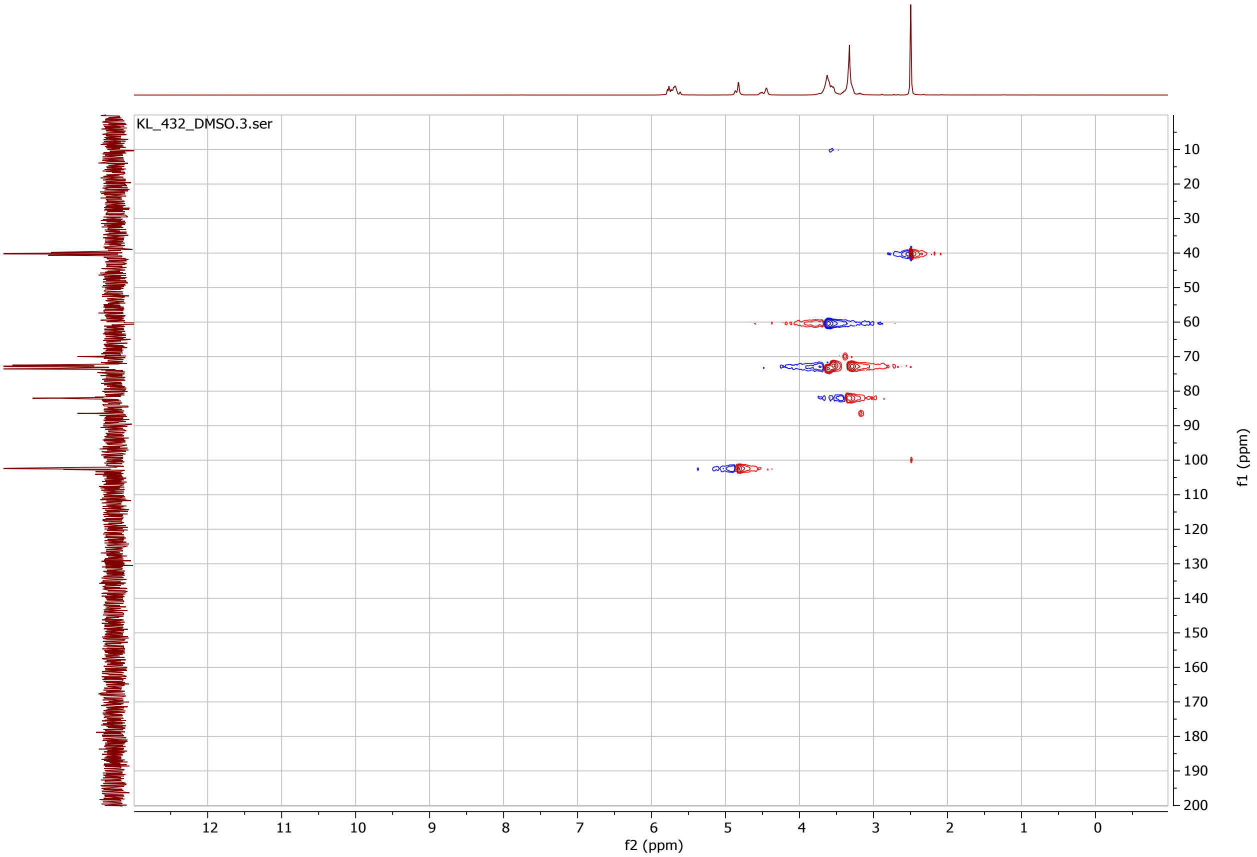

Fig. S40 2D-HSQC spectrum of **4a** in DMSO-d_6 at 25 °C

6^A-Chloro-6^A-deoxy-6^B-G-hexa-*O*-methyl-cyclomaltoheptaose (**2b**)

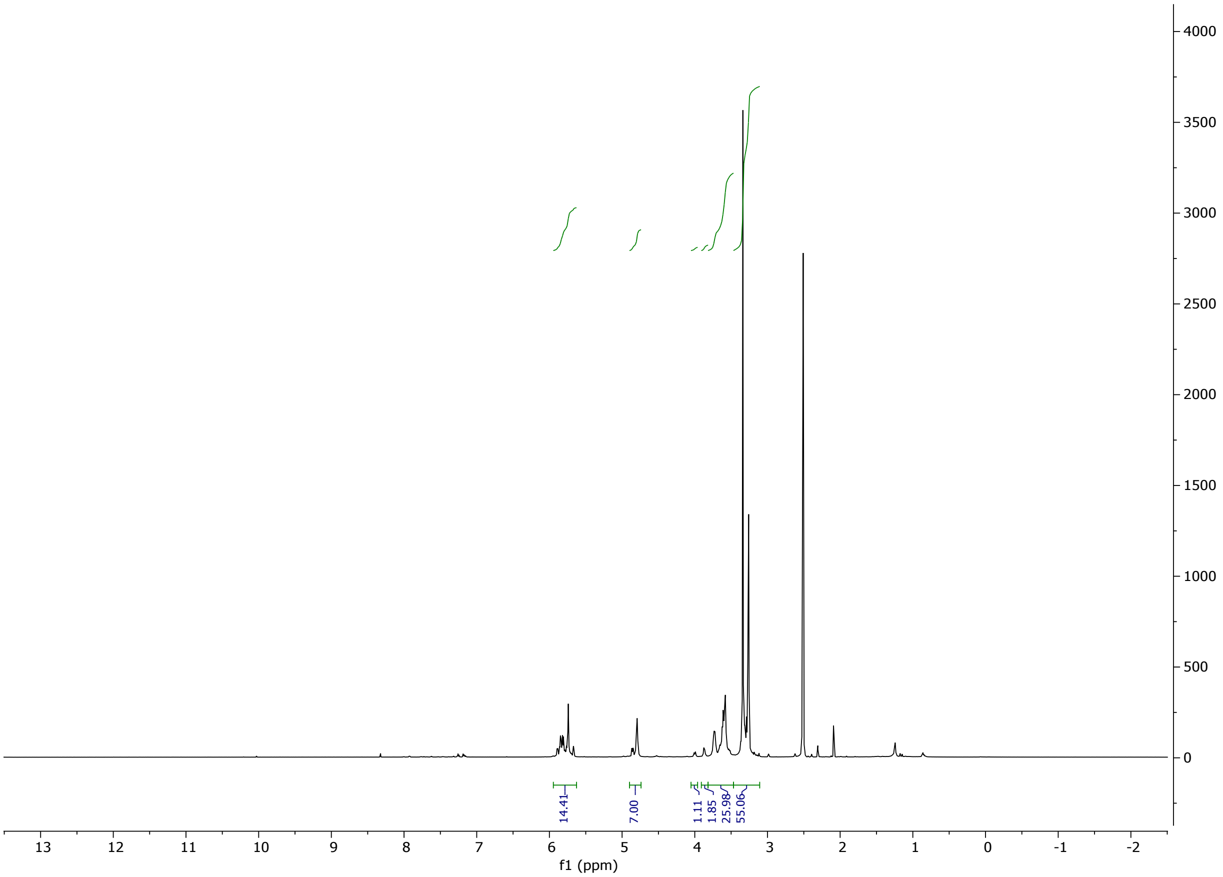

Fig. S41 ^{1}H-NMR-400 MHz spectrum of **2b** in DMSO-d_6 at 25 °C

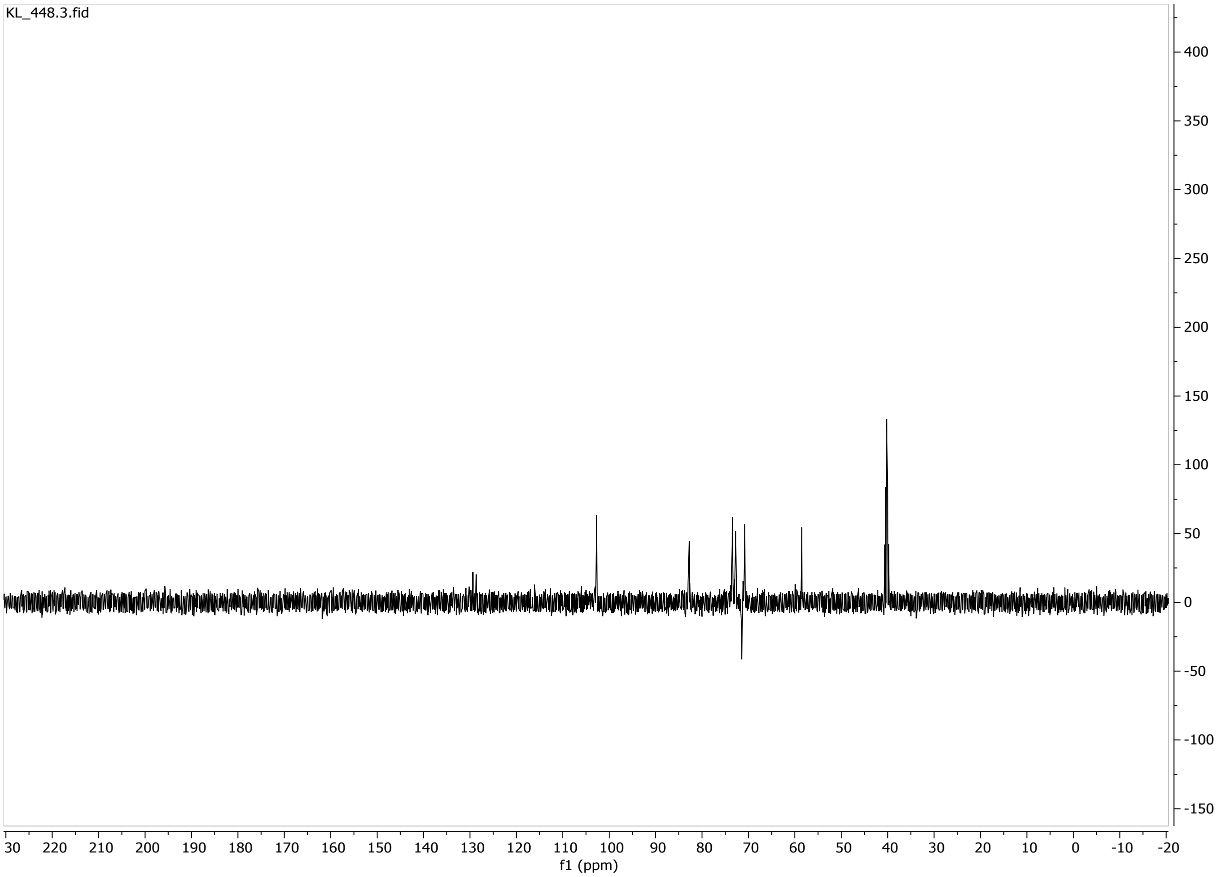

Fig. S42 ^{13}C-DEPT-101 MHz spectrum of **2b** in DMSO-d_6 at 25 °C

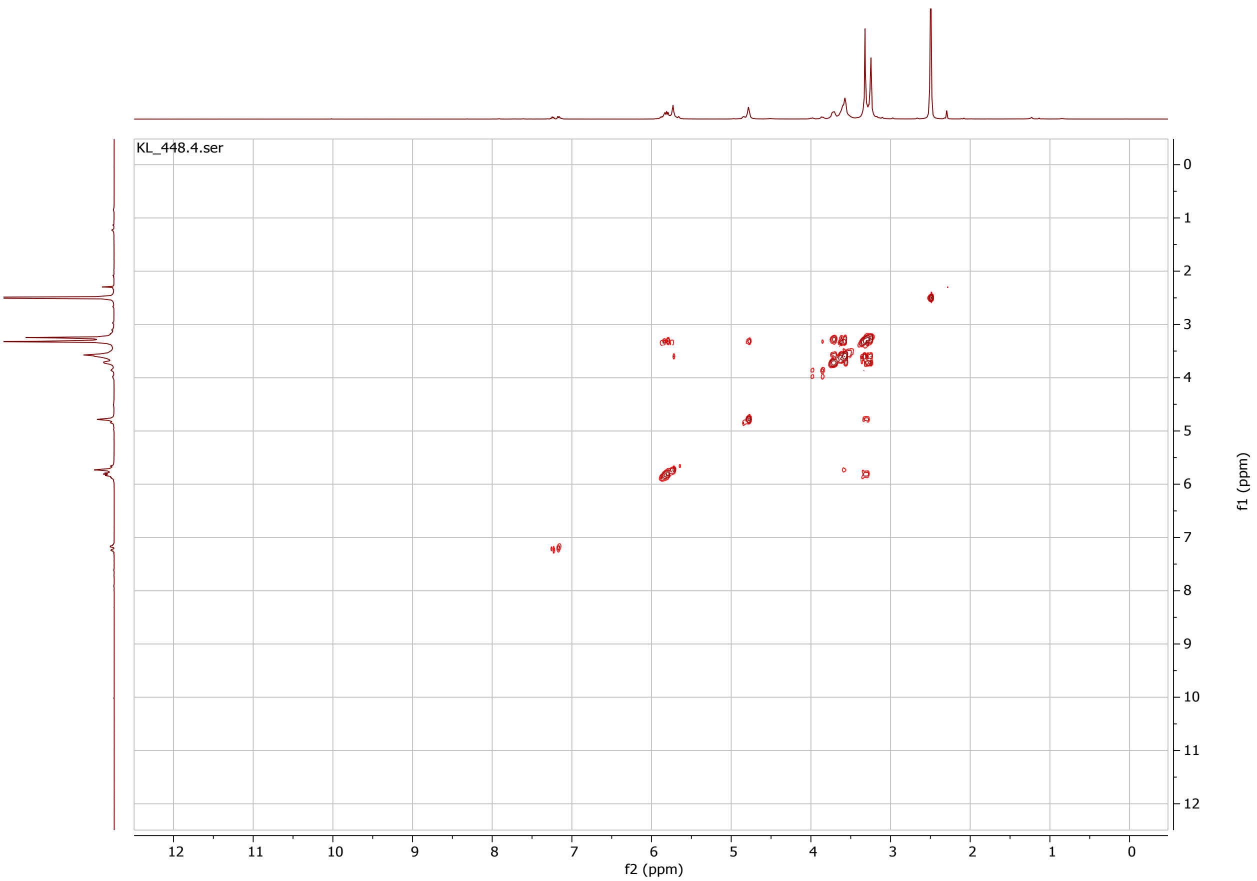

Fig. S43 2D-COSY spectrum of **2b** in DMSO-d_6 at 25 °C

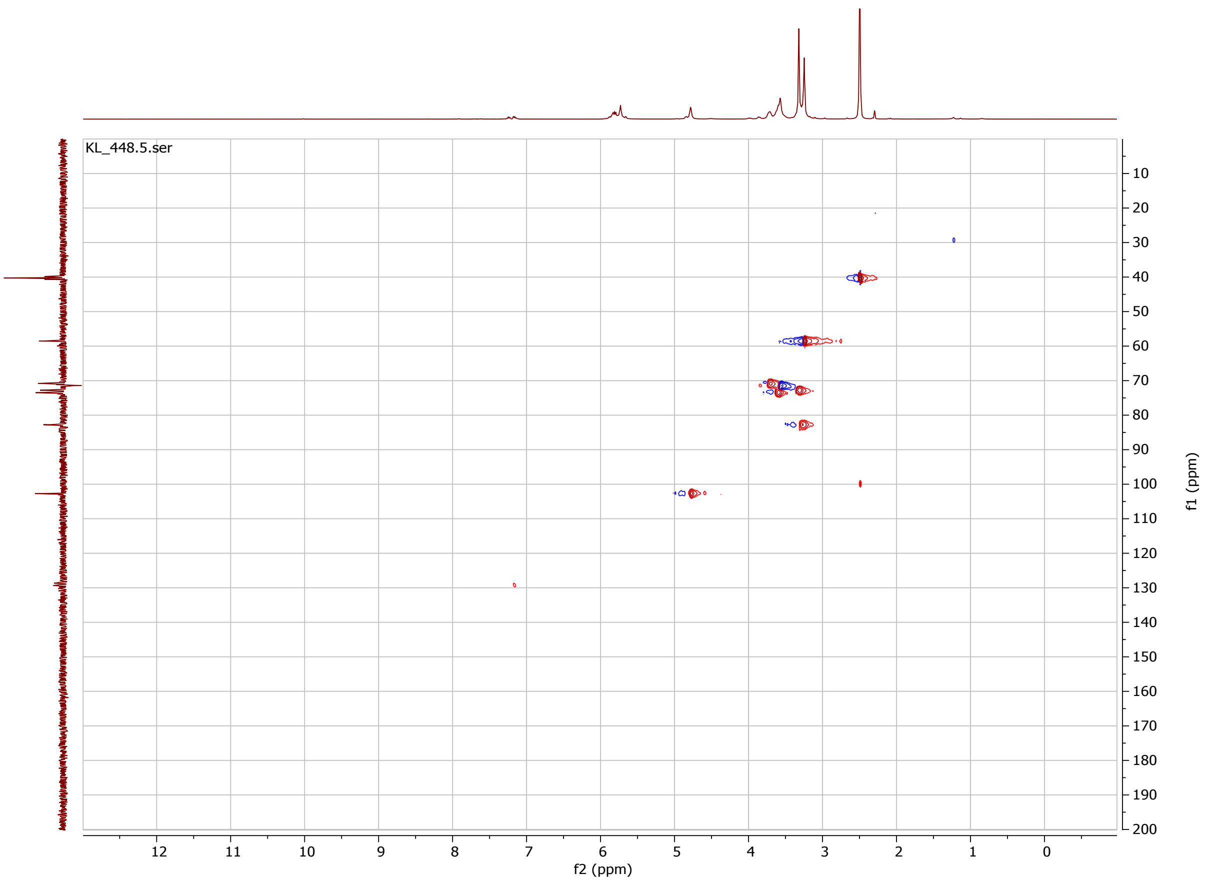

Fig. S44 2D-HSQC spectrum of **2b** in DMSO-d_6 at 25 °C

6^A-Bromo-6^A-deoxy-6^(B-G)-hexa-*O*-methyl-cyclomaltoheptaose (**3b**)

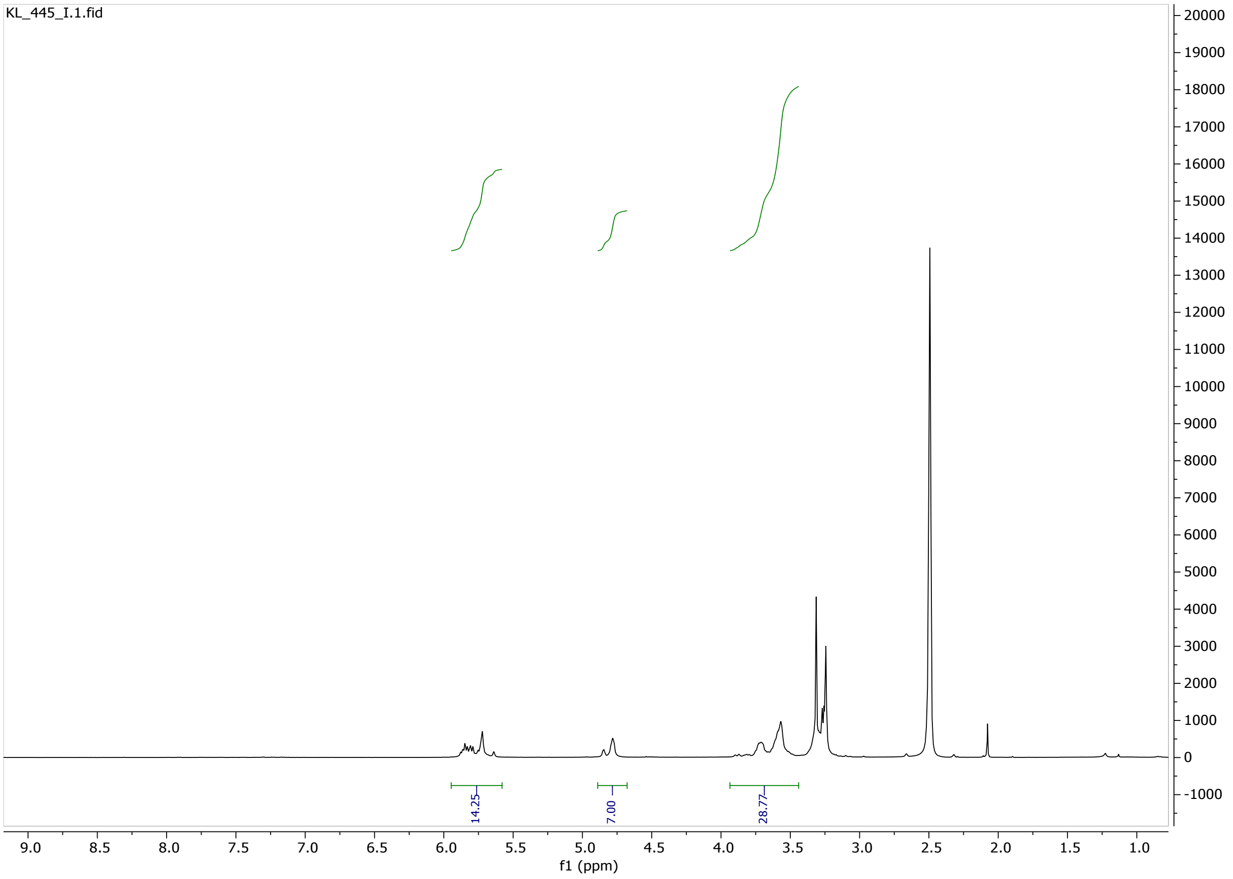

Fig. S45 H-NMR-400 MHz spectrum of **3b** in DMSO-d_6 at 25 °C

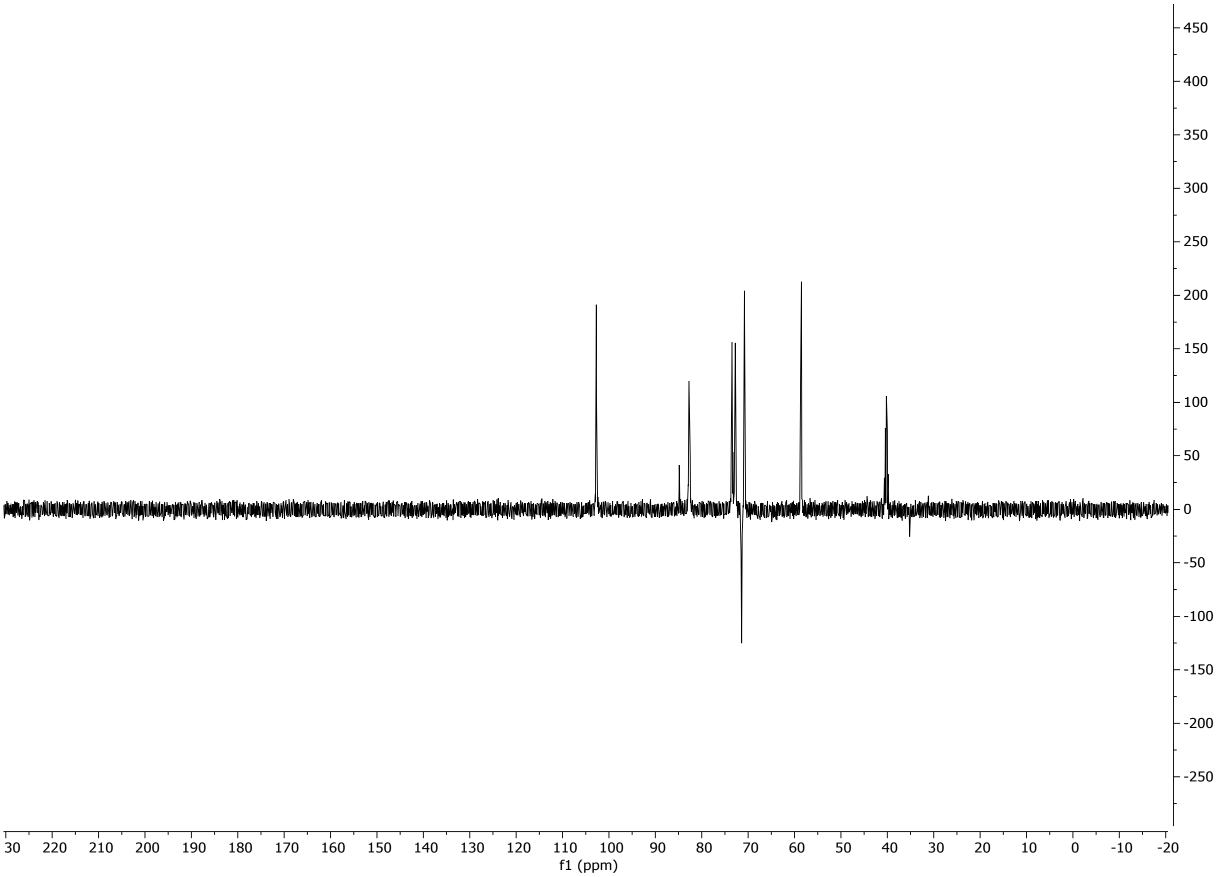

Fig. S46 ^{13}C-DEPT-101 MHz spectrum of **3b** in DMSO-d_6 at 25 °C

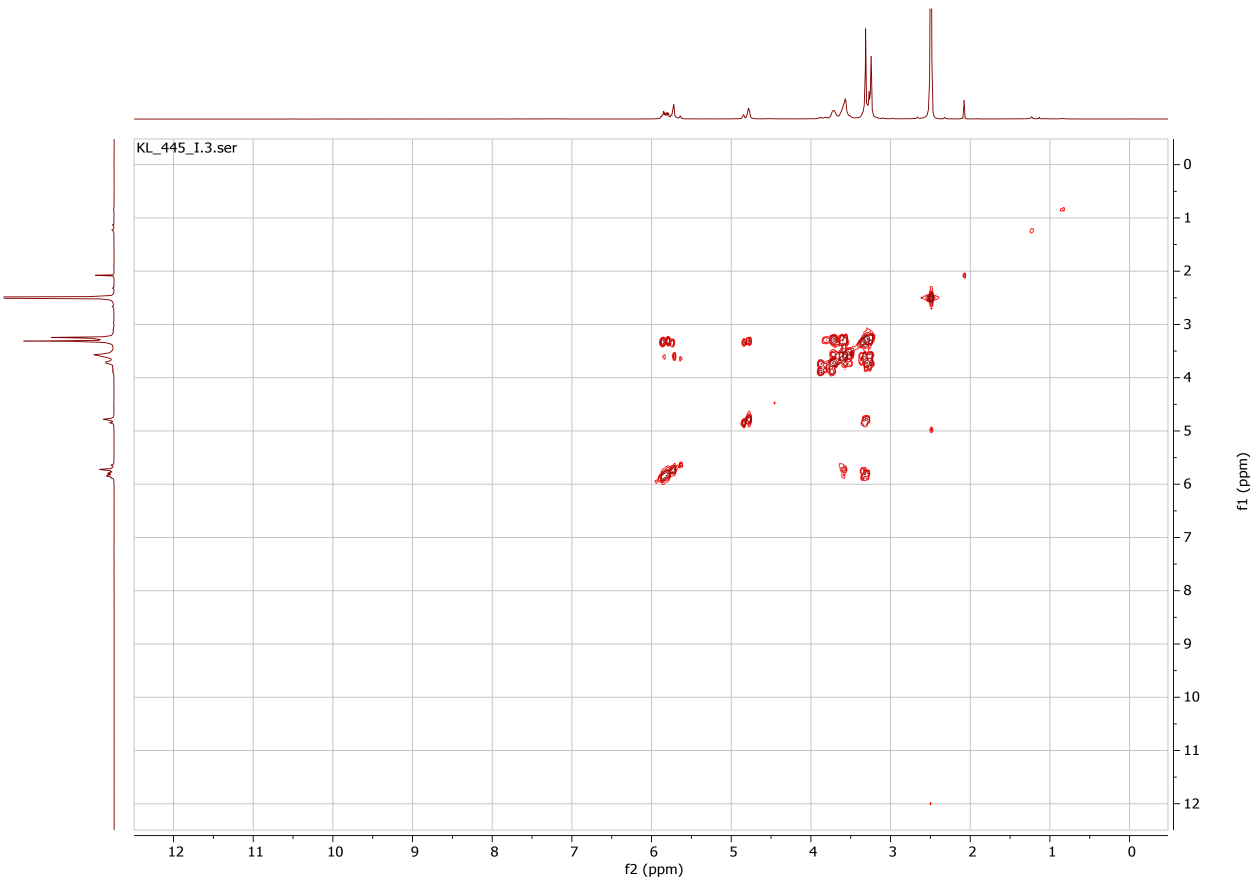

Fig. S47 2D-COSY spectrum of **3b** in DMSO-d_6 at 25 °C

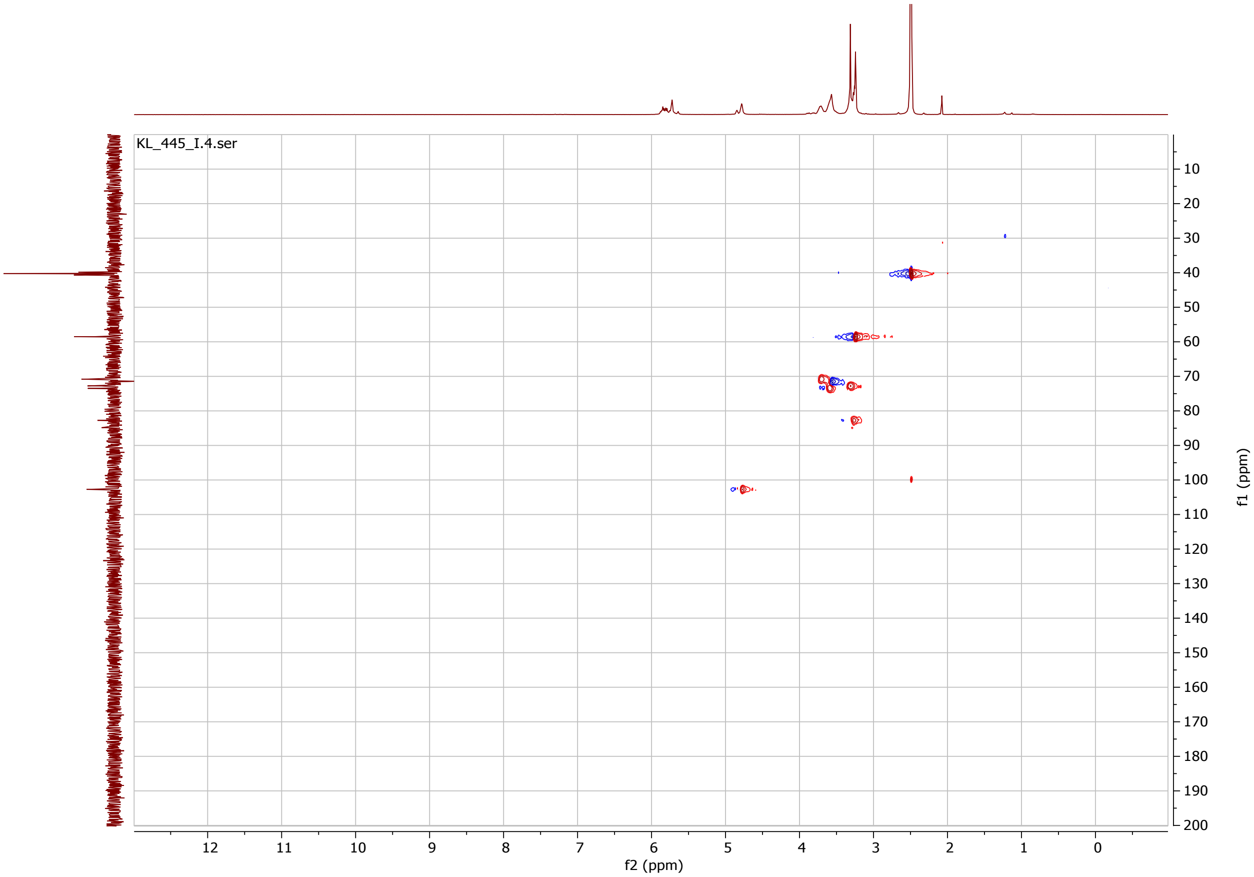

Fig. S48 2D-HSQC spectrum of **3b** in DMSO-d_6 at 25 °C

6^{A}-Deoxy-6^{A}-iodo-6^{B-G}-hexa-*O*-methyl-cyclomaltoheptaose (**4b**)

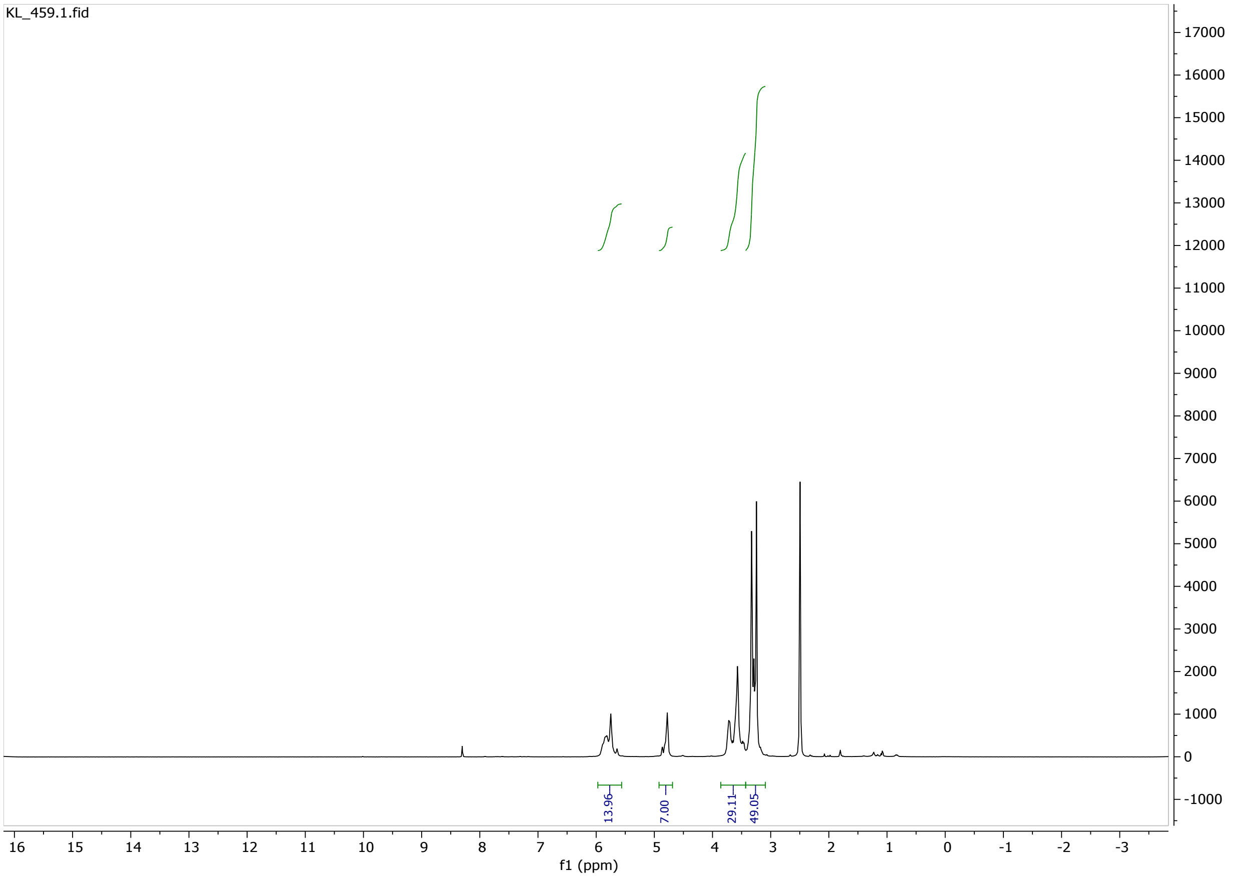

Fig. S49 ^{1}H-NMR-400 MHz spectrum of **4b** in DMSO-d_6 at 25 °C

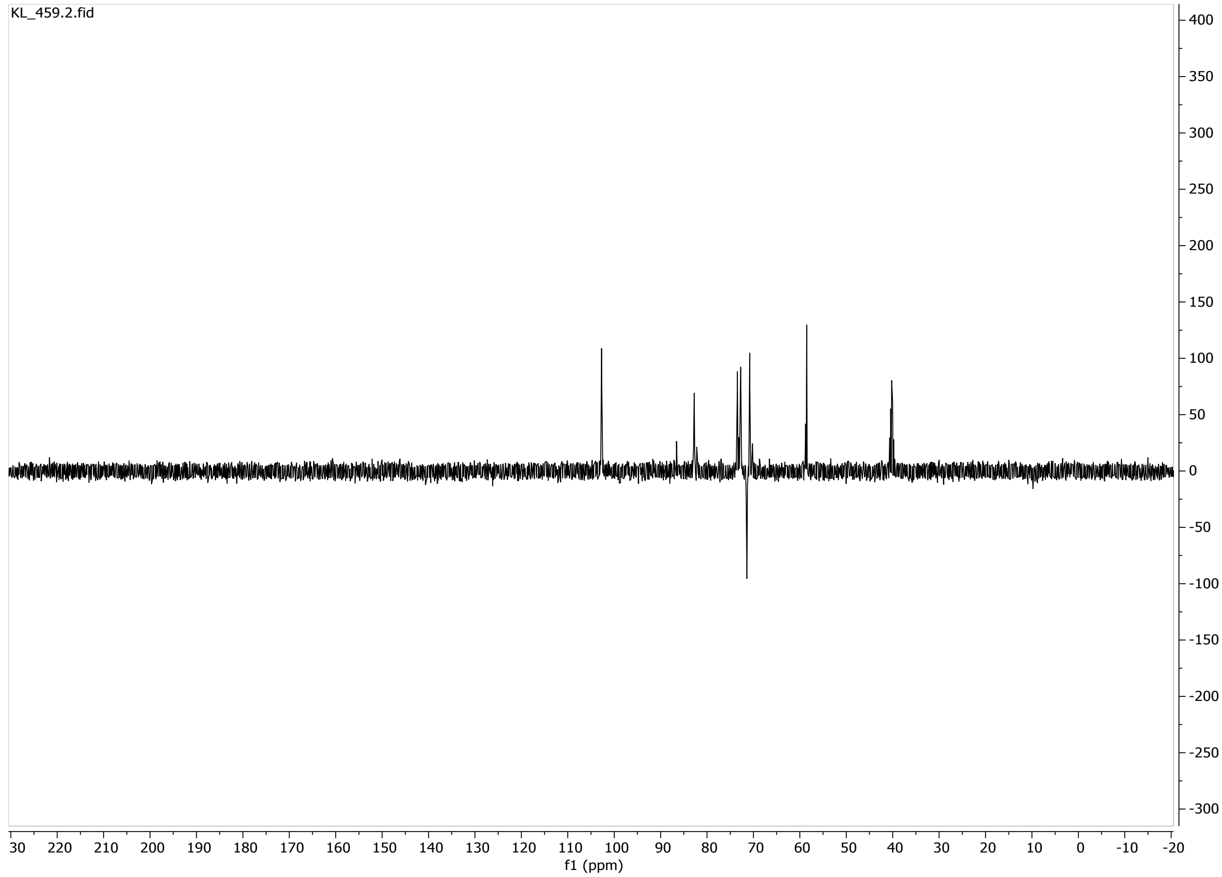

Fig. S50 ^{13}C-DEPT-101 MHz spectrum of **4b** in DMSO-d_6 at 25 °C

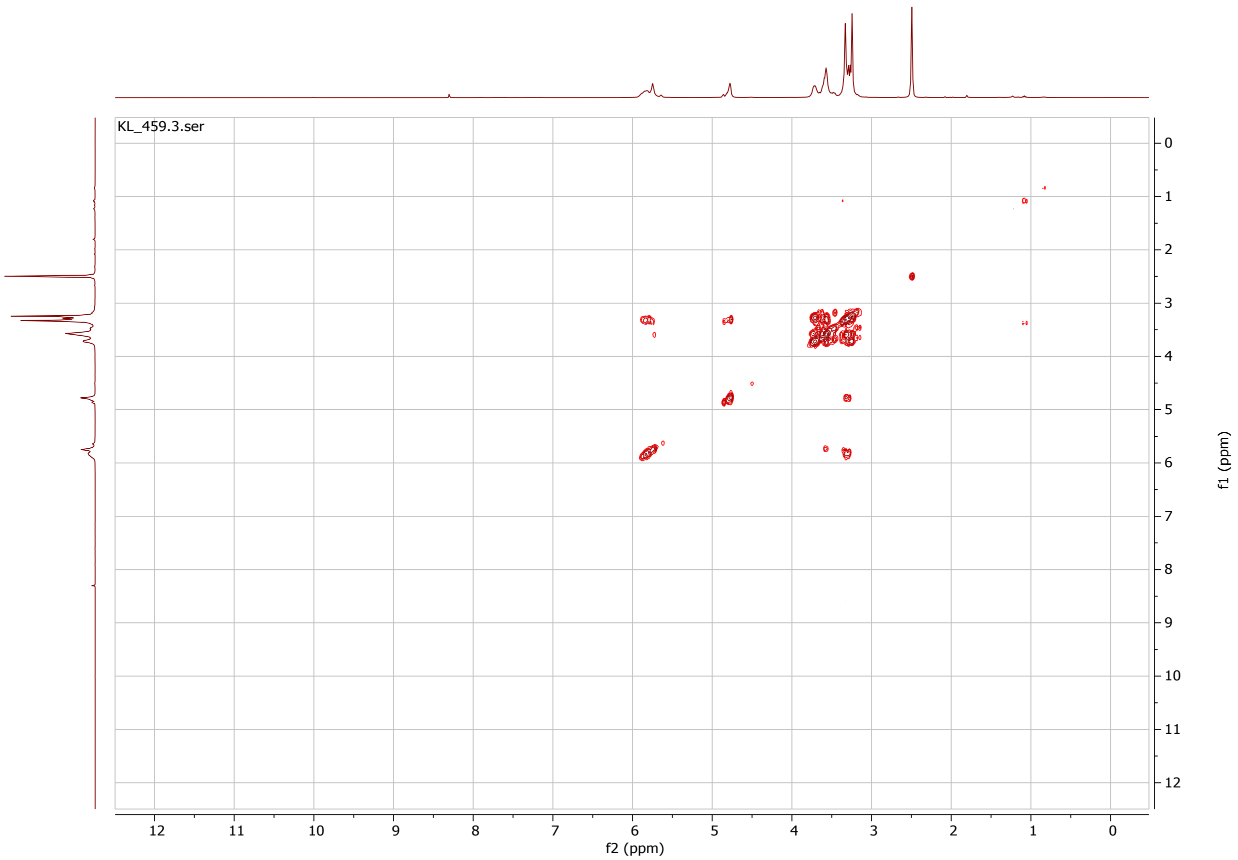

Fig. S51 2D-COSY spectrum of **4b** in DMSO-d_6 at 25 °C

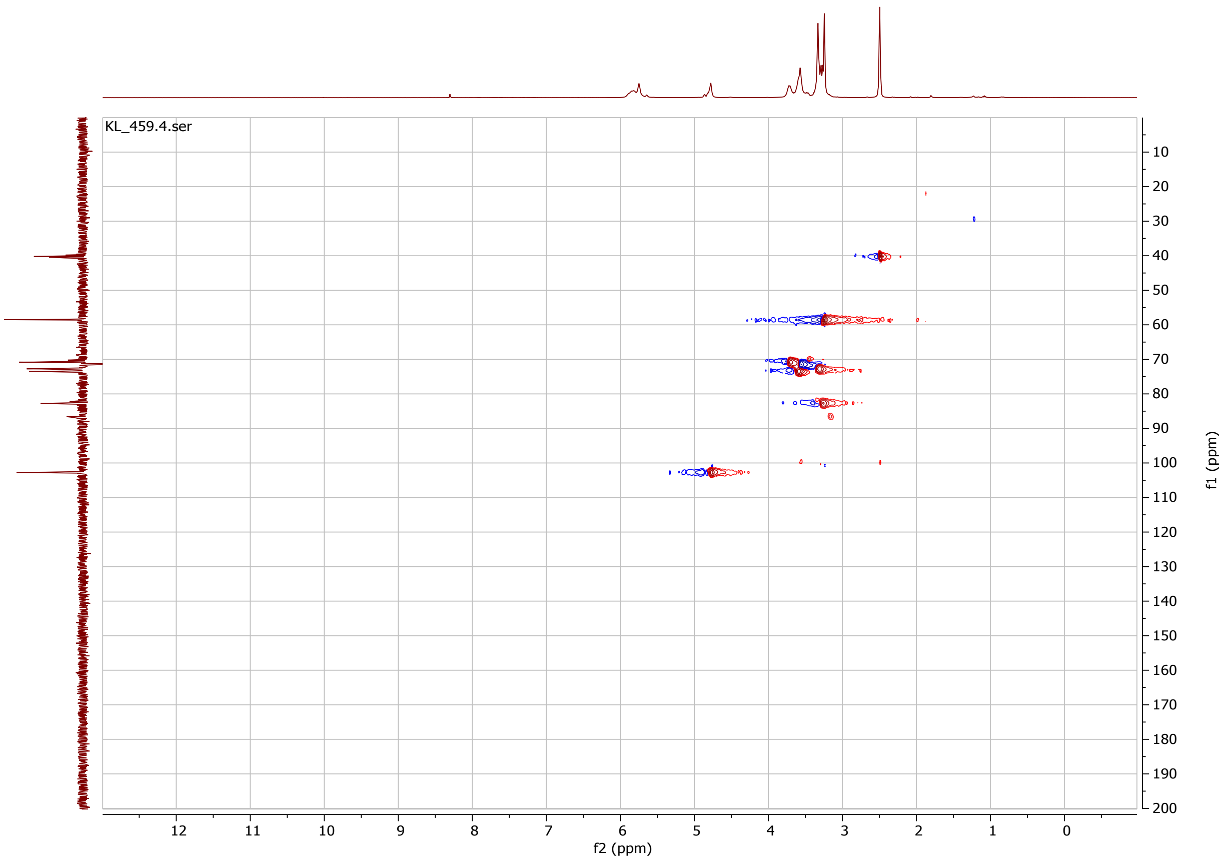

Fig. S52 2D-HSQC spectrum of **4b** in DMSO-d_6 at 25 °C

$2^{A\text{-}G}$,$3^{A\text{-}G}$-Tetradeca-*O*-acetyl-6^{A}-amino-6^{A}-deoxy-$6^{B\text{-}G}$-hexa-*O*-methyl-cyclomaltoheptaose (**1e**)

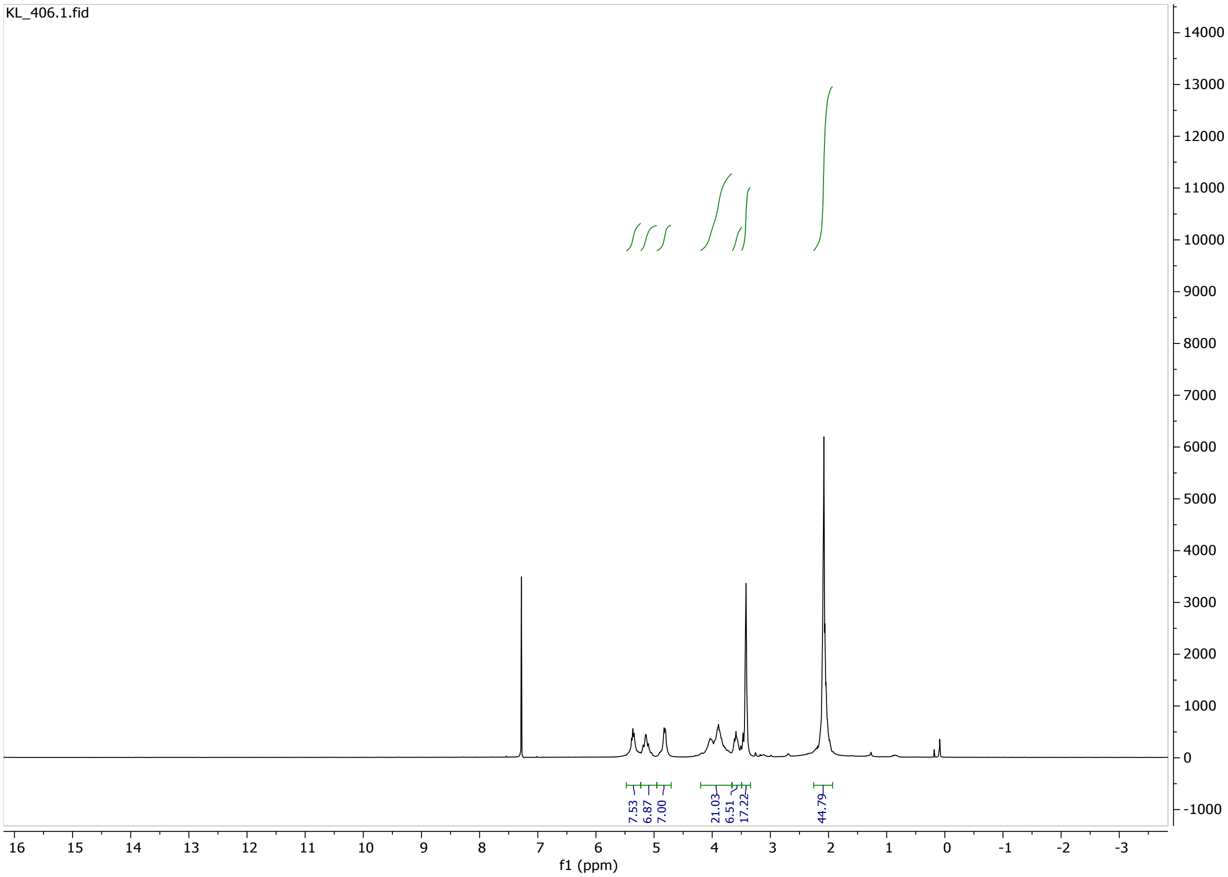

Fig. S53 ^{1}H-NMR-400 MHz spectrum of **1e** in $CDCl_3$ at 25 °C

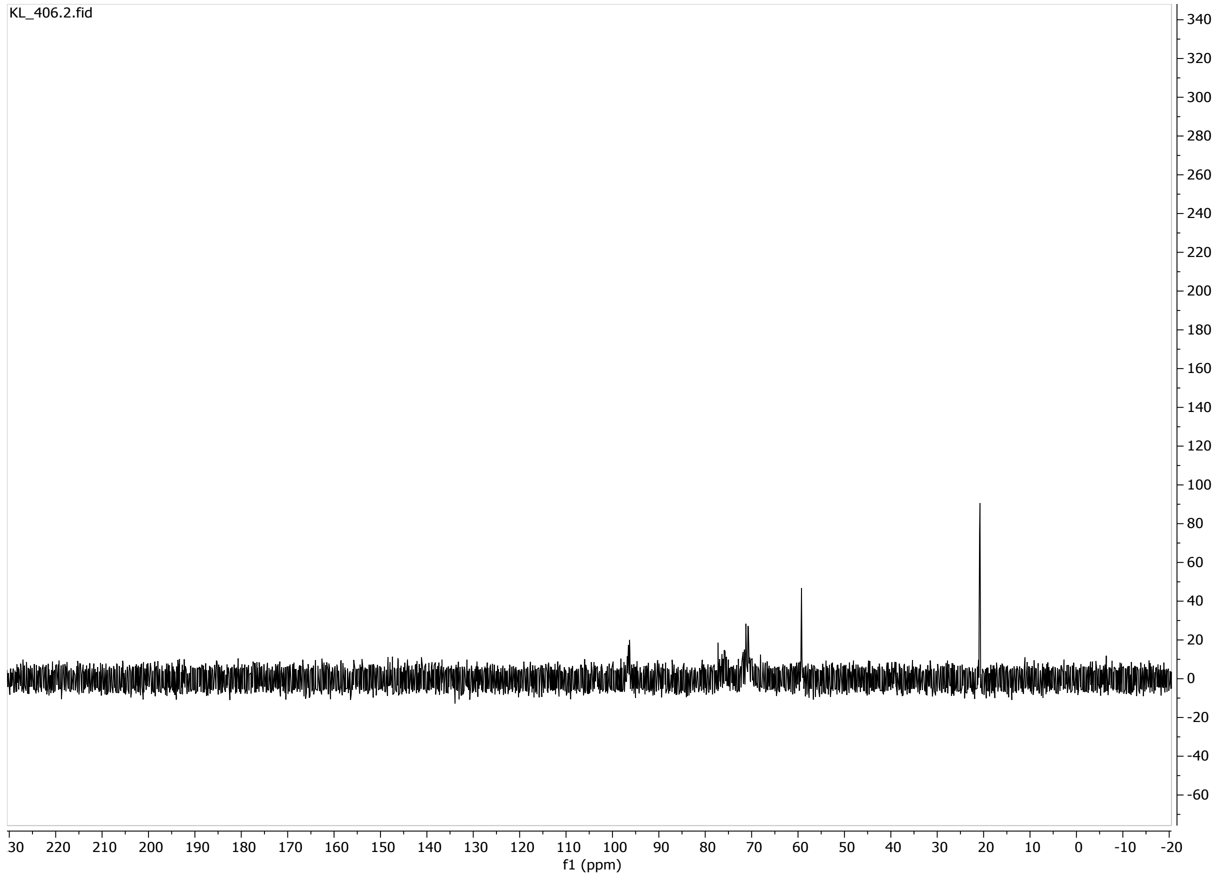

Fig. S54 ^{13}C-DEPT-101 MHz spectrum of **1e** in $CDCl_3$ at 25 °C

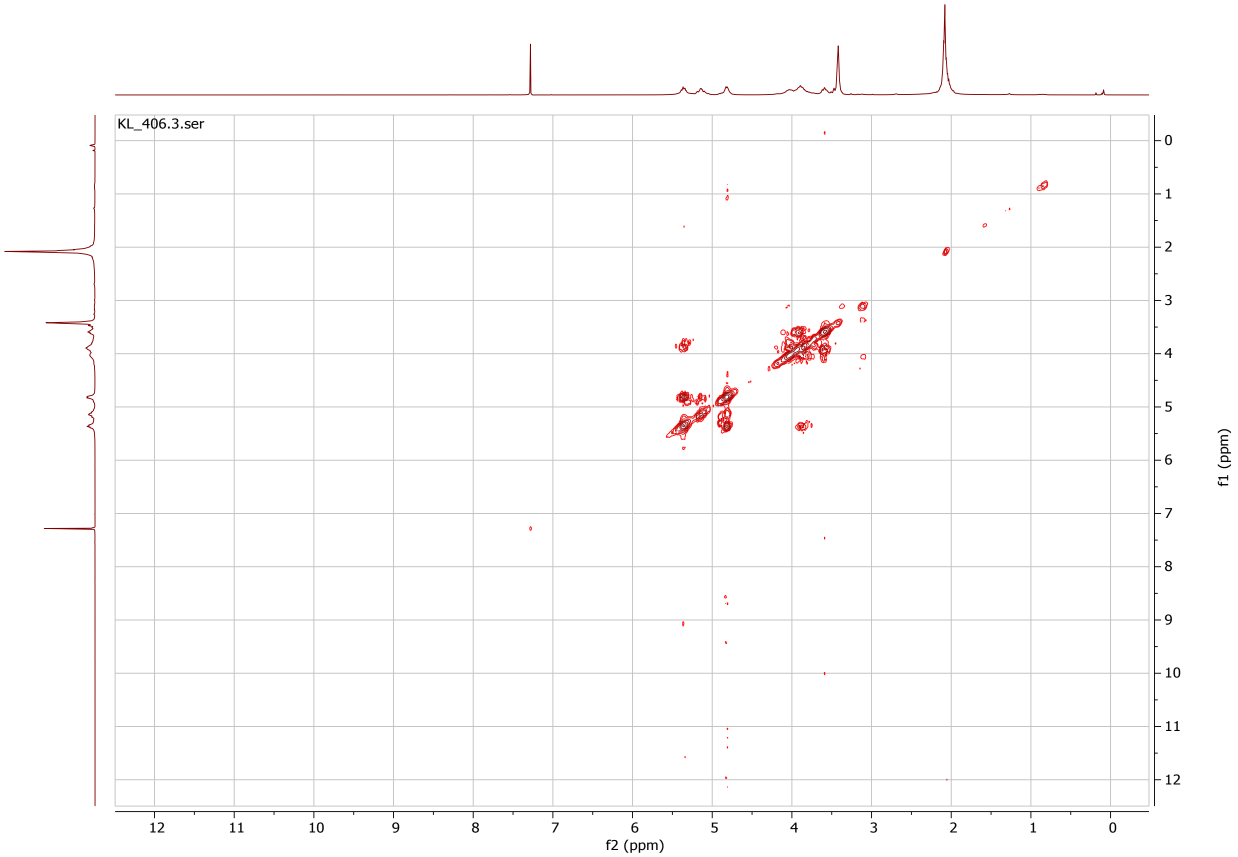

Fig. S55 2D-COSY spectrum of **1e** in $CDCl_3$ at 25 °C

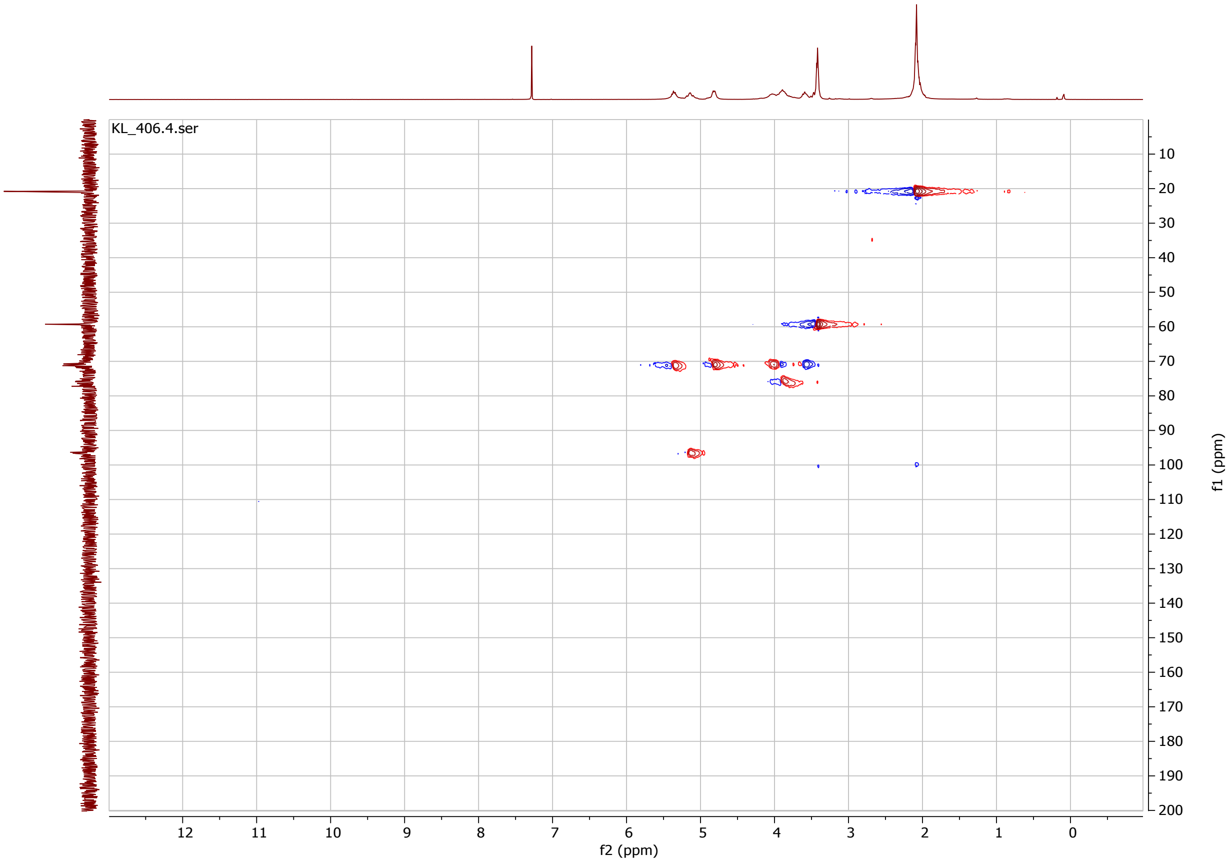

Fig. S56 2D-HSQC spectrum of **1e** in $CDCl_3$ at 25 °C

2^{A-G},3^{A-G}-Tetradeca-*O*-acetyl-6^{A}-bromo-6^{A}-deoxy-6^{B-G}-hexa-*O*-methyl-cyclomaltoheptaose (**3e**)

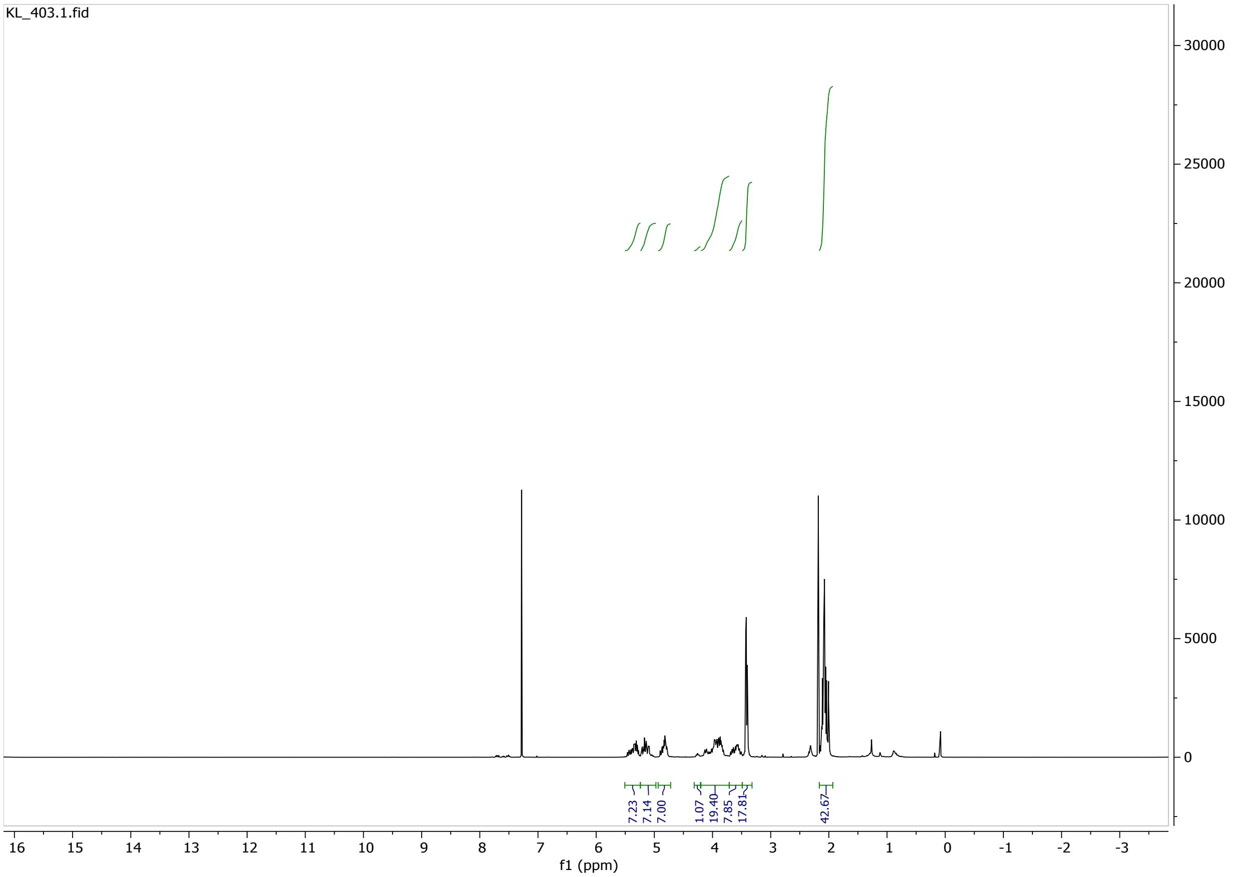

Fig. S57 ^{1}H-NMR-400 MHz spectrum of **3e** in $CDCl_3$ at 25 °C

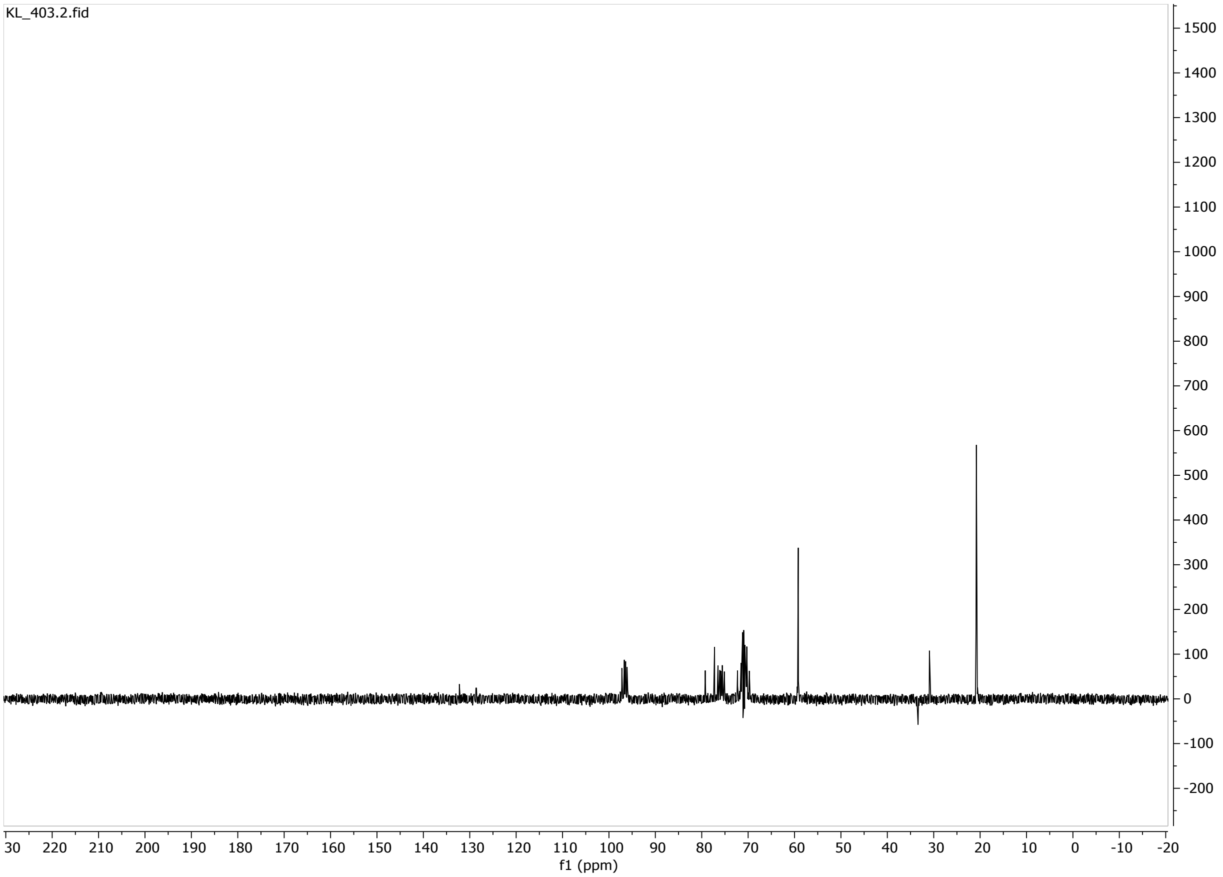

Fig. S58 ^{13}C-DEPT-101 MHz spectrum of **3e** in $CDCl_3$ at 25 °C

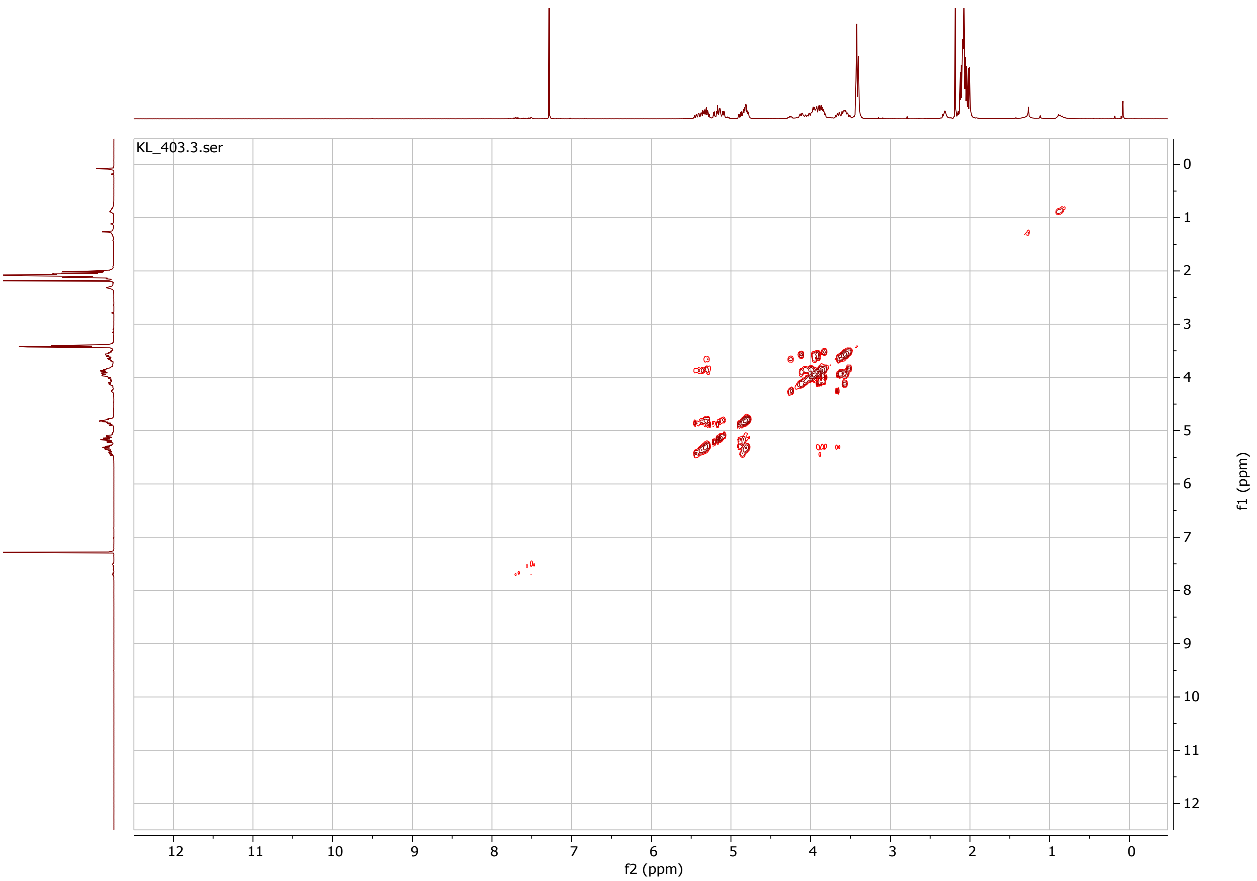

Fig. S59 2D-COSY spectrum of **3e** in $CDCl_3$ at 25 °C

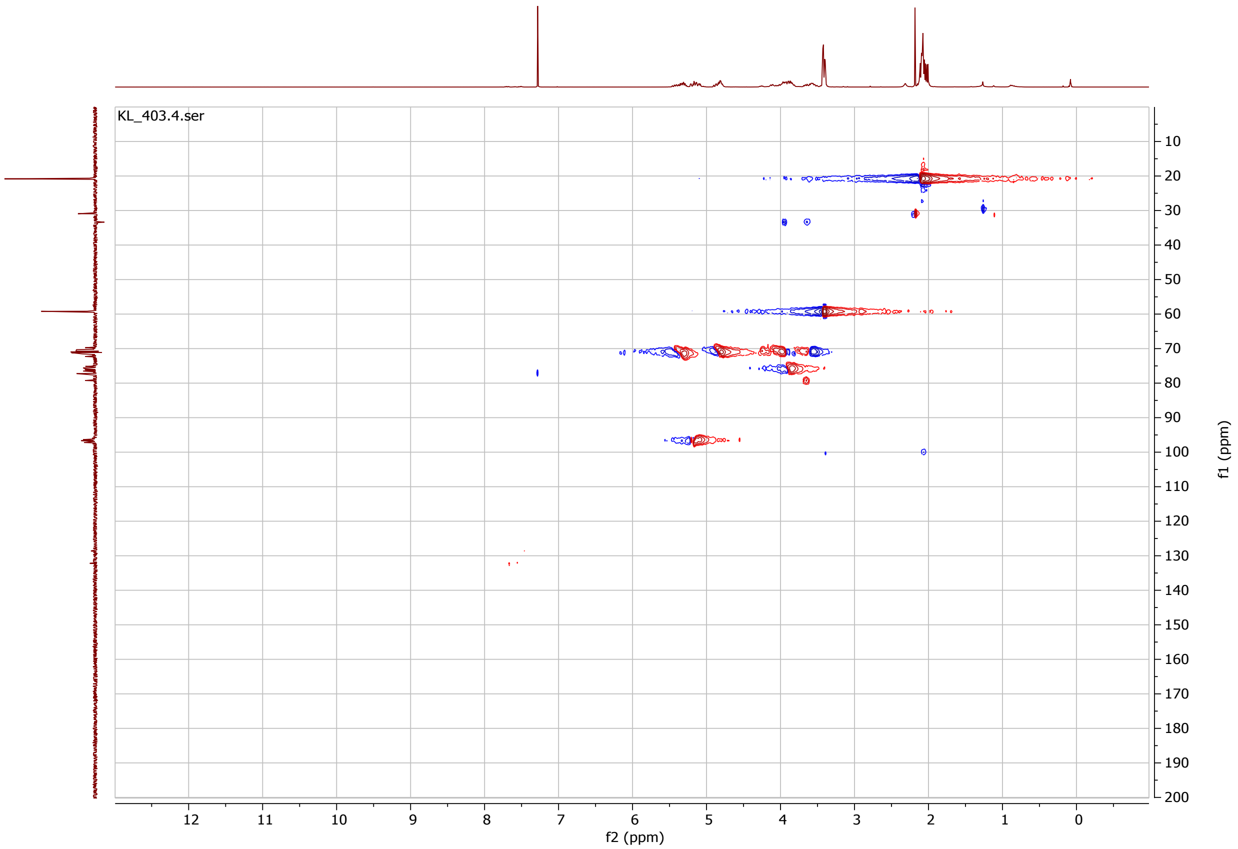

Fig. S60 2D-HSQC spectrum of **3e** in $CDCl_3$ at 25 °C

2^{A-G},3^{A-G}-Tetradeca-*O*-acetyl-6^{A}-deoxy-6^{A}-iodo-6^{B-G}-hexa-*O*-methyl-cyclomaltoheptaose (**4e**)

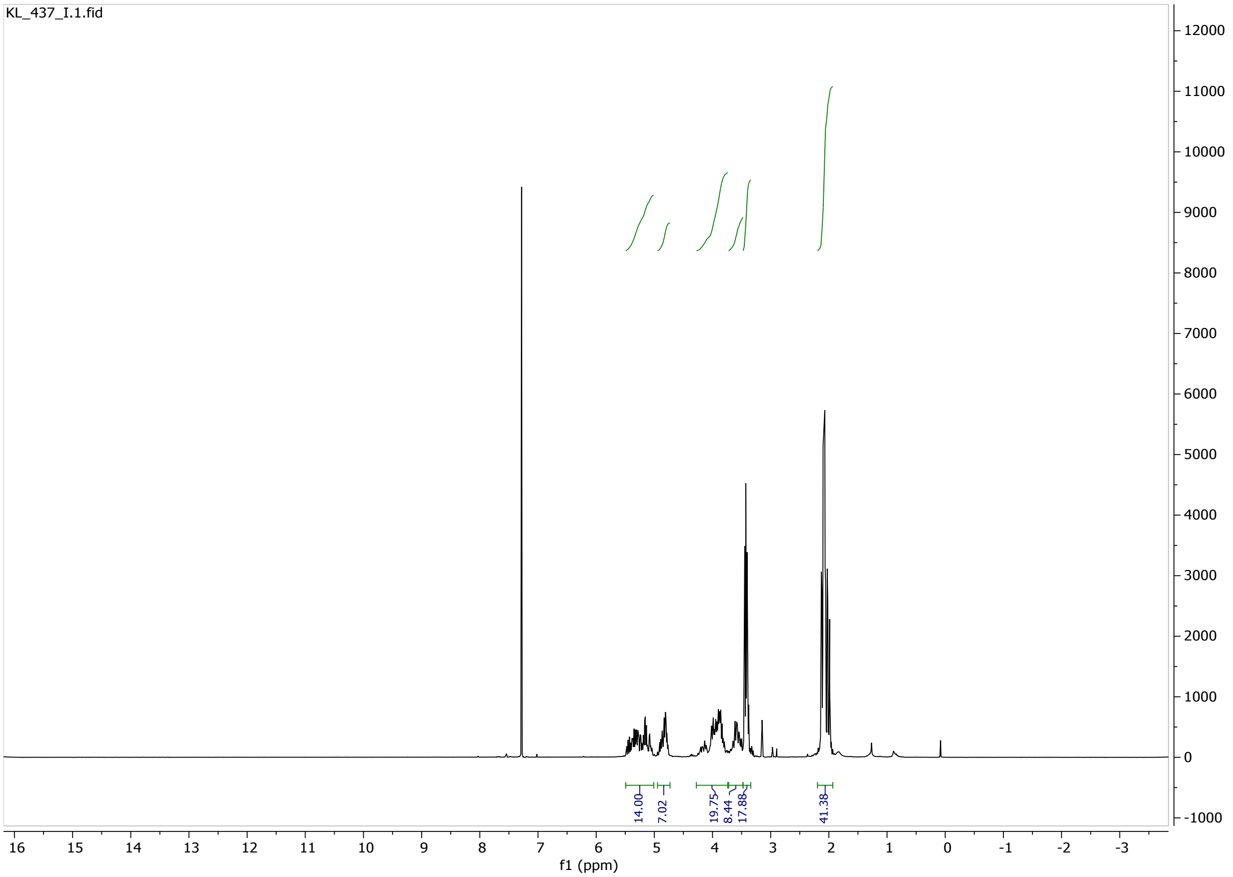

Fig. S61 ^{1}H-NMR-400 MHz spectrum of **4e** in $CDCl_3$ at 25 °C

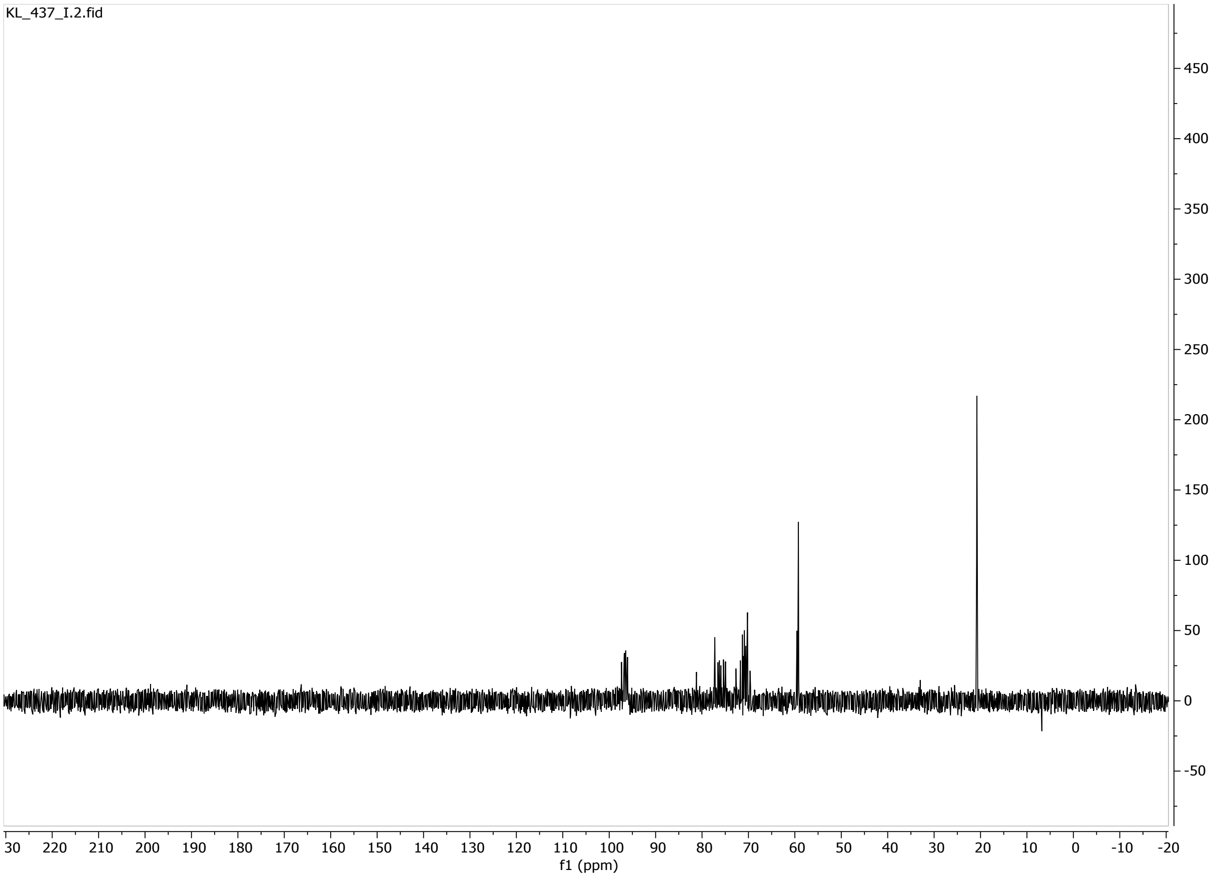

Fig. S62 ^{13}C-DEPT-101 MHz spectrum of **4e** in $CDCl_3$ at 25 °C

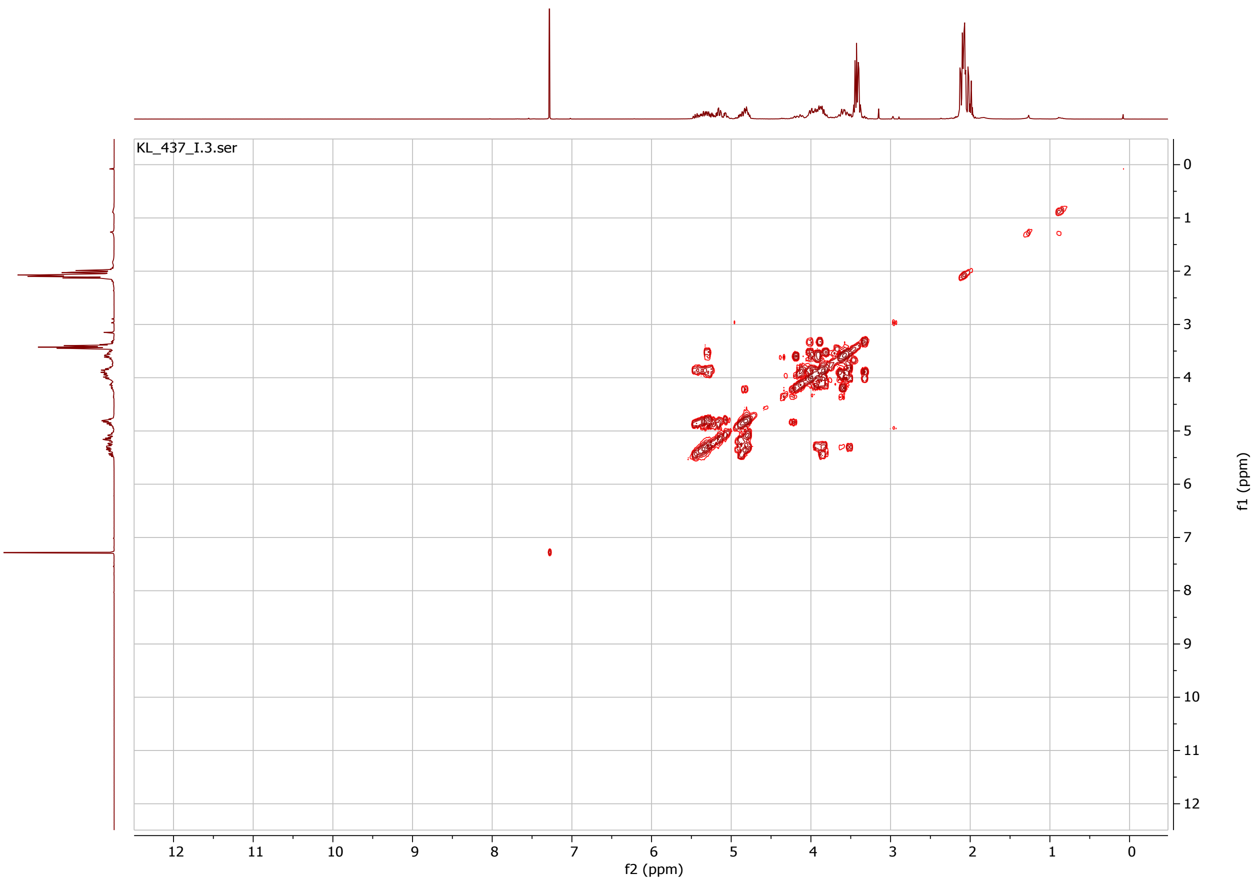

Fig. S63 2D-COSY spectrum of **4e** in $CDCl_3$ at 25 °C

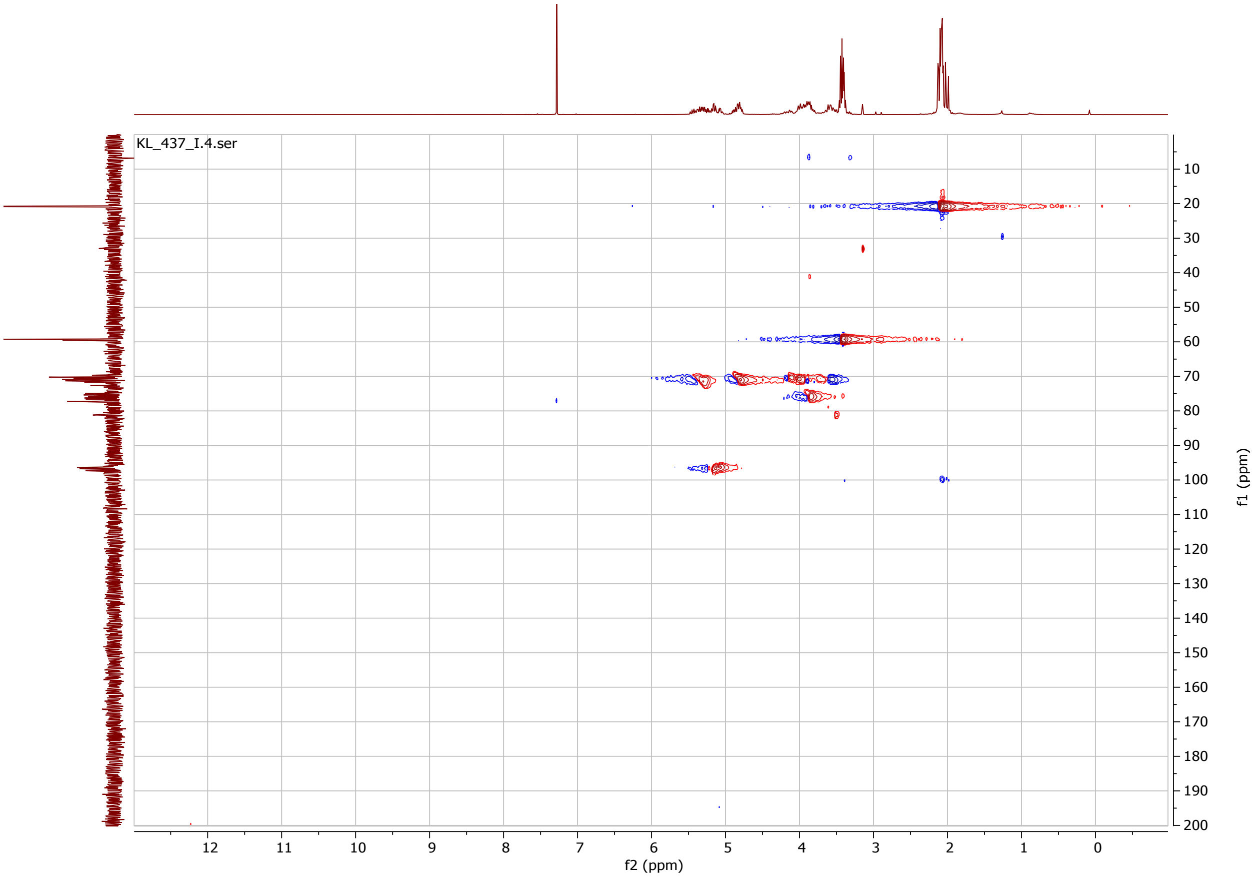

Fig. S64 2D-HSQC spectrum of **4e** in $CDCl_3$ at 25 °C
